# RNA/DNA Hybrid Interactome Identifies DXH9 as a Molecular Player in Transcriptional Termination and R-Loop-Associated DNA Damage

**DOI:** 10.1016/j.celrep.2018.04.025

**Published:** 2018-05-08

**Authors:** Agnese Cristini, Matthias Groh, Maiken S. Kristiansen, Natalia Gromak

**Affiliations:** 1 Sir William Dunn School of Pathology, University of Oxford, South Parks Road, Oxford OX1 3RE, UK

**Keywords:** R-loop, RNA/DNA hybrid, interactome, DHX9, DNA damage, PARP1, transcription, topoisomerase

## Abstract

R-loops comprise an RNA/DNA hybrid and displaced single-stranded DNA. They play important biological roles and are implicated in pathology. Even so, proteins recognizing these structures are largely undefined. Using affinity purification with the S9.6 antibody coupled to mass spectrometry, we defined the RNA/DNA hybrid interactome in HeLa cells. This consists of known R-loop-associated factors SRSF1, FACT, and Top1, and yet uncharacterized interactors, including helicases, RNA processing, DNA repair, and chromatin factors. We validate specific examples of these interactors and characterize their involvement in R-loop biology. A top candidate DHX9 helicase promotes R-loop suppression and transcriptional termination. DHX9 interacts with PARP1, and both proteins prevent R-loop-associated DNA damage. DHX9 and other interactome helicases are overexpressed in cancer, linking R-loop-mediated DNA damage and disease. Our RNA/DNA hybrid interactome provides a powerful resource to study R-loop biology in health and disease.

## Highlights

• Mass spectrometry identifies the RNA/DNA hybrid interactome in human cells
• Top RNA/DNA interactome candidate DHX9 promotes R-loop suppression
• DHX9 regulates transcriptional termination
• DHX9 interacts with PARP1 and prevents R-loop-associated DNA damage

## Graphical Abstract

**Figure:**
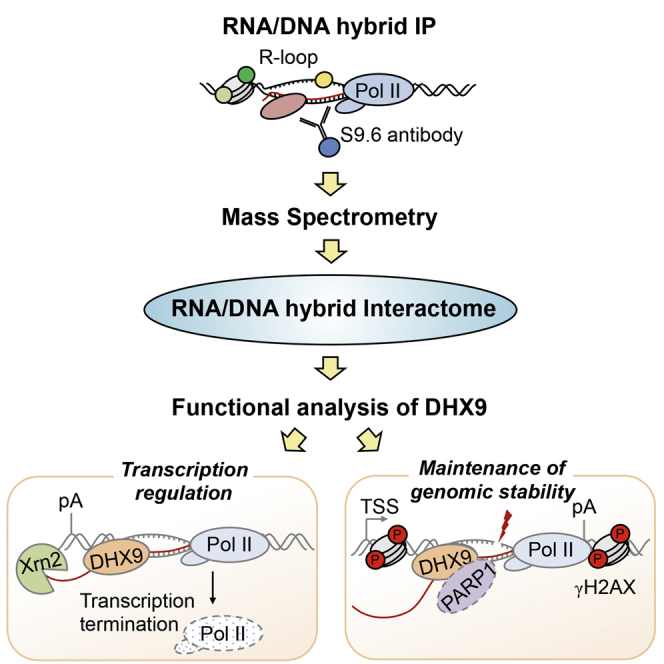

## Teaser

Cristini et al. use affinity purification and mass spectrometry to define the RNA/DNA interactome in human cells and validate it by revealing the role of DHX9 in transcriptional termination and R-loop-associated DNA damage.

## Introduction

R-loops consist of an RNA/DNA hybrid and a displaced non-template DNA strand. These structures are thermodynamically stable and can arise during transcription, where they contribute to gene regulation at multiple levels (Ginno et al., 2012, Skourti-Stathaki et al., 2011, Yang et al., 2014). They also are involved in immunoglobulin class switch recombination, DNA replication, and regulation of DNA and histone modifications (Aguilera and García-Muse, 2012, Skourti-Stathaki and Proudfoot, 2014).

Despite crucial biological processes associated with R-loops, many aspects of R-loop biology remain unclear. Which factors influence R-loop formation at different genomic locations? How are R-loop levels precisely controlled to allow for their beneficial functions while preventing detrimental effects from dysregulated R-loops? Failure to correctly control R-loop levels results in increased DNA damage and genome instability (Aguilera and García-Muse, 2012, Skourti-Stathaki and Proudfoot, 2014, Sollier and Cimprich, 2015) and aberrant transcriptional termination (Skourti-Stathaki et al., 2011, Skourti-Stathaki et al., 2014). Recent studies have implicated R-loops in the pathology of human diseases (Groh and Gromak, 2014). R-loops can form at expanded trinucleotide DNA repeats, leading to heterochromatin formation and transcriptional repression of genes associated with neurological disorders, including amyotrophic lateral sclerosis/frontotemporal dementia (ALS/FTD), Friedreich ataxia, and fragile X syndrome (Colak et al., 2014, Groh et al., 2014, Haeusler et al., 2014). A growing body of evidence also connects R-loops to processes that are deregulated in cancer, including DNA repair, replication, and gene expression of tumor-promoting genes (Bhatia et al., 2014, Boque-Sastre et al., 2015, Hatchi et al., 2015, Kotsantis et al., 2016, Schwab et al., 2015, Stork et al., 2016, Yang et al., 2014).

Genetic screens have been used to identify factors that regulate R-loop levels in yeast (Chan et al., 2014, Stirling et al., 2012, Wahba et al., 2011). However, these screens could not distinguish direct or indirect effects of these factors on R-loops, and the molecular mechanisms linking these factors to R-loop biology are still not understood fully. Human cells possess dedicated enzymes that can directly bind and regulate R-loop levels, including members of the RNase H family that specifically degrade the RNA in R-loops (Cerritelli and Crouch, 2009) and the helicase senataxin (SETX) that can unwind RNA/DNA hybrids (Skourti-Stathaki et al., 2011). It is interesting that mutations in RNase H and SETX lead to devastating neurological disorders, further underlining the importance of maintaining the correct R-loop balance in human cells (Groh et al., 2017, Groh and Gromak, 2014). Considering the complexity of RNA-processing reactions and the high abundance of non-coding RNA transcription, R-loop formation is a likely consequence that will require the availability of protein factors to directly control R-loop levels.

We used an unbiased approach to purify RNA/DNA hybrids and identify their associated proteins by mass spectrometry (MS) in human cells. This RNA/DNA hybrid interactome comprises 469 proteins, including helicases, RNA/DNA-binding proteins, and factors implicated in DNA damage. More important, we identify and validate factors previously implicated in R-loop biology, including SRSF1 and Top1, as well as candidate RNA/DNA hybrid interactors DHX9, PARP1, SAFB2, WHSC1, and DNA-PK. We demonstrate that a top candidate DHX9 helicase prevents R-loop accumulation *in vivo* and is required for transcriptional termination. We also describe a role of DHX9 in maintaining genome stability. DHX9 interacts with PARP1, and the loss of these proteins leads to R-loop accumulation, which triggers DNA damage. DHX9 and other RNA/DNA hybrid-interacting helicases are overexpressed in cancers, highlighting their potential role in the cancer transcriptional program. In conclusion, our data show that the RNA/DNA hybrid interactome reveals new RNA/DNA hybrid-interacting proteins and provides a link between fundamental aspects of R-loop biology and human disease.

## Results

### Design and Validation of the RNA/DNA Hybrid IP Method

**Figure 1:**
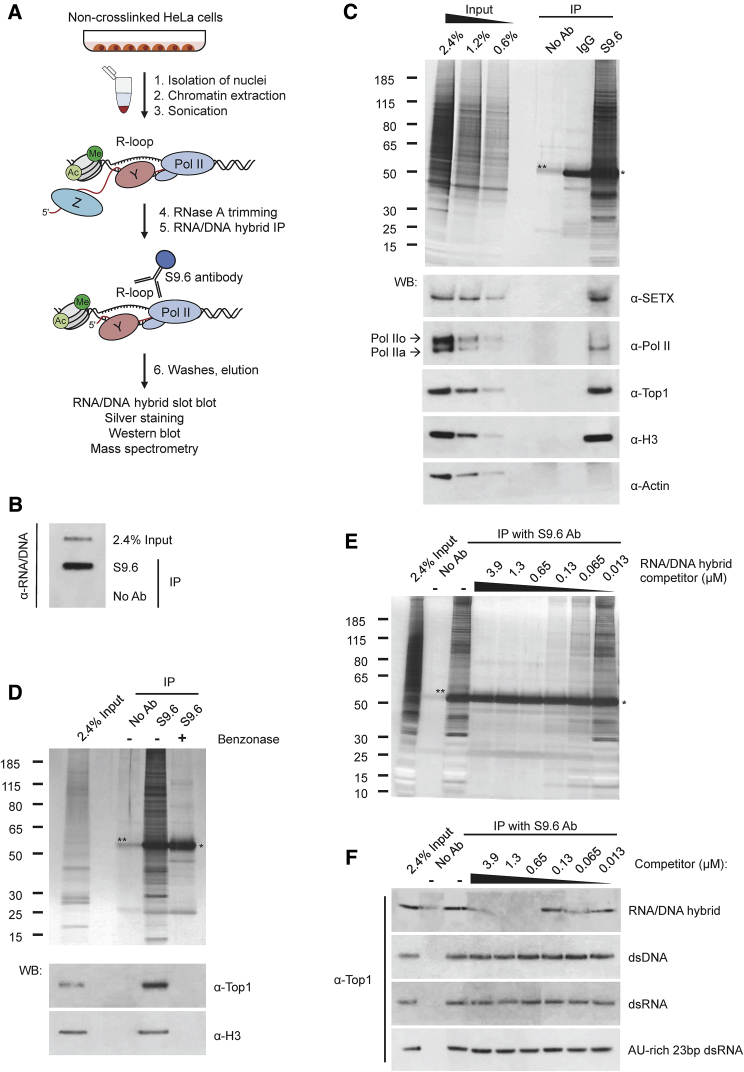
Design of RNA/DNA Hybrid IP Method in HeLa Cells (A) RNA/DNA hybrid IP workflow in HeLa cells. (B) RNA/DNA hybrid slot blot with S9.6 antibody. (C) Top: silver stain of RNA/DNA hybrid IP. No antibody and isotype-matched IgG2a antibody were used as controls. Bottom: western blot of RNA/DNA hybrid IP using indicated antibodies. Arrows indicate hypophosphorylated (IIa) and hyperphosphorylated (IIo) forms of Pol II. Triple amounts of input and IP samples were loaded for SETX. (D) Top: silver stain of RNA/DNA hybrid IP following benzonase treatment. Bottom: western blot of RNA/DNA hybrid IP, probed with Top1 and H3 antibodies. (E) Silver stain of RNA/DNA hybrid IP in the presence of RNA/DNA hybrid competitor. (F) Western blot for Top1 of RNA/DNA hybrid IP with indicated synthetic competitors. (C–E) ^∗^, indicates the heavy chain of S9.6 and IgG2a antibodies. ^∗∗^, indicates BSA, used to block protein A dynabeads. See also Figure S1.

To identify RNA/DNA hybrid-interacting factors in an unbiased way, we developed an affinity purification approach, using the S9.6 antibody, which specifically recognizes RNA/DNA hybrids with 0.6 nM affinity (Boguslawski et al., 1986, Phillips et al., 2013). Nuclear extracts were prepared from HeLa cells and sonicated before immunoprecipitation (IP) to minimize copurification of unspecific proteins (Figures 1A and S1A). Non-crosslinked cells were used because crosslinking reagents could induce R-loops (Schwab et al., 2015), preventing the identification of bona fide R-loop interactors. IP using the S9.6 antibody was carried out in the presence of RNase A to reduce unspecific RNA-mediated interactions and avoid S9.6 recognition of double-stranded RNA (dsRNA) (Figure S1B). RNase A treatment before IP did not affect RNA/DNA hybrid IP results, suggesting that R-loops are not formed artificially during the extraction procedure (data not shown). We verified that the RNA/DNA hybrid IP enriches for RNA/DNA hybrids using the RNA/DNA hybrid slot blot, which quantitatively detects both endogenous and synthetic RNA/DNA hybrids (Figures 1B and S1C). Silver staining of immunoprecipitated RNA/DNA hybrid-interacting proteins revealed a complex mixture of proteins, which differed from the nuclear input material (Figure 1C) or IP carried out with antibody-recognizing cap-binding protein 80 (CBP80) (Figure S1D). More important, little protein was detected in no antibody or matched isotype immunoglobulin G2 (IgG2) negative IP controls. These results suggest that the RNA/DNA hybrid IP enriches a unique set of interacting proteins.

To test possible cross-reactivity of the S9.6 antibody with proteins, nuclear extracts were treated with benzonase, which degrades all forms of nucleic acids, including RNA/DNA hybrids, without affecting proteins (Figure S1E). Benzonase caused a loss of immunoprecipitated proteins (Figure 1D), confirming that RNA/DNA hybrid IP proteins are associated with nucleic acids recognized by the S9.6 antibody.

Next, we validated our IP procedure by testing copurification of proteins already implicated in R-loop biology. In particular, we detected the RNA/DNA helicase SETX, which resolves R-loops at termination regions (Figure 1C, bottom) (Hatchi et al., 2015, Skourti-Stathaki et al., 2011) and Top1 (El Hage et al., 2010, Tuduri et al., 2009). In line with cotranscriptional R-loop formation, RNA polymerase II (Pol II) also was detected. As an integral part of chromatin, RNA/DNA hybrids interacted with histone H3 but not with actin.

To verify that proteins identified in the RNA/DNA hybrid IP specifically bind RNA/DNA hybrids, we added synthetic 15–23 bp competitors during the IP procedure. When 15-bp RNA/DNA hybrid with 0.54 nM affinity for S9.6 antibody (Phillips et al., 2013) was added, a significant competition was observed for most concentrations, as demonstrated by a loss of copurified proteins, including Top1 and H3 (Figures 1E and 1F and S1F). In contrast, the addition of corresponding dsDNA, dsRNA, and 23 bp AU-rich dsRNA, which has low affinity for S9.6 *in vitro* (Phillips et al., 2013), did not affect RNA/DNA hybrid IP efficiency (Figures 1F and S1F–S1H). Supporting the specificity of our method, we did not detect the loss of proteins in the CBP80 IP in the presence of each competitor (Figure S1D).

In conclusion, even though a low affinity of S9.6 antibody with dsRNA in mild buffer conditions has been reported (Phillips et al., 2013), our stringent purification procedure and the use of RNase A ensures that the RNA/DNA hybrid IP method exhibits high specificity and efficiently enriches for proteins associated with RNA/DNA hybrids.

### Characterization of the RNA/DNA Hybrid Interactome

**Figure 2:**
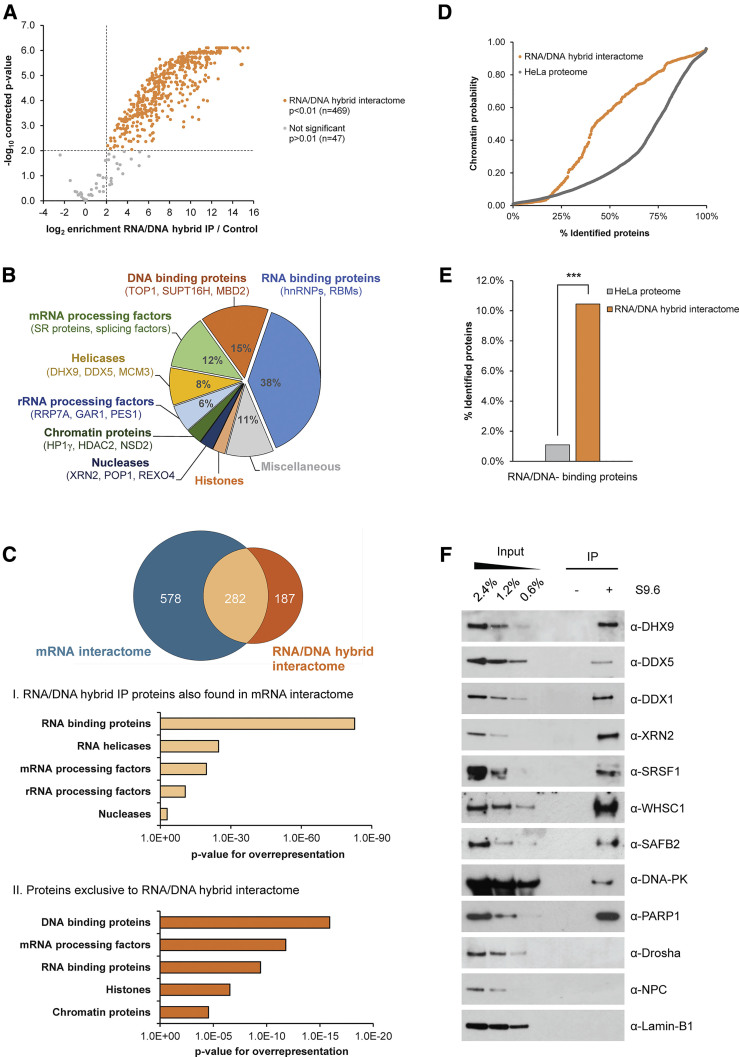
Characterization of the RNA/DNA Hybrid Interactome (A) Volcano plot displaying MS results of three biological replicates of RNA/DNA hybrid IP. Averaged log_2_ ratios between RNA/DNA hybrid IP and control IP (with addition of 1.3 μM synthetic RNA/DNA hybrid competitor) are plotted against their Benjamini-Hochberg-corrected −log_10_ p values across all replicates using a moderated t test. Proteins significantly enriched in RNA/DNA hybrid IP are in orange (p < 0.01) and constitute the RNA/DNA hybrid interactome. Proteins identified with p > 0.01 are in gray. Dashed lines indicate the significance cutoffs (log_2_ enrichment > 2 and –log_10_ > 2). (B) Protein classes overrepresented in RNA/DNA hybrid interactome (corrected p < 0.05, Fisher’s exact test). Representative proteins are given in brackets. (C) Top: overlap between mRNA interactome and RNA/DNA hybrid interactome in HeLa cells. Bottom: common proteins enriched in both RNA/DNA hybrid and mRNA interactomes (I) and proteins unique to the RNA/DNA hybrid interactome (II). The x axis indicates statistical significance of overrepresentation. (D) The chromatin probability analysis of 7,635 HeLa proteins (HeLa proteome) and RNA/DNA hybrid interactome. (E) Enrichment for proteins known to bind both RNA and DNA in the RNA/DNA hybrid interactome compared to the HeLa proteome (p < 1.6 × 10^−28^, Fisher’s exact test). (F) Validation of RNA/DNA hybrid interactors using western blot, probed with indicated antibodies. Drosha, NPC, and Lamin B1 are negative controls. (−) IP lane corresponds to control IP with IgG2a antibody. See also Figure S2.

We then developed a proteomic pipeline based on label-free quantitative MS to identify proteins associated with RNA/DNA hybrids in HeLa cells. To achieve the highest specificity, we compared protein enrichment in RNA/DNA hybrid IP to a control IP with S9.6 antibody in the presence of 1.3 μM RNA/DNA hybrid competitor. In total, 846 proteins were identified with high reproducibility (*r* > 0.77) in three biological replicates using MaxQuant software (Figure S2A) (Cox and Mann, 2008). Next, by using a moderated t test, we identified 469 candidate factors significantly enriched in the RNA/DNA hybrid versus control IP (Smyth, 2004). These proteins represent the RNA/DNA hybrid interactome (Figure 2A, orange data points). Analysis of our MS data with an independent approach based on the normalized spectral index (SIN) (Trudgian et al., 2011) found a strong correlation between the two methods (*r* = 0.796), confirming the robustness of the analysis (Figures S2B and S2C). We subdivided the RNA/DNA hybrid interactome into three classes according to their statistical significance: class I (top 25%), class II (next 50%), and class III (bottom 25%) (Figure S2D). A representative list of identified proteins, encompassing a variety of cellular functions, is provided in Table S2.

As expected, the RNA/DNA hybrid interactome almost exclusively contained nuclear proteins (Figure S2E). However, we observed no correlation between their cellular abundance (Geiger et al., 2012) and enrichment in the RNA/DNA hybrid interactome (Figure S2F), indicating that the RNA/DNA hybrid IP enriches for a specific subset of the proteome. Indeed, protein classes involved in RNA metabolism, such as heterogeneous nuclear ribonucleoproteins (hnRNPs), SR proteins and splicing factors, DNA-binding proteins (including Top1 and FACT), and chromatin-associated proteins, were overrepresented (Figure 2B). Notably, we detected many helicases acting on RNA (DDX5 and DHX9) or DNA (MCM3) and a high enrichment of RNA/DNA-binding domains, including RNA recognition motif (RRM); K homology (KH); double-stranded RNA-binding domains (dsRBDs); and SAF-A/B, acinus, and PIAS (SAP) (Figure S2G). This suggests that the RNA/DNA hybrid IP detects direct interactions rather than secondary interactions mediated by protein-protein binding.

We also investigated whether the RNA/DNA hybrid interactome exhibits additional characteristics. Comparison of the RNA/DNA hybrid interactome to the HeLa mRNA interactome (Castello et al., 2012) showed that despite an overlap between the two datasets, a significant part of the RNA/DNA hybrid interactome (187 proteins) is unique (Figure 2C, top). While proteins common to both interactomes are enriched for RNA-related functions, factors unique to the RNA/DNA hybrid interactome are involved in DNA and chromatin biology (Figure 2C, bottom). Consequently, we also detected an overall enrichment for proteins with high chromatin probability (Figure 2D) (Kustatscher et al., 2014) and proteins capable of binding to both RNA and DNA (Figure 2E) (Hudson and Ortlund, 2014) compared to the HeLa proteome. More important, there was no correlation between protein abundance in the RNA/DNA hybrid interactome and chromatin probability, demonstrating that our method enriches for a specific subset of proteins over the chromatin background (Figure S2H). It is interesting that the RNA/DNA hybrid interactome also was enriched for factors mediating genome stability (Paulsen et al., 2009) (Figure S2J), whereas other abundant protein classes were depleted (Figure S2I). Finally, consistent with the literature, we found a number of proteins already implicated in R-loop biology in human cells, including Top1 (El Hage et al., 2010, Tuduri et al., 2009), SRSF1 (Li and Manley, 2005, Tuduri et al., 2009), FACT (Herrera-Moyano et al., 2014), and some human counterparts of factors regulating R-loops in yeast, including ALY/REF (yeast YRA1) (Gavaldá et al., 2013) and DDX39B (yeast Sub2) (Gómez-González et al., 2011) (Table S3).

### RNA/DNA Hybrid Interactome Uncovers New Candidates Involved in R-Loop Biology *In Vivo*

Next, we experimentally validated proteins identified in all three classes of the RNA/DNA hybrid interactome by western blotting (class I: DHX9, DDX5, WHSC1, SAFB2; class II: DDX1, XRN2, DNA-PK, PARP1; class III: SRSF1) (Figure 2F). In particular, we confirmed a number of proteins already implicated in R-loop biology, including the 5′-3′ exonuclease XRN2, previously associated with R-loop-mediated transcription termination (Skourti-Stathaki et al., 2011) and SRSF1 (Li and Manley, 2005, Tuduri et al., 2009). In addition, we validated candidates with previously unreported function in R-loop metabolism *in vivo*, including DHX9, DDX5, WHSC1, SAFB2, DNA-PK, and PARP1. We also confirmed the absence of abundant nuclear proteins, including Drosha, Lamin B1, and nuclear pore complex (NPC).

**Figure 3:**
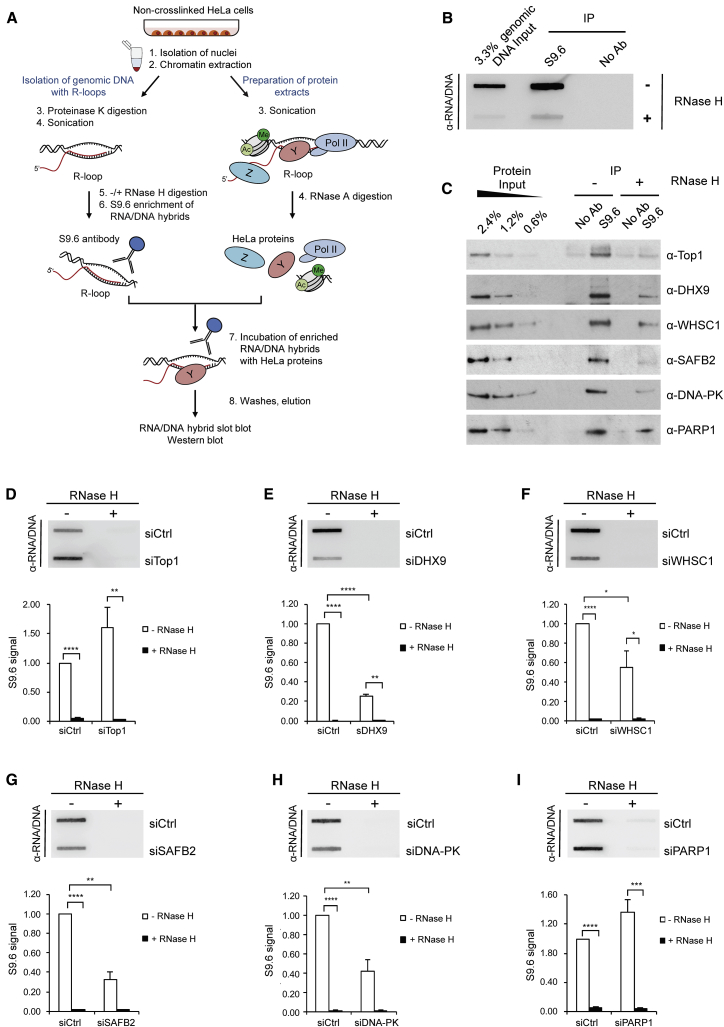
Validation of New RNA/DNA Hybrid Interactome Candidates (A) Workflow of RNA/DNA hybrid IP with RNase H digestion. (B and C) HeLa genomic DNA input was either treated (+) or not (−) with RNase H before enrichment for RNA/DNA hybrids with the S9.6 antibody. Genomic RNA/DNA hybrids were incubated with nuclear extracts depleted for RNA/DNA hybrids with RNase A, followed by S9.6 IP. RNA/DNA hybrid slot blot (B) and western blot of RNA/DNA hybrid IP, probed with indicated antibodies (C). (D–I) Genomic DNA from HeLa cells transfected with control (siCtrl) or indicated siRNAs was treated with RNase H. siTop1 (D), siDHX9 #1 (E), siWHSC1 (F), siSAFB2 (G), siDNA-PK (H), siPARP1 (I) were used. Top: RNA/DNA hybrid slot blot. Bottom: quantification of S9.6 signal. Values are normalized to the siCtrl and represent the means ± SEMs, n ≥ 3. See also Figure S3.

To confirm the specificity of these RNA/DNA hybrid interactors, we performed a modified version of the RNA/DNA hybrid IP with RNase H treatment (Figure 3A). The genomic DNA was extracted from HeLa cells and enriched for RNA/DNA hybrids with the S9.6 antibody. These genomic RNA/DNA hybrids were then incubated with HeLa nuclear extracts, depleted for RNA/DNA hybrids by treatment with a high concentration of RNase A, followed by IP of RNA/DNA-binding proteins with the S9.6 antibody (Figures 3B and 3C and S3A). All new R-loop-interacting candidates, including Top1, used as a positive control, can bind genomic RNA/DNA hybrids (Figure 3C). In contrast, treatment of the genomic DNA with RNase H strongly decreased the amount of copurified proteins, which correlated with the decrease in the RNA/DNA hybrid signal on the slot blot (Figures 3B and 3C).

To further investigate the involvement of selected candidates in R-loop biology, we performed slot blot analyses upon small interfering RNA (siRNA)-mediated depletion of these candidates (Figures S3C–3H). We observed that the knockdown of DHX9, WHSC1, SAFB2, and DNA-PK decreases the global level of RNA/DNA hybrids (Figures 3E–3H and S3B), whereas depletion of PARP1 and Top1 triggers an increase (Figures 3D and 3I). Moreover, the S9.6 signal was abolished by RNase H treatment, indicating that it is specific to RNA/DNA hybrids (Figures 3D–3I). Even though the mechanistic basis of the global R-loop changes requires further investigation, these results demonstrate that the RNA/DNA hybrid interactome uncovers new factors involved in R-loop biology.

### DHX9 Promotes Transcriptional Termination

**Figure 4:**
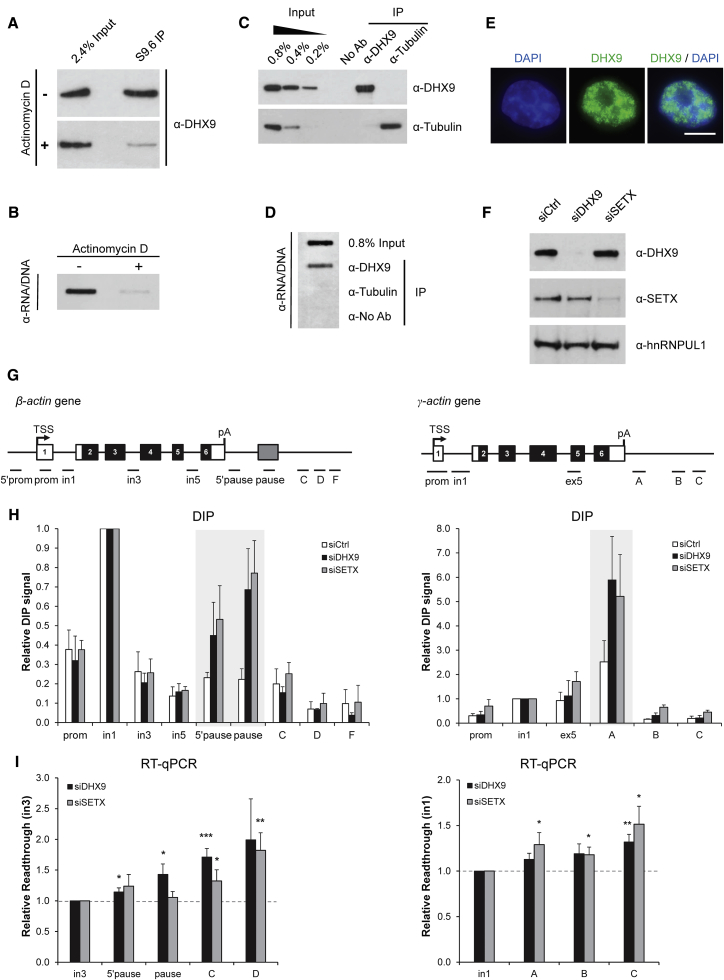
DHX9 Promotes Transcriptional Termination (A and B) Western blot probed with DHX9 antibody (A) and RNA/DNA hybrid slot blot (B) of RNA/DNA hybrid IP from cells treated (+) or not (−) with actinomycin D. (C and D) Western blot probed with DHX9 and tubulin antibodies (C) and RNA/DNA hybrid slot blot (D) of IPs carried out with DHX9 and tubulin antibodies. (E) IF analysis of DHX9 (green). DAPI (blue) depicts nuclei. Scale bar, 10 μm. (F) Western blot of HeLa protein extracts, treated with control, DHX9 #1, and SETX siRNAs and probed with indicated antibodies. hnRNPUL1 is a loading control. (G) Diagram of *β*-*actin* (left) and *γ*-*actin* (right) genes depicting exons (black), UTRs (white), transcriptional start site (TSS), termination region (gray), and qPCR amplicons. (H) DIP in HeLa cells, treated with control, DHX9 #1 and SETX siRNAs, on *β*-*actin* (left) and *γ*-*actin* (right) genes. Values are normalized to in1. (I) Read-through transcription analysis of *β*-*actin* (left) and *γ*-*actin* (right) genes in HeLa cells treated with control, DHX9 #1, and SETX siRNAs, using RT-qPCR. Values are normalized to *β-actin* in3 and *γ*-*actin* in1. (H–I) Bars, means ± SEMs, n ≥ 3. See also Figure S4.

We detected several helicases enriched in the RNA/DNA interactome, suggesting that this protein class may play yet uncharacterized roles in R-loop biology. Therefore, we decided to further investigate the R-loop-associated function of a top validated candidate, DHX9. DHX9 has been shown to possess RNA/DNA helicase activity *in vitro* (Chakraborty and Grosse, 2011). To establish whether DHX9 associates with RNA/DNA hybrids *in vivo*, we confirmed the specificity of this interaction biochemically. RNA/DNA hybrid IP carried out with benzonase or RNase H treatments significantly reduced DHX9-RNA/DNA hybrid interaction (Figures S4A and 3C). Cell treatment with the transcriptional inhibitor actinomycin D also dramatically reduced DHX9-RNA/DNA hybrid interaction, suggesting that this interaction is transcription dependent (Figures 4A and 4B). Furthermore, DXH9-RNA/DNA hybrid interaction was prevented by synthetic RNA/DNA hybrid competitor but not by corresponding dsDNA, dsRNA, and AU-rich dsRNA competitors (Figure S4B). RNA/DNA hybrids also co-immunoprecipitated (coIP) with DHX9 but not with tubulin or no antibody control IPs (Figures 4C and 4D). These experiments confirm that DHX9 interacts with RNA/DNA hybrids *in vivo*.

We further examined the R-loop-associated biological functions of DHX9 *in vivo*. In line with its potential function in R-loop biology, endogenous DHX9 is predominantly localized throughout the nucleus (Figure 4E), and it is enriched at promoter-proximal regions of *β-actin* and *γ-actin* genes, as demonstrated by chromatin immunoprecipitation (ChIP) (Figures S4C and S4D). Therefore, we examined the R-loop profile of the *β-actin* and *γ-actin* genes in DHX9-depleted cells, using DNA/RNA immunoprecipitation (DIP) (Skourti-Stathaki et al., 2011). As a positive control, we also depleted SETX, which resolves R-loops at the termination regions of the *β-actin* gene (Skourti-Stathaki et al., 2011). R-loops are enriched over the promoter-proximal and termination regions of the *β-actin* (in1, pause, and 5′ pause) (Skourti-Stathaki et al., 2011) and *γ-actin* (in1, A) genes (Figures 4G and 4H). R-loops were increased over the termination regions of both genes in DHX9- and SETX-depleted cells (Figures 4F–4H and S4E and S4F), and the DIP signal was sensitive to RNase H treatment (Figure S5E). These data suggested that similar to SETX, DXH9 promotes R-loop suppression at termination regions.

Next, we investigated whether the function of DHX9 in R-loop metabolism in poly(A)-proximal regions of *β-actin* and *γ-actin* genes can affect their transcriptional termination, as previously shown for SETX (Skourti-Stathaki et al., 2011). Similar to SETX, DHX9 depletion caused an increase in the amount of read-through transcripts downstream of the poly(A) signal (Figure 4I) and stabilization of Pol II over the transcription termination region for both genes (Figure S4G). These data pointed toward a role for DHX9 in R-loop suppression during transcriptional termination.

### DHX9 Depletion Triggers R-Loop Accumulation in Response to CPT

Defects in transcription termination resulting from SETX or XRN2 depletion have been associated with R-loop accumulation and R-loop-driven genome instability (Hatchi et al., 2015, Morales et al., 2016). Because DHX9-depleted cells exhibit a termination defect (Figures 4H and 4I), we examined the potential role of DHX9 in preventing R-loop-associated DNA damage. For these studies, we used the Top1 inhibitor camptothecin (CPT), which is known to promote R-loop accumulation and replication- and transcription-associated DNA damage caused by unresolved DNA supercoiling (Marinello et al., 2016, Sordet et al., 2009). CPT treatment induced DNA damage, as indicated by the phosphorylation of histone variant H2AX (γH2AX) and R-loop accumulation as measured by immunofluorescence (IF) with the S9.6 antibody (Figures S5A and S5B).

**Figure 5:**
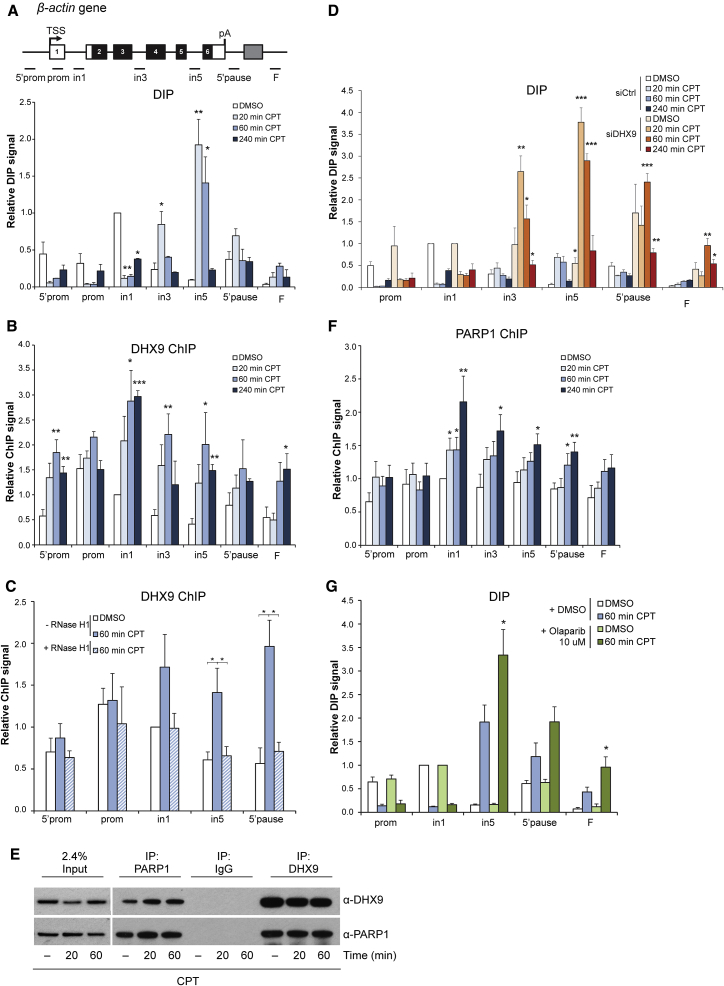
DHX9 Depletion Triggers R-Loop Accumulation in Response to CPT (A, B, and F) Diagram of *β*-*actin* gene (A, top), DIP (A, bottom), DHX9 ChIP (B), and PARP1 ChIP (F) in HeLa cells, treated with CPT for the indicated time, on *β*-*actin* gene. Values are relative to in1 in the DMSO sample. (C) DHX9 ChIP in HEK293T cells, transfected with FLAG (−RNase H1) or RNase H1 (+RNase H1) and treated with CPT for 60 min. Values are relative to in1 −RNase H1 in the DMSO sample. (D) DIP in HeLa cells, transfected with control (shades of blue) or DHX9 #1 (shades of red) siRNA and treated with CPT for indicated time, on the *β-actin* gene. Values are relative to in1 for each siRNA. The p-value is calculated for the siDHX9 versus the siCtrl sample. (E) Western blot of IgG2a (negative control), PARP1, and DHX9 IPs in HeLa cells treated with CPT and probed with indicated antibodies. Left: input, right: IP. (G) DIP in HeLa cells, treated with DMSO (shades of blue) or Olaparib (shades of green) before addition of CPT for 60 min, on the *β-actin* gene. Values are relative to in1 for DMSO and Olaparib. The pvalue is calculated for the Olaparib + CPT versus the DMSO + CPT samples. (A–D, F, and G) Bars, means ± SEMs, n ≥ 3. See also Figures S5 and S6.

Next, we examined the R-loop response following CPT treatment on the *β-actin* gene (Figure 5A). CPT triggered a reduction of R-loops over the prom and in1 regions, which correlated with transcriptional downregulation detected by Pol II ChIP (Figure S5C). This is in line with previously reported effects of CPT on Pol II transcription (Khobta et al., 2006). In contrast, CPT enhanced R-loop formation over in3, in5, and 5′ pause regions (Figure 5A). This coincided with the induction of γH2AX (Figure S5D). It is interesting that R-loops were most dramatically induced after 20 min, followed by a steady reduction, reaching the basal level at ∼240 min. These striking kinetics suggest that CPT-induced R-loops can be resolved efficiently by cellular factors. Therefore, we investigated the response of DHX9 to CPT-induced R-loop accumulation. ChIP analysis showed that despite a transcriptional downregulation (Figure S5C), DHX9 is enriched over the *β-actin* gene in response to CPT, and this binding is R-loop dependent because it is abolished by RNase H1 overexpression (Figures 5B and 5C). To test whether DHX9 is involved in the suppression of CPT-induced R-loops, we assessed R-loop levels in DHX9-depleted cells. In the absence of DHX9, CPT led to a dramatic increase in R-loops over in3, in5, and 5′ pause *β-actin* regions (Figure 5D). RNase H treatment confirmed that CPT-induced DIP peaks were specific (Figure S5E). Taken together, these results suggested that DHX9 is recruited to the *β-actin* gene to suppress CPT-induced R-loops.

DHX9 has been identified in MS screens for PARP1 and poly(ADP-ribos)ylation (PARylation) interactors (Gagné et al., 2012, Isabelle et al., 2010). We identified PARP1 in the RNA/DNA hybrid interactome by MS and validated its interaction with RNA/DNA hybrids *in vivo* (Figures 2F and 3C). Furthermore, we observed an increase in the global level of R-loops in PARP1-depleted cells (Figure 3I). Next, we examined whether PARP1 is involved in R-loop-driven DNA damage, similar to DHX9. CoIP experiments showed that endogenous DHX9 and PARP1 proteins interact in both untreated and CPT-treated cells (Figure 5E). Similar to DHX9, PARP1 was recruited to the *β-actin* gene in response to CPT (Figure 5F). Moreover, the CPT-induced R-loop signal was increased over in5 and 3′ end of the *β-actin* gene upon PARP inhibition with Olaparib (10 and 0.1 μM) or PARP1 depletion (Figures 5G and S6A–S6C). However, PARP inhibition did not further increase the R-loop signal on the in5 of the *β-actin* gene in DHX9-depleted cells, suggesting that DHX9 and PARP1 act in the same pathway to suppress CPT-induced R-loops (Figure S6D). Next, we tested whether DHX9 and PARP1 are required for their reciprocal recruitment to chromatin in response to CPT. Depletion of PARP1 did not compromise DHX9 recruitment to the *β-actin* gene (Figure S6E). Similarly, PARP1 recruitment was not affected upon DHX9 loss (Figure S6F). These results suggest that DHX9 and PARP1 promote the suppression of CPT-induced R-loops independent of their reciprocal recruitment to chromatin.

### DHX9 Prevents R-Loop-Dependent DNA Damage in Response to CPT

**Figure 6:**
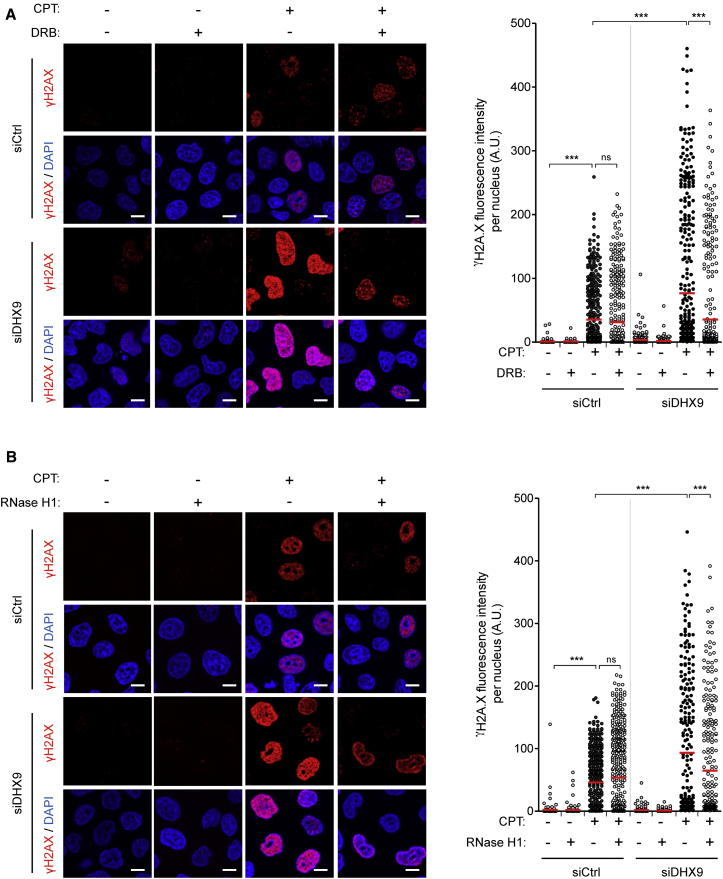
DHX9 Prevents R-Loop-Associated DNA Damage in Response to CPT (A) IF analysis of HeLa cells transfected with control or DHX9 #1 siRNA and treated with DRB before the addition of CPT for 60 min and stained for γH2AX (red) and DAPI (blue). Left: representative images. Bars, 10 μm. Right: γH2AX fluorescence intensity per nucleus from a representative experiment (≥300 nuclei were analyzed per condition). The horizontal red bars represent the means, and each dot is one nucleus. (B) The same as in (A), but instead of DRB, cells were transfected with FLAG (−RNase H1) or RNase H1 plasmid. ^∗∗∗^p < 0.001, ns, not significant (one-way ANOVA Tukey’s multiple comparisons test). See also Figure S7.

An excess of R-loops promotes DNA damage and genome instability (Aguilera and García-Muse, 2012, Skourti-Stathaki and Proudfoot, 2014, Sollier and Cimprich, 2015). Therefore, we examined whether R-loop accumulation in DHX9-depleted cells caused a global increase in R-loop-dependent DNA damage by analyzing γH2AX signal by IF. In line with the increased accumulation of R-loops (Figure 5D), DHX9-depleted cells showed a significant increase in γH2AX signal following CPT treatment (Figure 6A). Inhibition of transcription with 5,6-dichloro-1-β-D-ribofuranosylbenzimidazole (DRB) or cordycepin clearly reduced γH2AX induction, which is consistent with co-transcriptional formation of R-loops (Figures 6A and S7A). Furthermore, overexpression of RNase H1 significantly reduced the CPT-induced γH2AX signal in DHX9-depleted cells (Figure 6B). Co-staining against RNase H1 showed that cells overexpressing RNase H1 exhibited low γH2AX levels, while non-expressing cells showed high γH2AX signal (Figure S7B). Therefore, our data demonstrate that DHX9 prevents R-loop-associated DNA damage in response to CPT.

### R-Loop Helicases and Cancer

**Figure 7:**
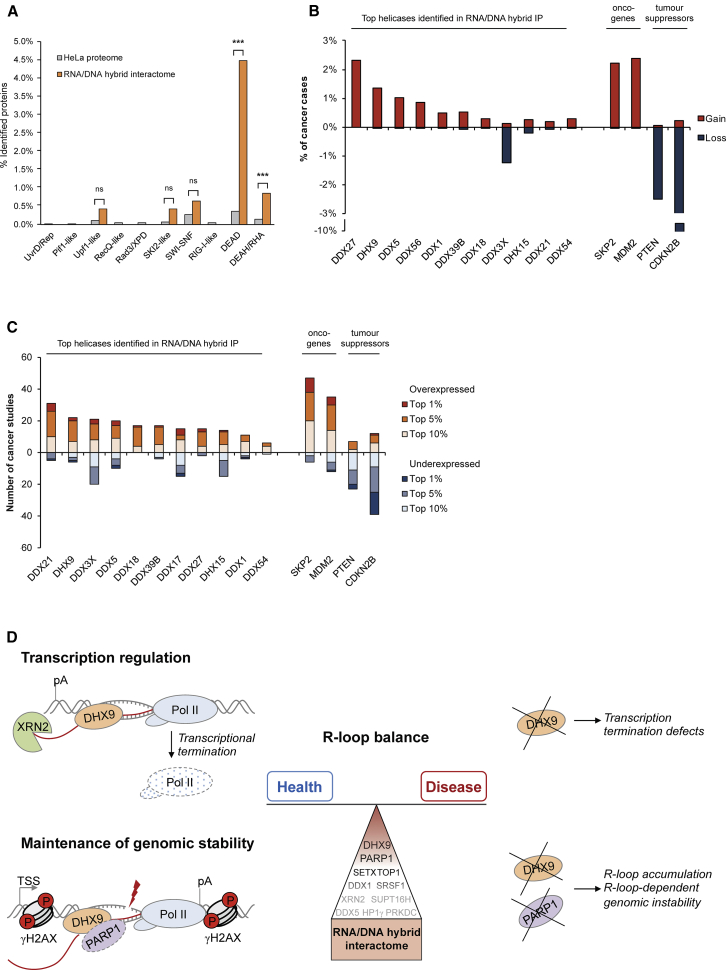
RNA/DNA Hybrid Interactome Helicases Are Amplified in Human Cancers (A) Enrichment of DEAD and DEAH/RHA helicases in the RNA/DNA hybrid interactome. (B) Gain (red) and loss (blue) of RNA/DNA hybrid-interacting DEAD/H helicases in cancer. The y axis shows the percentage of the total tested cancer samples based on copy number variations (COSMIC database). (C) Transcriptional expression changes of RNA/DNA hybrid-interacting DEAD/H helicases in cancer. The y axis shows the number of cancer studies (ONCOMINE database). (D) Model showing the role of the RNA/DNA hybrid interactome and the top candidate DHX9 in regulating R-loop balance in health and disease.

The role of DHX9 in R-loop-associated DNA damage pointed toward its potential involvement in human cancer because one of cancer hallmarks is widespread genomic instability. In addition to DHX9, other DEAD/H helicases were enriched in the RNA/DNA hybrid interactome (Figure 7A), suggesting a general role for these helicases in R-loop processes. In agreement with a possible role for these RNA/DNA hybrid-interacting helicases in cancer, analysis of the COSMIC cancer dataset (Forbes et al., 2015) revealed that these DEAD/H helicases are frequently genetically amplified in cancer, similar to known oncogenes SKP2 and MDM2 (Figure 7B). Moreover, most identified helicases, including DHX9 and DDX5, showed mRNA overexpression in a range of cancers, based on the ONCOMINE database (Rhodes et al., 2007) (Figure 7C). Therefore, R-loop-interacting helicases may play a role in oncogenesis or cancer development.

## Discussion

In this study, we established and validated a specific affinity-based MS approach with the S9.6 antibody to define a comprehensive RNA/DNA hybrid interactome in HeLa cells (Figures 1 and 2). The RNA/DNA hybrid interactome represents a unique functional subset of the total HeLa proteome enriched for dual DNA- and RNA-binding proteins and RNA-processing factors. It comprises several proteins with previously described functions in R-loop biology, such as SRSF1, Top1, and FACT (Aguilera and García-Muse, 2012, Skourti-Stathaki and Proudfoot, 2014). The RNA/DNA hybrid interactome also reveals several new classes of *in vivo* RNA/DNA hybrid-binding proteins, including RNA-processing factors, helicases, histone modifiers, and DNA repair factors (Figure 2; Table S2), suggesting that these processes are linked to R-loops. It should be noted that some expected proteins, including SETX and RNase H1, were not identified by MS. This could be because of limited MS sensitivity for low-abundance proteins or their dynamic association with RNA/DNA hybrids *in vivo*. We experimentally validated a number of new candidates from the RNA/DNA hybrid interactome and demonstrated their possible implication in R-loop biology (Figure 3). Although future work is required to determine the specific function of these proteins in R-loop metabolism, these data highlight the value of the RNA/DNA hybrid interactome in understanding R-loop biology.

In this article, we specifically focused on R-loop-associated function of a top interactome candidate, helicase DHX9. Previous work demonstrated that DHX9 can resolve RNA/DNA hybrids *in vitro* (Chakraborty and Grosse, 2011). We report here that DHX9 interacts with RNA/DNA hybrids *in vivo* and it promotes R-loop suppression and transcriptional termination (Figure 4). Furthermore, DHX9 is important for maintaining genomic stability in response to CPT by preventing R-loop accumulation (Figures 5 and 6). However, it is not clear how DHX9 recognizes and suppresses physiological R-loops at transcription termination regions and CPT-induced R-loops.

DHX9 is involved in various aspects of RNA metabolism (Lee and Pelletier, 2016), and its depletion results in altered transcription (Chen et al., 2014). This broad involvement in transcription underlies the global decrease of R-loop levels detected by slot blot and IF upon DHX9 depletion (Figure 3). The new characterized role of DHX9 in transcriptional termination accounts for the specific R-loop accumulation at the termination regions of *β-actin* and *γ-actin* genes (Figure 4). In this respect, DHX9 behaves similarly to SETX (Skourti-Stathaki et al., 2011). DHX9 can regulate transcription either by binding directly to RNA (Liu et al., 2013) and gene promoters (Myöhänen and Baylin, 2001) or by mechanisms dependent on its nucleoside-triphosphatase (NTPase)/helicase activity (Nakajima et al., 1997) and its ability to coordinate protein-protein interactions (Anderson et al., 1998). Indeed, DHX9 interacts with a large number of proteins that function at the interface of transcription and DNA damage response, including RNA Pol II, CBP, BRCA1 (Anderson et al., 1998, Nakajima et al., 1997), SMN (Pellizzoni et al., 2001), DDX5 (Hegele et al., 2012), topoisomerase IIα (Zhou et al., 2003), and DNA-PK (Mischo et al., 2005). Therefore, DHX9 may be recruited to the promoters of transcribed genes by different mechanisms, and then, by interacting with Pol II, it can travel along the gene suppressing the arising R-loops.

Endogenous DHX9 is found in the same complexes as PARP1, another RNA/DNA hybrid interactor (Figure 5), which is in line with data from MS screens for PARP1 and PARylation interactors (Gagné et al., 2012, Isabelle et al., 2010). The loss of DHX9 and PARP1 independently results in increased R-loop accumulation following CPT treatment. An intriguing possibility is that DHX9 and PARP1 could be in the same pathway to suppress CPT-induced R-loops. PARP1 is not required for DHX9 recruitment to chromatin, but it may regulate DHX9 helicase activity in a similar way as it regulates the activity of the Werner syndrome helicase (von Kobbe et al., 2004). Moreover, our data show that following CPT treatment, PARP1 and DHX9 both are recruited in the body and at the 3′ end of the *β-actin* gene, corresponding to R-loop peaks and the surrounding chromatin. The local increased concentration of R-loop-processing factors around the CPT-induced R-loops could be a way for the cells to deal with pathological R-loops, representing a threat to genome stability. However, PARP1 is known to play key roles in the repair of CPT-induced DNA lesions (Cristini et al., 2016, Das et al., 2014) and in other DNA repair pathways (Tallis et al., 2014). Therefore, following CPT treatment, PARP1 also could be recruited to the *β-actin* gene because of its function in DNA repair, independent of R-loop induction. This may explain the lack of exact co-localization between DHX9, PARP1, and CPT-induced R-loop peaks over the *β-actin* gene.

In addition to a potential model of cooperation between PARP1 and DHX9 in processing R-loops, different non-exclusive mechanisms could exist. First, PARP1 has many interactors that could process R-loops (Isabelle et al., 2010). Second, PARylation itself triggers the recruitment of RNA-processing factors such as FUS, TAF15, EWSF1, and SAFA to DNA damage (Britton et al., 2014). These factors are known R-loop suppressors (Aguilera and García-Muse, 2012, Skourti-Stathaki and Proudfoot, 2014). Finally, R-loop accumulation could be a consequence of impaired DNA damage repair. Indeed, R-loops have been described at DNA breaks (Britton et al., 2014, Cohen et al., 2018). Our data suggest a potential role of PARP1 in controlling R-loop balance, but given the complexity of this scenario, further studies are required to understand the mechanistic details underlying PARP1 function in R-loop metabolism.

Recently, it has been demonstrated that increased global transcription in response to oncogenes or hormones is a characteristic of cancer (Kotsantis et al., 2016, Stork et al., 2016). This transcriptional burst is accompanied by the accumulation of R-loops, which directly contributes to replication stress and genomic instability (Aguilera and García-Muse, 2012, Skourti-Stathaki and Proudfoot, 2014, Sollier and Cimprich, 2015). These findings suggest that an increased R-loop level could be a common feature of cancer cells. We found that multiple members of the DEAD/H helicase family, including DHX9, are strongly enriched in the RNA/DNA hybrid interactome and they are frequently deregulated in a range of cancers (Figure 7). These helicases may be required to support a higher transcriptional and RNA metabolic activity of cancer cells because of their role in transcription, RNA processing, and export. However, DHX9 and other DEAD/H helicases also may be upregulated in cancer to prevent R-loop accumulation or to promote the restart of replication forks stalled by the excess of R-loops. Future work will determine whether these helicases play a role in oncogenesis, tumor progression, or drug resistance by promoting R-loop processing.

In conclusion, our study provides the first proteomic characterization of factors interacting with RNA/DNA hybrids *in vivo*, offering a new perspective on cellular R-loop functions, including transcriptional termination and maintenance of genome stability (Figure 7D). Thus, the RNA/DNA hybrid interactome constitutes a powerful resource to study R-loop biology in health and disease.

## Experimental Procedures

### Cell Culture, Transfections, and Drug Treatments

HeLa and HEK293T cells were grown as described (Skourti-Stathaki et al., 2011). Cells were treated with 5 μg/mL actinomycin D for 6 hr, 10 μM CPT for the indicated time, 100 μM DRB for 3 hr, 50 μM cordycepin for 4 hr, and 10 μM or 0.1 μM Olaparib for 1 hr. Cell transfections are described in Supplemental Experimental Procedures.

### ChIP and DIP Analysis

ChIP with 2–5 μg of the indicated antibodies was carried out as described (Groh et al., 2014). DIP analysis with the S9.6 antibody (Boguslawski et al., 1986) was described by Groh et al. (2014) and Skourti-Stathaki et al. (2011). RNase H digestion with 1.5 U RNase H (M0297, NEB) per microgram genomic DNA for 2.5 hr at 37°C was carried out before IP. The amount of immunoprecipitated material at a particular gene region was calculated as the percentage of input after subtracting the background signal (no antibody control). Where stated, the values were normalized to the indicated probes.

### RNA/DNA Hybrid IP

Non-crosslinked HeLa cells were lysed in 85 mM KCl, 5 mM PIPES (pH 8.0), and 0.5% NP-40 for 10 min on ice. Pelleted nuclei were resuspended in RSB buffer (10 mM Tris-HCl pH 7.5, 200 mM NaCl, 2.5 mM MgCl2) with 0.2% sodium deoxycholate [NaDOC], 0.1% SDS, 0.05% sodium lauroyl sarcosinate [Na sarkosyl] and 0.5% Triton X-100, and extracts were sonicated for 10 min (Diagenode Bioruptor). Extracts were then diluted 1:4 in RSB with 0.5% Triton X-100 (RSB + T) and subjected to IP with the S9.6 antibody, bound to protein A dynabeads (Invitrogen), and preblocked with 0.5% BSA/PBS for 2 hr. CBP80 and IgG2a antibodies were used as control. RNase A (PureLink, Invitrogen) was added during IP at 0.1 ng RNase A per microgram genomic DNA. Beads were washed 4x with RSB + T; 2x with RSB; and eluted either in 2x LDS (Invitrogen), 100 mM DTT for 10 min at 70°C (for SDS-PAGE), or 1% SDS and 0.1 M NaHCO_3_ for 30 min at room temperature (for RNA/DNA hybrid slot blot). Where indicated, nuclear extracts were treated with 1 U/μL benzonase (Sigma) for 30 min at 37°C before IP. Sequences and preparation of double-stranded competitors were described by Phillips et al. (2013) and Rigby et al. (2014). For MS analysis, eluted samples were processed by filter-aided sample preparation (FASP) with trypsin (Wiśniewski et al., 2009). Table S1 provides the list of proteins that make up the RNA/DNA interactome.

### Statistical Analysis

Unless otherwise stated, values represent the means ± SEMs based on at least three independent experiments. Asterisks indicate statistical significance (^∗^p < 0.05; ^∗∗^p < 0.01; ^∗∗∗^p < 0.001), based on unpaired, two-tailed Student’s t test. Correlations were calculated using Pearson’s correlation coefficient (*r*).

## Data and Software Availability

The accession number for the RNA/DNA interactome reported in this paper is PRIDE: PXD002960 (http://www.ebi.ac.uk/pride/archive/login).

## Supplemental Information

Document S1. Supplemental Experimental Procedures, Figures S1–S7, and Tables S2 and S3

Table S1. Full Protein List of RNA/DNA Hybrid Interactome, Related to Figure 2 List of proteins identified by MS that make up the RNA/DNA interactome.

Document S2. Article plus Supplemental Information
