# Supplementary material for: RNA/DNA Hybrid Interactome Identifies DXH9 as a Molecular Player in Transcriptional Termination and R-Loop-Associated DNA Damage
Source: Cell Rep. 2018 May 8;23(6):1891–905. doi: 10.1016/j.celrep.2018.04.025 (PMC5976580; doi:10.1016/j.celrep.2018.04.025)
Supplement: Document S1. Supplemental Experimental Procedures, Figures S1–S7, and Tables S2 and S3 [file mmc1.pdf]

Cell Reports, Volume *23*

Supplemental Information

RNA/DNA Hybrid Interactome Identifies DXH9 as a Molecular Player in Transcriptional Termination and R-Loop-Associated DNA Damage

Agnese Cristini, Matthias Groh, Maiken S. Kristiansen, and Natalia Gromak

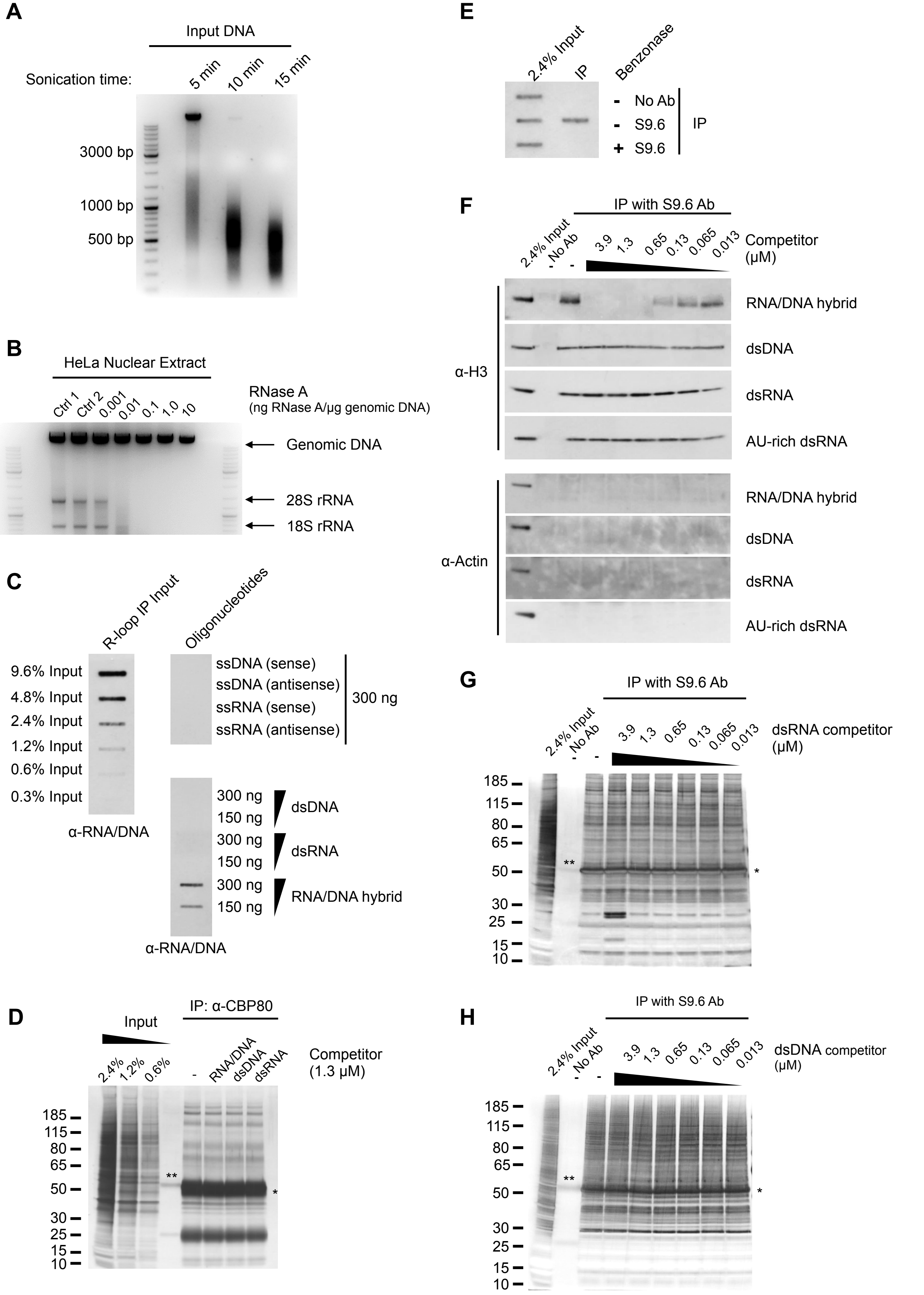
A
Input DNA
Sonication time:
5 min
10 min
15 min
3000 bp
1000 bp
500 bp
B
HeLa Nuclear Extract
Ctrl 1
Ctrl 2
0.001
0.01
0.1
1.0
10
RNase A
(ng RNase A/µg genomic DNA)
Genomic DNA
28S rRNA
18S rRNA
C
R-loop IP Input
Oligonucleotides
9.6% Input
4.8% Input
2.4% Input
1.2% Input
0.6% Input
0.3% Input
α-RNA/DNA
ssDNA (sense)
ssDNA (antisense)
ssRNA (sense)
ssRNA (antisense)
300 ng
300 ng
150 ng
dsDNA
dsRNA
RNA/DNA hybrid
α-RNA/DNA
D
Input
IP: α-CBP80
2.4%
1.2%
0.6%
-
RNA/DNA
dsDNA
dsRNA
Competitor
(1.3 µM)
185
115
80
65
50
30
25
15
10
**
*
E
2.4% Input
IP
Benzonase
- No Ab
- S9.6
+ S9.6
IP
F
IP with S9.6 Ab
2.4% Input
No Ab
3.9
1.3
0.65
0.13
0.065
0.013
Competitor
(µM)
α-H3
RNA/DNA hybrid
dsDNA
dsRNA
AU-rich dsRNA
α-Actin
RNA/DNA hybrid
dsDNA
dsRNA
AU-rich dsRNA
G
IP with S9.6 Ab
2.4% Input
No Ab
3.9
1.3
0.65
0.13
0.065
0.013
dsRNA competitor
(µM)
185
115
80
65
50
30
25
15
10
**
*
H
IP with S9.6 Ab
2.4% Input
No Ab
3.9
1.3
0.65
0.13
0.065
0.013
dsDNA competitor
(µM)
185
115
80
65
50
30
25
15
10
**
*

Figure S1. Design and validation of RNA/DNA hybrid IP method. Related to Figure 1

A. Agarose gel analysis of DNA fragment sizes from RNA/DNA hybrid IP samples, sonicated for 5, 10 and 15 min. DNA size markers are shown to the left of the gel.

B. Agarose gel analysis of RNA trimming by RNase A during RNA/DNA hybrid IP. 5 µg of purified genomic DNA, prepared according to the RNA/DNA hybrid IP protocol, was treated with RNase A for 2h at 37°C. Control samples (Ctrl 1 and Ctrl 2) were not treated with RNase A. The migration of genomic DNA and ribosomal RNA (18S and 28S rRNA) is indicated on the right of the gel.

C. S9.6 antibody specifically recognises endogenous and synthetic RNA/DNA hybrids at a wide range of concentrations. Slot blot analysis with S9.6 antibody. Different amounts of endogenous and synthetic RNA/DNA hybrids were loaded on the slot blot. 300 ng of indicated synthetic single-stranded oligonucleotides were used as negative controls (right top panel).

D. Silver-stain of CBP80 IP with nucleic acid competitors added at 1.3 uM.

E. RNA/DNA hybrid slot blot after benzonase treatment.

F. Western blot of RNA/DNA hybrid IP samples in the presence of indicated competitors added at 3.9-0.013 uM. Western blot was probed with histone H3 (top panel) and actin (bottom panel) antibodies.

G-H. Silver-stain of RNA/DNA hybrid IP with dsRNA (G) and dsDNA (H) competitors.

Asterisk (*) indicates the heavy chain from the S9.6 and IgG2a antibodies. Band labelled (**) in ‘No Ab’ lane corresponds to BSA, used to block protein A Dynabeads.

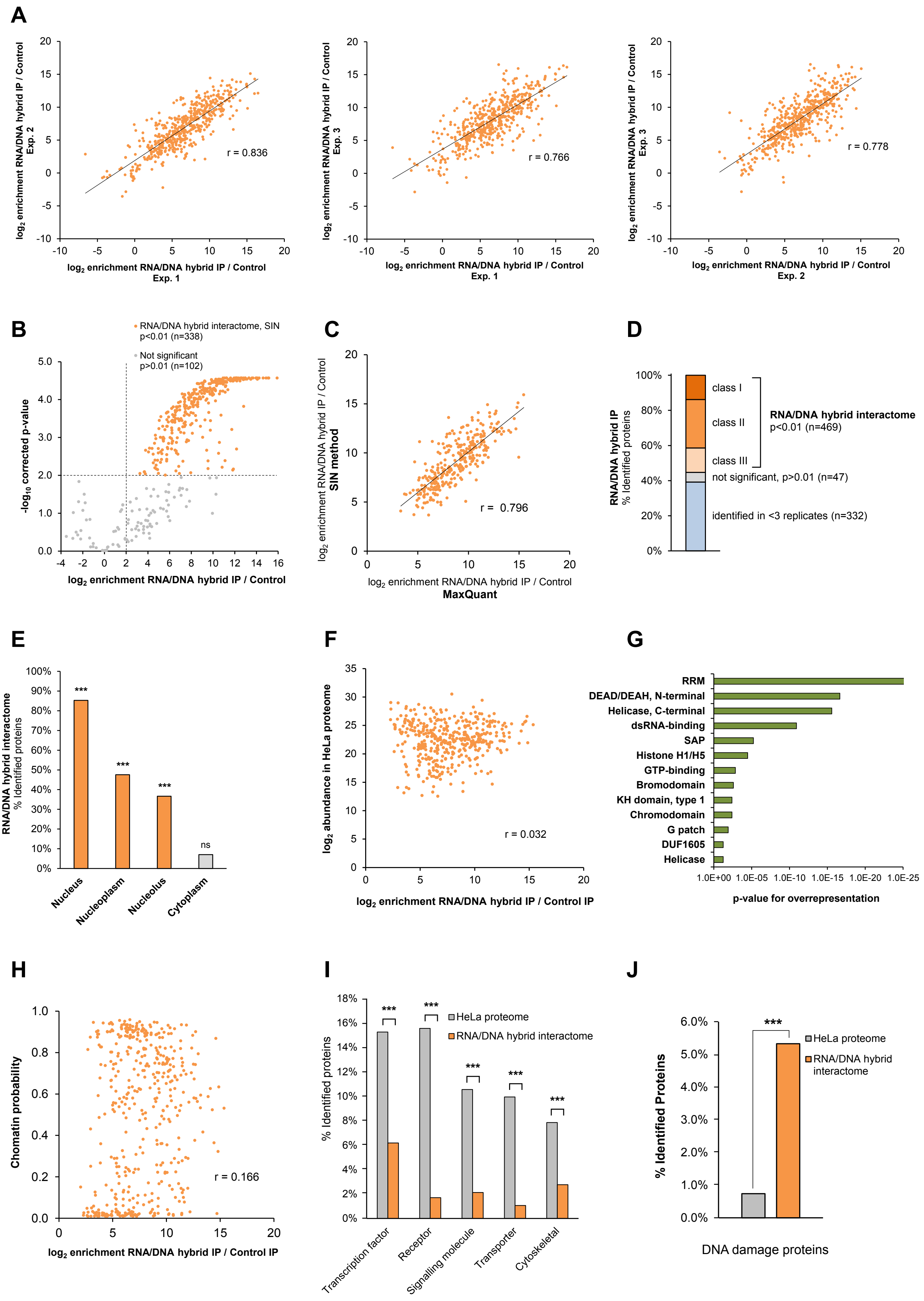
A
log2 enrichment RNA/DNA hybrid IP / Control Exp. 2
log2 enrichment RNA/DNA hybrid IP / Control Exp. 1
r = 0.836
log2 enrichment RNA/DNA hybrid IP / Control Exp. 3
log2 enrichment RNA/DNA hybrid IP / Control Exp. 1
r = 0.766
log2 enrichment RNA/DNA hybrid IP / Control Exp. 3
log2 enrichment RNA/DNA hybrid IP / Control Exp. 2
r = 0.778
B
RNA/DNA hybrid interactome, SIN p<0.01 (n=338)
Not significant p>0.01 (n=102)
-log10 corrected p-value
log2 enrichment RNA/DNA hybrid IP / Control
C
log2 enrichment RNA/DNA hybrid IP / Control SIN method
log2 enrichment RNA/DNA hybrid IP / Control MaxQuant
r = 0.796
D
RNA/DNA hybrid IP % Identified proteins
class I
class II
class III
RNA/DNA hybrid interactome p<0.01 (n=469)
not significant, p>0.01 (n=47)
identified in <3 replicates (n=332)
E
RNA/DNA hybrid interactome % Identified proteins
***
***
***
ns
Nucleus
Nucleoplasm
Nucleolus
Cytoplasm
F
log2 abundance in HeLa proteome
log2 enrichment RNA/DNA hybrid IP / Control IP
r = 0.032
G
RRM
DEAD/DEAH, N-terminal
Helicase, C-terminal
dsRNA-binding
SAP
Histone H1/H5
GTP-binding
Bromodomain
KH domain, type 1
Chromodomain
G patch
DUF1605
Helicase
1.0E+00 1.0E-05 1.0E-10 1.0E-15 1.0E-20 1.0E-25
p-value for overrepresentation
H
Chomatin probability
log2 enrichment RNA/DNA hybrid IP / Control IP
r = 0.166
I
% Identified proteins
HeLa proteome
RNA/DNA hybrid interactome
***
Transcription factor
Receptor
Signalling molecule
Transporter
Cytoskeletal
J
% Identified Proteins
***
HeLa proteome
RNA/DNA hybrid interactome
DNA damage proteins

Figure S2. **RNA/DNA hybrid interactome analysis. Related to Figure 2**

A. RNA/DNA hybrid IP is highly reproducible. The panel shows the correlation between log_2 enrichment RNA/DNA hybrid IP / Control IP of proteins quantified in three independent biological replicates of RNA/DNA hybrid IP by mass spectrometry.

B. Volcano plot displaying mass spectrometry results of three biological replicates of RNA/DNA hybrid IP experiments, using normalised spectral indexes (SIN) quantification method, as implemented in the SINQ software (Trudgian et al., 2011). Averaged log_2 ratios between RNA/DNA hybrid IP and control IP, carried out in the presence of 1.3 uM synthetic RNA/DNA hybrid, are plotted against their Benjamini-Hochberg corrected $-log_{10}$ p-values calculated across all three biological replicates using a moderated t-test. Proteins (n=338) significantly enriched in R-loop IP/Control are plotted in orange. Dashed lines indicate the significance cutoffs (log_2 enrichment > 2 and $–log_{10} > 2$).

C. Correlation between protein enrichment in RNA/DNA hybrid IP/Control IP of proteins quantified by MaxQuant method and SIN method.

D. Classification of proteins identified in RNA/DNA hybrid IP mass spectrometry on the basis on moderated t-test of three biological replicates with p-value corrected according to Benjamini-Hochberg. 469 Proteins enriched in RNA/DNA hybrid IP (corrected p-value <0.01) represented the 'RNA/DNA hybrid interactome'. The RNA/DNA hybrid interactome was further subdivided into three classes according to the corrected p-values: top 25% (class I), middle 50% (class II), and bottom 25% (class III). 379 proteins were identified but not enriched (grey).

E. Cellular compartment analysis of RNA/DNA hybrid interactome ($p < 0.01$). Asterisks (***) indicate highly significant enrichment of the depicted compartments in RNA/DNA hybrid IP as determined by Fisher's exact test (Benjamini-Hochberg corrected p-values of $2x10^{-127}$, $1x10^{-69}$, $8x10^{-113}$, respectively).

F. Relative protein abundance in RNA/DNA hybrid interactome as compared to the total cellular protein abundance in HeLa cells (Geiger et al., 2012). Log_2 enrichment RNA/DNA hybrid IP/Control IP of proteins in RNA/DNA hybrid interactome (x-axis) is plotted against their corresponding Log_2 abundance in the total HeLa proteome (y-axis).

G. Protein domains overrepresented in RNA/DNA hybrid IP. Analysis of enrichment of Pfam InterPro Domains in RNA/DNA hybrid interactome using 'Enrichr' software (Chen et al., 2013). Top thirteen significantly overrepresented protein domains as determined by Fisher's exact test are shown and ranked according to their Benjamini-Hochberg corrected p-value.

H. Relative protein abundance in RNA/DNA hybrid interactome compared to chromatin probability (Kustatscher et al., 2014). Log_2 enrichment RNA/DNA hybrid IP/Control IP of proteins in RNA/DNA hybrid interactome (x-axis) is plotted against their corresponding chromatin probability (y-axis).

I-J. Enrichment analysis of abundant protein families (I) and factors mediating genome stability (Paulsen et al., 2009) (J) in the RNA/DNA hybrid interactome.

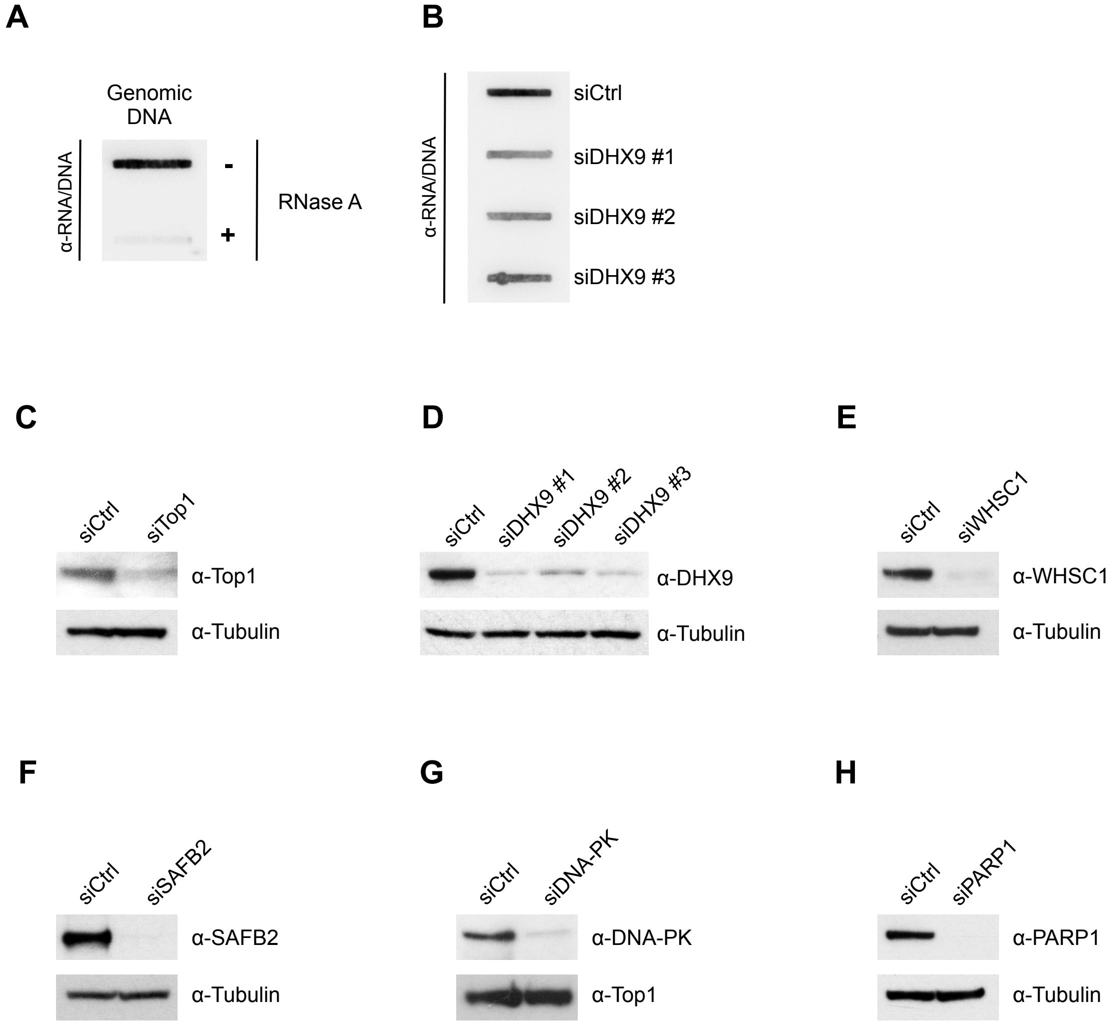

Figure S3. Validation of new RNA/DNA hybrid interactome candidates. Related to Figure 3

A. RNA/DNA hybrid slot blot of HeLa genomic DNA from nuclear extracts treated with 0.1 mg/ml RNase A for 1 h at 37°C and probed with S9.6 antibody.

B. RNA/DNA hybrid slot blot of genomic DNA from HeLa cells transfected with control (siCtrl) or the indicated siRNA targeting DHX9.

C-H. Western blot of whole cell extracts from HeLa cells transfected with control (siCtrl) or indicated siRNAs sequences. Blots were probed with the indicated antibodies. Tubulin and Top1 were used as loading controls.

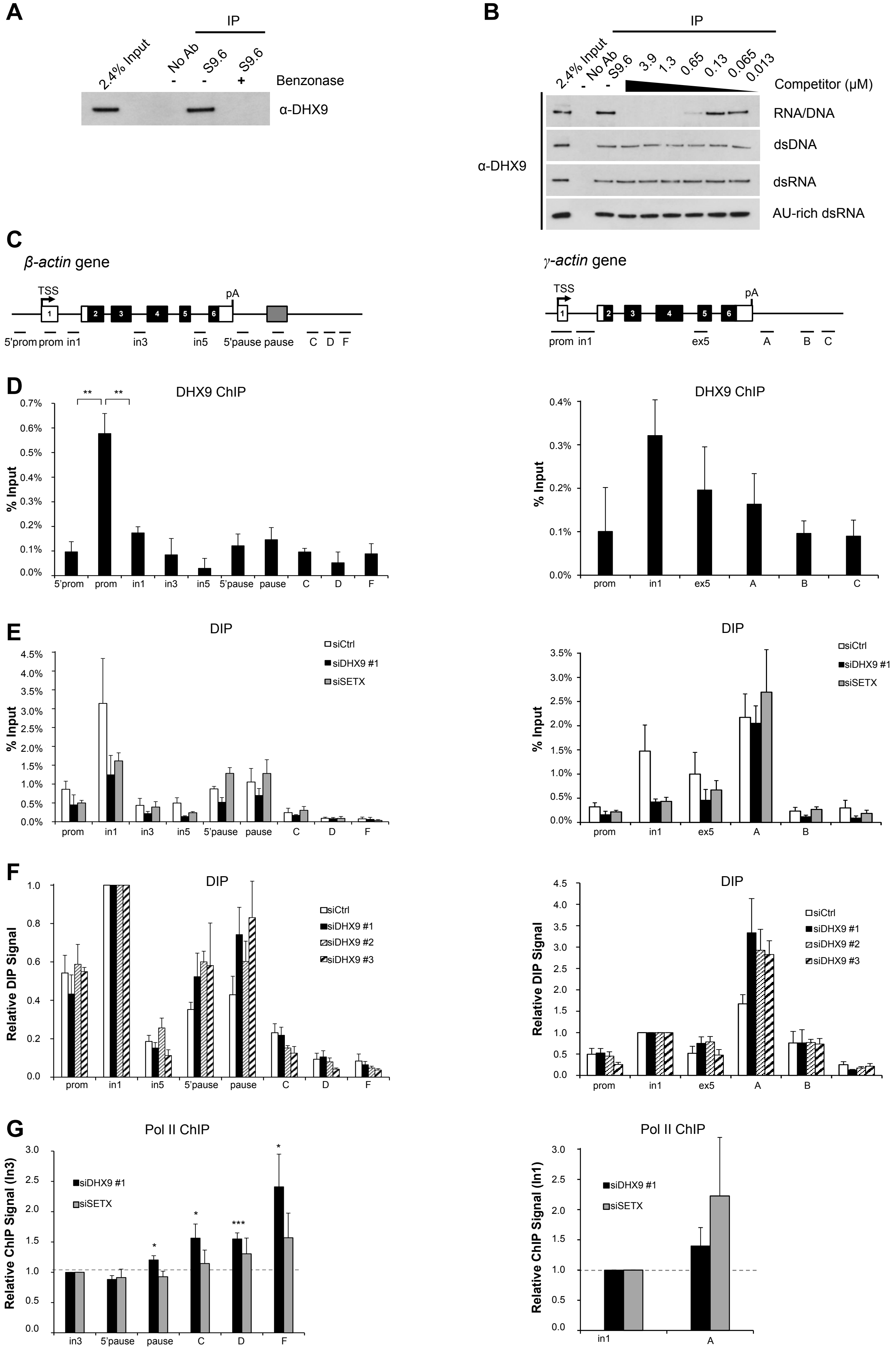
A
IP
2.4% Input
No Ab
S9.6
S9.6
- - +
Benzonase
α-DHX9
B
IP
2.4% Input
No Ab
S9.6
3.9
1.3
0.65
0.13
0.065
0.013
Competitor (μM)
α-DHX9
RNA/DNA
dsDNA
dsRNA
AU-rich dsRNA
C
β-actin gene
TSS
pA
5'prom prom in1 in3 in5 5'pause pause C D F
γ-actin gene
TSS
pA
prom in1 ex5 A B C
D
DHX9 ChIP
% Input
5'prom prom in1 in3 in5 5'pause pause C D F
DHX9 ChIP
% Input
prom in1 ex5 A B C
E
DIP
% Input
siCtrl
siDHX9 #1
siSETX
prom in1 in3 in5 5'pause pause C D F
DIP
% Input
prom in1 ex5 A B
F
DIP
Relative DIP Signal
siCtrl
siDHX9 #1
siDHX9 #2
siDHX9 #3
prom in1 in5 5'pause pause C D F
DIP
Relative DIP Signal
prom in1 ex5 A B
G
Pol II ChIP
Relative ChIP Signal (In3)
siDHX9 #1
siSETX
in3 5'pause pause C D F
Pol II ChIP
Relative ChIP Signal (In1)
in1 A

Figure S4. DHX9 binds R-loops *in vivo* and promotes transcription termination. Related to Figure 4

A. Western blot of RNA/DNA hybrids IP samples. Nuclear extracts were treated with benzonase (1 U/ul) for 30 min prior to IP with S9.6 antibody. Western blot was probed with DHX9 antibody.

B. Western blot of DHX9 in RNA/DNA hybrid IP in presence of the indicated synthetic competitors.

C. Diagram of *β-actin* (left panel) and *γ-actin* (right panel) genes. Exons are black, UTRs are white, TSS is transcriptional start site. qPCR amplicons are shown below the diagram. Grey box denotes a termination region.

D. DHX9 ChIP in HeLa cells on *β-actin* (left panel) and *γ-actin* (right panel) genes. Values are % of Input.

E. DIP in HeLa cells, transfected with control (siCtrl), DHX9 #1 and SETX siRNAs, on *β-actin* (left panel) and *γ-actin* (right panel) genes. Values are percentage of input from Figure 4H.

F. DIP in HeLa cells, transfected with the indicated siRNAs targeting DHX9 or with a control sequence (siCtrl), on *β-actin* (left panel) and *γ-actin* (right panel) genes. Values are relative to in1.

G. Pol II ChIP in HeLa cells, transfected with control (siCtrl), DHX9 #1 and SETX siRNAs, on *β-actin* (left panel) and *γ-actin* (right panel) genes. Values are normalized to *β-actin* in 3 and *γ-actin* in 1, respectively.

Bars in D-G represent the average values from at least three independent experiments +/- SEM with * $p<0.05$, ** $p<0.01$, *** $p<0.001$ (unpaired, two-tailed Student's t test).

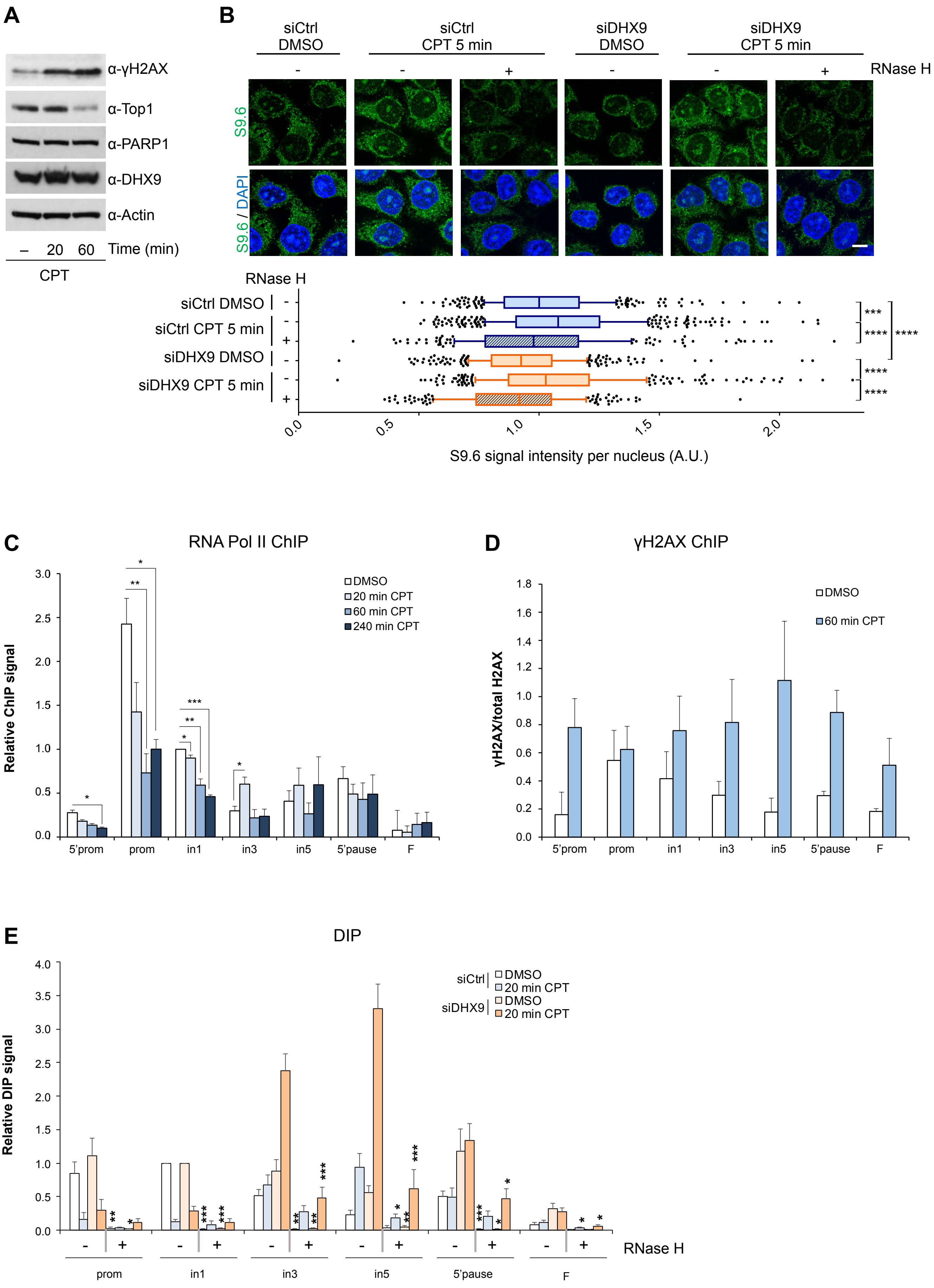
A
α-γH2AX
α-Top1
α-PARP1
α-DHX9
α-Actin
– 20 60 Time (min)
CPT
B
siCtrl DMSO
siCtrl CPT 5 min
siDHX9 DMSO
siDHX9 CPT 5 min
- - + - - + RNase H
S9.6
S9.6 / DAPI
RNase H
siCtrl DMSO
siCtrl CPT 5 min
siDHX9 DMSO
siDHX9 CPT 5 min
***
****
****
****
****
0.0 0.5 1.0 1.5 2.0
S9.6 signal intensity per nucleus (A.U.)
C
RNA Pol II ChIP
Relative ChIP signal
DMSO
20 min CPT
60 min CPT
240 min CPT
5'prom prom in1 in3 in5 5'pause F
D
γH2AX ChIP
γH2AX/total H2AX
DMSO
60 min CPT
5'prom prom in1 in3 in5 5'pause F
E
DIP
Relative DIP signal
siCtrl DMSO
20 min CPT
siDHX9 DMSO
20 min CPT
RNase H
prom in1 in3 in5 5'pause F

Figure S5. DNA damage and R-loop induction upon CPT treatment. Related to Figure 5

A. Western blot of total HeLa extracts, treated with DMSO or CPT for the indicated times, probed with indicated antibodies. Actin was used as loading control.

B. IF analysis of R-loops using S9.6 antibody (green) in Hela cells transfected with DHX9 #1 or control siRNA and treated with DMSO or CPT for 5 min. DAPI (blue) depicts the nuclei. Cells were treated (+) or left untreated (-) with RNase H prior to S9.6 staining. Top panel: representative images. Bar: 10 um. Bottom panel: S9.6 intensity per nucleus. More than 100 nuclei were analyzed per condition (n=3, apart from RNase H conditions that are n=2). The box represents the 25-75 percentile range with the median plotted as horizontal bar; the whiskers are set to 10-90 percentile range. Dots outside the whiskers represent nuclei that are not in the 10-90 percentile range. *** $p<0.001$, ****, $p<0.0001$ (One-way ANOVA test).

C. Pol II ChIP in HeLa cells, treated with DMSO or CPT for the indicated times, on *β-actin* gene. Values are relative to in1 amplicon in DMSO-treated samples.

D. γH2AX ChIP in HeLa cells, treated with DMSO or CPT for 60 min, on *β-actin* gene. γH2AX values are normalized to total H2AX signal for each amplicon.

E. DIP in HeLa cells, transfected with control (shades of blue) or DHX9 #1 (shades of red) siRNA and treated with DMSO or CPT for 20 min, on *β-actin* gene. HeLa genomic DNA was untreated (-) or treated (+) with RNase H prior to IP with S9.6 antibody. Values are relative to in1 for each siRNA. The p-value is calculated for each condition for + RNase H samples versus the - RNase H samples.

Bars in C-E represent the average values from at least three independent experiments +/- SEM with
* $p<0.05$, ** $p<0.01$, *** $p<0.001$ (unpaired, two-tailed Student's t test).

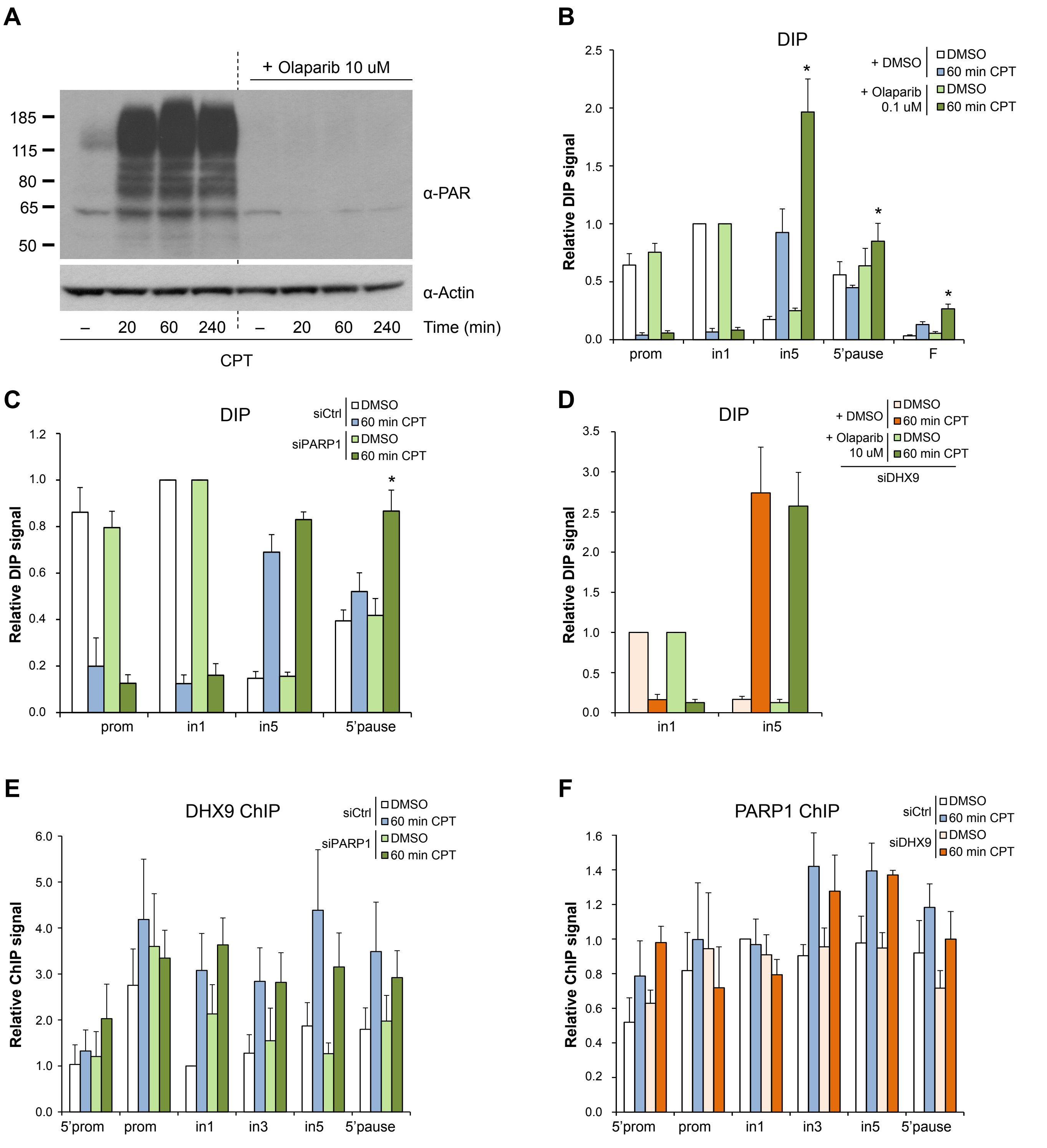

A
+ Olaparib 10 uM
185
115
80
65
50
α-PAR
α-Actin
– 20 60 240 – 20 60 240
Time (min)
CPT
B
DIP
+ DMSO
DMSO
60 min CPT
+ Olaparib 0.1 uM
DMSO
60 min CPT
Relative DIP signal
prom
in1
in5
5'pause
F
C
DIP
siCtrl
siPARP1
DMSO
60 min CPT
Relative DIP signal
D
DIP
+ DMSO
+ Olaparib 10 uM
siDHX9
E
DHX9 ChIP
Relative ChIP signal
5'prom
in3
F
PARP1 ChIP
siDHX9

Figure S6. Effects of PARylation inhibition and PARP1 depletion on R-loops and DHX9. Related to Figure 5

A. Western blot of whole cells extracts from HeLa cells treated with DMSO or 10 uM Olaparib before the addition of DMSO or CPT for the indicated times. Blots were probed with anti-PAR antibody. Actin was used as loading control.

B. DIP analysis in HeLa cells, treated with DMSO (shades of blue) or 0.1 uM Olaparib (shades of green) before the addition of DMSO or CPT for 60 min, on *β-actin* gene. Values are relative to in1 for DMSO and Olaparib. The p-value is calculated for each amplicon for the Olaparib + CPT samples versus the DMSO + CPT samples.

C. DIP analysis in HeLa cells, transfected with control (siCtrl, shades of blue) and PARP1 siRNAs (shades of green) and treated with DMSO or CPT for 60 min, on *β-actin* gene. Values are relative to in1 for each siRNA. The p-value is calculated for the siPARP1 versus the siCtrl sample.

D. DIP analysis in HeLa cells, transfected with siRNA targeting DHX9 (DHX9 #1) and treated with DMSO or 10 uM Olaparib before the addition of DMSO or CPT for 60 min, on *β-actin* gene. Values are relative to in1 for DMSO and Olaparib.

E. DHX9 ChIP in HeLa cells, transfected with control (siCtrl, shades of blue) and PARP1 siRNAs (shades of green) and treated with DMSO or CPT for 60 min, on *β-actin* gene. Values are relative to in1 in siCtrl DMSO-treated samples.

F. PARP1 ChIP in HeLa cells (using anti-PARP1 from Proteintech #22999-1-AP), transfected with control (siCtrl, shades of blue) and DHX9 #1 siRNAs (shades of red) and treated with DMSO or CPT for 60 min, on *β-actin* gene. Values are relative to in1 in siCtrl DMSO-treated samples.

Bars in B-F represent the average values from at least three independent experiments +/- SEM with * $p<0.05$ (unpaired, two-tailed Student's t test).

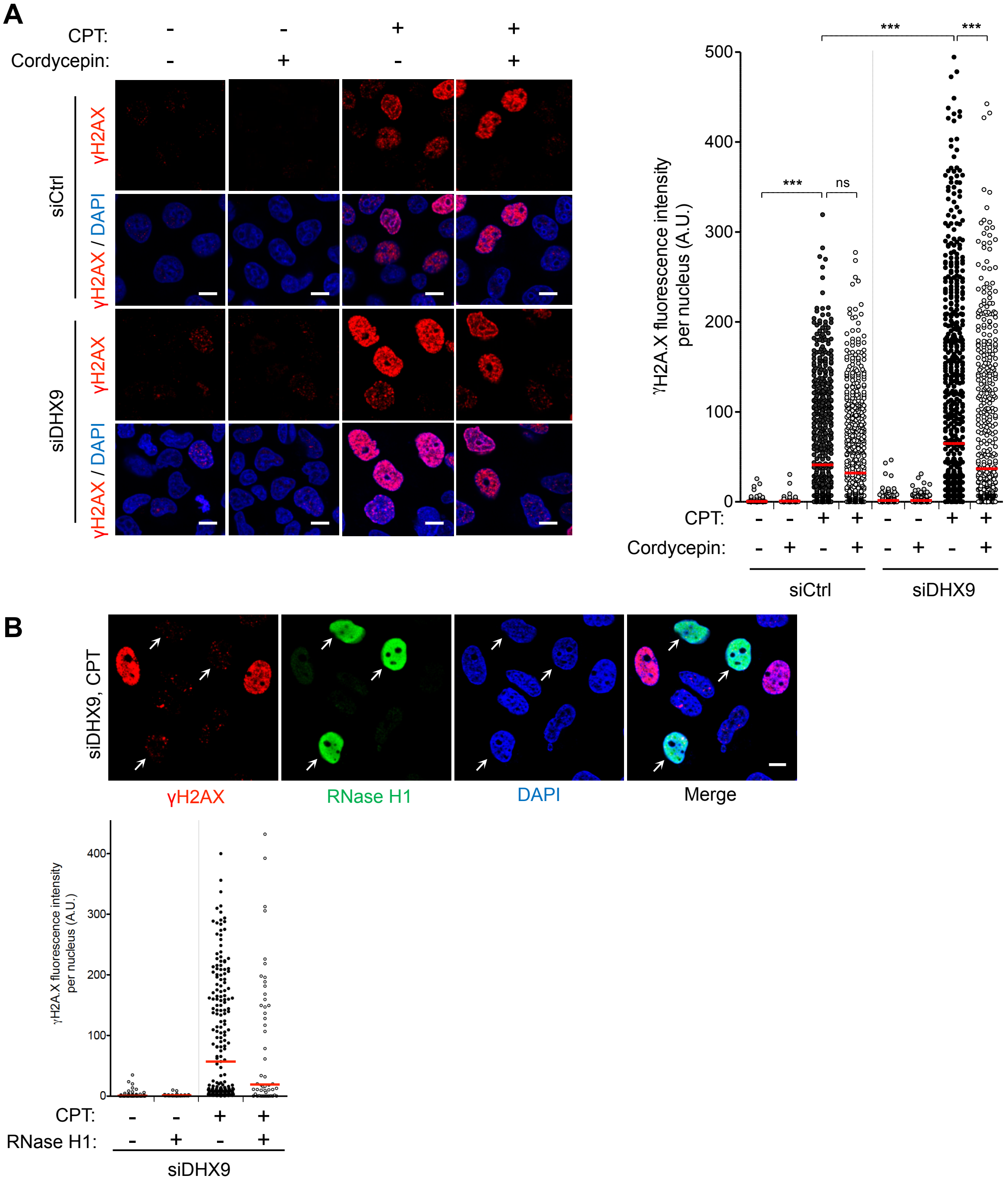

Figure S7. CPT-induced DNA damage in DHX9-depleted cells is transcription-dependent and decreases with RNase H1 overexpression. Related to Figure 6

A. IF analysis of HeLa cells transfected with DHX9 #1 or control siRNA and treated with Cordycepin before the addition of DMSO or CPT for 60 min and stained for γH2AX (red) and DAPI (blue). Left panel: representative images. Bars: 10 um. Right panel: γH2AX fluorescence intensity per nucleus from a representative experiment (≥ 300 nuclei were analyzed per condition). The horizontal red bar represents the means and each dot one nucleus.

B. IF analysis of HeLa cells transfected with DHX9 #1 siRNA and treated with DMSO or CPT for 60 min and then co-stained for γH2AX (red), RNase H1 (green) and DAPI (blue). Arrows indicate RNase H1 transfected cells. Top panel: representative images. Bars: 10 um. Bottom panel: γH2AX fluorescence intensity per nucleus from one representative experiment. Fluorescence was calculated for the cells with (+) or without (-) RNase H1 overexpression (green staining) in each condition. The horizontal red bar represents the median value of fluorescence and each dot corresponds to one nucleus.

Table S1: Full protein list of RNA/DNA hybrid interactome. Related to Figure 2

Table S2. Representative RNA/DNA hybrid interactors identified by MS. Related to Figure 2

Gene	Protein name	Enrichment	p-value	Class
**Transcription**				
DDX5	Probable ATP-dependent RNA helicase DDX5	10.34	1.32E-06	I
ZNF326	DBIRD complex subunit ZNF326	10.09	2.83E-06	I
CTCF	Transcriptional repressor CTCF	8.58	2.88E-06	I
MED19	Mediator of RNA polymerase II transcription subunit 19	6.35	8.20E-06	II
TTF1	Transcription termination factor 1	8.33	8.67E-06	II
**Splicing and Processing**				
SYNCRIP	Heterogeneous nuclear ribonucleoprotein Q	11.69	1.91E-06	I
SNRPE	Small nuclear ribonucleoprotein E	8.77	3.04E-06	I
PRPF19	Pre-mRNA-processing factor 19	10.89	3.45E-06	I
HNRNPA1	Heterogeneous nuclear ribonucleoprotein A1	7.50	4.03E-06	II
TRA2A	Transformer-2 protein homolog alpha	9.88	4.97E-06	II
SRPK1	SRSF protein kinase 1	7.07	1.10E-05	II
U2AF1	Splicing factor U2AF 65 kDa subunit	6.33	1.38E-05	II
SRSF9	Serine/arginine-rich splicing factor 9	7.51	2.60E-05	II
SNRNP70	U1 small nuclear ribonucleoprotein 70 kDa	9.47	1.66E-04	II
U2AF2	Splicing factor U2AF 65 kDa subunit	4.89	2.38E-04	III
**Epigenetic gene regulation**				
WHSC1	Histone-lysine N-methyltransferase NSD2	9.78	2.42E-06	I
HP1BP3	Heterochromatin protein 1-binding protein 3	10.27	3.04E-06	I
HDAC2	Histone deacetylase 2	8.09	5.17E-06	II
BAZ1B	Tyrosine-protein kinase BAZ1B	8.97	5.97E-06	II
MBD2	Methyl-CpG-binding domain protein 2	7.33	1.77E-05	II
NAT10	N-acetyltransferase 10	9.15	3.42E-05	II
KMT2A	Histone-lysine N-methyltransferase 2A	6.17	3.82E-05	II
CDYL	Chromodomain Y-like protein	5.63	3.87E-05	II
BRD7	Bromodomain-containing protein 7	6.73	6.04E-05	II
CBX3	Chromobox protein homolog 3	7.80	8.06E-05	II
RUVBL2	RuvB-like 2	5.40	1.58E-04	II
DNMT1	DNA (cytosine-5)-methyltransferase 1	4.99	1.71E-04	II
SUV39H1	Histone-lysine N-methyltransferase SUV39H1	4.01	1.25E-03	III
CBX5	Chromobox protein homolog 5	2.80	1.56E-03	III
SMARCA5	SWI/SNF-related matrix-associated actin-dependent regulator of chromatin subfamily A member 5	5.75	4.41E-03	III
**DNA replication and repair**				
TOP2A	DNA topoisomerase 2-alpha	7.82	7.76E-06	II

PRKDC	DNA-dependent protein kinase catalytic subunit	7.30	1.94E-05	II
PARP1	Poly [ADP-ribose] polymerase 1	6.47	3.42E-05	II
PARP2	Poly [ADP-ribose] polymerase 2	5.92	9.60E-05	II
PCNA	Proliferating cell nuclear antigen	4.37	1.42E-04	II
DDB1	DNA damage-binding protein 1	5.75	2.87E-04	III
XAB2	XPA Binding Protein 2	5.13	3.71E-04	III
MCM3	DNA replication licensing factor MCM3	3.31	2.81E-03	III

Table S3. RNA/DNA hybrid interactors identified by MS with known implication in R-loop biology in mammalian cells. Related to Figure 2

Gene	Protein name	Enrichment	p-value	Class	Notes	Reference
**Transcription**						
DHX9	ATP-dependent RNA helicase A	12.85	1.11E-06	I	*In vitro*	(Chakraborty and Grosse, 2011)
ILF3	Interleukin enhancer-binding factor 3	11.59	1.82E-06	I		(Nadel et al., 2015)
ILF2	Interleukin enhancer-binding factor 2	11.78	2.27E-06	I		(Nadel et al., 2015)
XRN2	5-3 exoribonuclease 2	9.82	4.10E-06	II		(Morales et al., 2016)
DDX1	ATP-dependent RNA helicase DDX1	8.29	4.94E-06	II		(Li et al., 2016; Li et al., 2008)
SUPT16H	FACT complex subunit SPT16	6.23	2.12E-04	II		(Herrera-Moyano et al., 2014)
SNW1	SNW domain-containing protein 1	8.26	2.39E-04	III		(Paulsen et al., 2009)
SSRP1	FACT complex subunit SSRP1	4.23	1.86E-03	III		(Herrera-Moyano et al., 2014)
**RNA processing and export**						
DDX21	Nucleolar RNA helicase	14.98	7.91E-07	I		(Song et al., 2017)
HNRNPC	Heterogeneous nuclear ribonucleoproteins C1/C2	10.56	1.29E-06	I		(Nadel et al., 2015)
SNRPD1	Small nuclear ribonucleoprotein Sm D1	10.95	1.32E-06	I		(Paulsen et al., 2009)
SNRPB	Small nuclear ribonucleoprotein-associated proteins B	9.33	1.87E-06	I		(Paulsen et al., 2009)
HNRNPU	Heterogeneous nuclear ribonucleoprotein U	14.91	3.62E-06	II		(Britton et al., 2014)

SNRPD3	Small nuclear ribonucleoprotein Sm D3	8.42	3.88E-06	II		(Paulsen et al., 2009)
SNRPA1	U2 small nuclear ribonucleoprotein A	8.51	4.03E-06	II		(Paulsen et al., 2009)
SNRNP40	U5 small nuclear ribonucleoprotein 40 kDa protein	7.91	8.88E-06	II		(Tresini et al., 2015)
FUS	RNA-binding protein FUS	7.94	1.23E-05	II		(Hill et al., 2016; Wang et al., 2015)
TARDBP	TAR DNA-binding protein 43	8.17	1.85E-05	II		(Hill et al., 2016)
PRPF8	Pre-mRNA-processing-splicing factor 8	10.44	2.10E-05	II		(Tresini et al., 2015)
DDX23	Probable ATP-dependent RNA helicase DDX23	6.97	2.11E-05	II		(Sridhara et al., 2017)
TARBP2	RISC-loading complex subunit TARBP2	5.69	3.24E-05	II	*In vitro*	(Vukovic et al., 2014)
TAF15	TATA-binding protein-associated factor 2N	5.10	8.7E-05	II	Direct function in R-loop biology is not determined	(Britton et al., 2014)
CRNKL1	Crooked neck-like protein 1	5.63	1.52E-04	II		(Paulsen et al., 2009)
CDC40	Pre-mRNA-processing factor 17	6.70	2.08E-04	II		(Paulsen et al., 2009)
SRPK2	SRSF protein kinase 2	6.08	2.58E-04	III		(Sridhara et al., 2017)
SRSF3	Serine/arginine-rich splicing factor 3	3.99	3.49E-04	III	*In vitro*	(Li and Manley, 2005)
SRSF2	Serine/arginine-rich splicing factor 2	3.68	5.58E-04	III		(Chen et al., 2018; Li and Manley, 2005)
SRSF1	Serine/arginine-rich splicing factor 1	3.03	9.84E-04	III		(Li and Manley, 2005; Tuduri et al., 2009)
SF3B2	Splicing factor 3B subunit 2	5.20	2.69E-03	III		(Tresini et al., 2015)
EIF4A	Eukaryotic initiation factor 4A	6.31	2.99E-04	III	*In vitro*	(Du et al., 2002)
FIP1L1	Pre-mRNA 3-end-processing factor FIP1	5.16	2.40E-04	III	Direct function in R-loop biology is not determined in human cells	(Stirling et al., 2012)

**DNA Topology**						
TOP1	DNA topoisomerase 1	4.61	1.83E-04	II		(Groh et al., 2014; Marinello et al., 2016; Marinello et al., 2013; Sollier et al., 2014; Sordet et al., 2009; Tuduri et al., 2009)
**Replication**						
MCM5	DNA replication licensing factor MCM5	5.00	3.71E-04	III	Murine B cell lines	(Wiedemann et al., 2016)
**Mitosis**						
BUB3	Mitotic checkpoint protein BUB3	5.52	3.29E-04	III		(Wan et al., 2015)
ZNF207	Zinc finger protein 207	4.71	4.14E-04	III		(Wan et al., 2015)

Supplemental experimental procedures

Oligonucleotide sequences

**Name**	**Sequence (5'⟶3')**
**β-actin gene**	
5'prom (F)	CCA CCT GGG TAC ACA CAG TCT
5'prom (R)	TGT CCT TGT CAC CCT TTC TTG
prom (F)	CCG AAA GTT GCC TTT TAT GGC
prom (R)	CAA AGG CGA GGC TCT GTG C
in1 (F)	CGG GGT CTT TGT CTG AGC
in1 (R)	CAG TTA GCG CCC AAA GGA C
in3(F)	TAA CAC TGG CTC GTG TGA CAA
in3(R)	AAG TGC AAA GAA CAC GGC TAA
in5(F)	GGA GCT GTC ACA TCC AGG GTC
in5(R)	TGC TGA TCC ACA TCT GCT GG
5'pause (F)	TTA CCC AGA GTG CAG GTG TG
5'pause (R)	CCC CAA TAA GCA GGA ACA GA
pause (F)	GGG ACT ATT TGG GGG TGT CT
pause (R)	TCC CAT AGG TGA AGG CAA AG
C (F)	TGG GCC ACT TAA TCA TTC AAC
C (R)	CCT CAC TTC CAG ACT GAC AGC
D (F)	CAG TGG TGT GGT GTG ATC TTG
D (R)	GGC AAA ACC CTG TAT CTG TGA

F (F)	CCA TCA CGT CCA GCC TAT TT
F (R)	TGT GTG AGT CCA GGA GTT GG
**γ-actin**	
prom (F)	GGA AAG ATC GCC ATA TAT GGA C
prom (R)	TCA CCG GCA GAG AAA CGC GAC
in1 (F)	CCG CAG TGC AGA CTT CCG AG
in1 (R)	CGG GCG CGT CTG TAA CAC GG
ex5(F)	GTG ACA CAG CAT CAC TAA GG
ex5 (R)	ACA GCA CCG TGT TGG CGT
A (F)	TTC GTG GGC TGG TGA GAA AA
A (R)	CTC CAA CAC CCA AAC CCA CT
B (F)	GGG TCA AGG GAT CGT TCT G
B (R)	GCC TGG AGC TCA GTA AGC
C (F)	GAG GTT TGA GAC TGC AGT GAG
C (R)	CAG ACA TAA TTT TGT GGG GTT TG
**Synthetic competitors for RNA/DNA hybrid IP**	
ssDNA (sense)	CGG TGT GAA TCA GAC
ssDNA (anti-sense)	GTC TGA TTC ACA CCG
ssRNA (sense)	CGG UGU GAA UCA GAC
ssRNA (anti-sense)	GUC UGA UUC ACA CCG
ds AU-rich RNA (sense)	AAU UAC AUU GAU AGA AUU AUU AG
ds AU-rich RNA (anti-sense)	CUA AUA AUU CUA UCA AUG UAA UU
**siRNA sequences**	All siRNAs are terminated by dTdT
control siRNA (siGENOME non-targeting siRNA#1, GE Life Science)	Sequence is licenced (D-001210-01)
DHX9 #1 siRNA (Thermo Fisher)	5'-GAAGUGCAAGCGACUCUAG-3'
DHX9 #2 siRNA (Thermo Fisher)	Sequence is licenced (s4019)
DHX9 #3 siRNA (GE Life Science)	Sequence is licenced (D-009950-01)
SETX siRNA (Invitrogen)	5'- AUUUGACGACGGCUUCCACCCAUUG-3'
Top1 siRNA (Thermo Fisher)	5'-GGACUCCAUCAGAUACUAU-3'
WHSC1 siRNA (Thermo Fisher)	5'- AACGGCCAGAACAAGCUCUUA -3'
SAFB2 siRNA (Thermo Fisher)	5'-GAGUCAGGAUCGCAAGUCA-3'
DNA-PK siRNA (Thermo Fisher)	5'-GGGCGCUAAUCGUACUGAA-3'
PARP1 siRNA (Thermo Fisher)	5'- GAAAGUGUGUUCAACUAAU-3'

siRNA and plasmid transfections

Transfection of plasmids and siRNAs was performed with Lipofectamine 2000 (Thermo Fisher Scientific) using the manufacturer's instructions. HeLa cells were transfected for 24 h and HEK293T cells for 48 h with pFlag (Sigma Aldrich, E7398), RNaseH1-Flag or RNaseH1-no tag plasmid (Groh et al., 2014). RNA interference was performed with Lipofectamine 2000 (Thermo Fisher Scientific) using the manufacturer's instructions. DHX9 and SETX siRNAs were transfected as described (Andersen et al., 2013).

Mass Spectrometry analysis

Peptides prepared using FASP were analysed on an Ultimate 3000 RSLCnano HPLC (Dionex, Camberley, UK) system run in direct injection mode coupled to a QExactive Orbitrap mass spectrometer (Thermo Electron, Hemel Hempstead, UK). Protein samples were resolved on a 25cm by 75 micron inner diameter picotip analytical column (New Objective, Woburn, MA, USA) which was packed in-house with ProntoSIL 120-3 C18 Ace-EPS phase, 3μm bead (Bischoff Chromatography, Germany). The system was operated at a flow-rate of 300nL min-1. A 120 min gradient was used to separate the peptides. The mass spectrometer was operated in a 'Top 20' data-dependent acquisition mode. Precursor scans were performed in the orbitrap at a resolving power of 70,000, from which the twenty most intense precursor ions were selected by the quadrupole and fragmented by HCD at a normalised collision energy of 30%. The quadrupole isolation window was set at 1.6 m/z. Charge state +1 ions and undetermined charge state ions were rejected from selection for fragmentation. Dynamic exclusion was enabled for 27s. Mass spectrometry data processing for all figures (except Figure S2B) was carried out using MaxQuant 1.5.0.35 and Andromeda search engine (Cox and Mann, 2008; Cox et al., 2011). Enzyme specificity was set to trypsin/P, allowing a maximum of two missed cleavages. Cysteine carbamidomethylation was selected as fixed and protein N-terminal acetylation and methionine oxidation as variable modifications. Initial mass tolerance of precursor ions was set to 50 ppm. Proteins and peptides were identified with FDR < 0.01 with a minimum peptide length of 7 amino acids. Protein identification required one unique peptide to the protein group. For protein quantification a minimum of two ratio counts were set and 'match between runs' function enabled. The initial 848 identified proteins were filtered for occurrence in at least three samples using Perseus 1.5.2.6. Common contaminants such as keratins and proteins of the large and small ribosomal subunits (RPL and RPS) were filtered out, due to their known contribution as contaminants and unresolved interactions in affinity purification procedures (Mellacheruvu et al., 2013).

For additional validation of the RNA/DNA hybrid interactome, an independent mass spectrometry quantitation pipeline was used, which is based on MS/MS spectra rather than ion intensities. For this, data were converted from .RAW to .MGF using ProteoWizard (Chambers et al., 2012). Data were analysed using the Central Proteomics Facility Pipeline software (Trudgian et al., 2010). Peptide searches were performed using the InterProphet meta-search combining Mascot, X! Tandem with the k-score plugin, and OMSSA against concatenated target/decoy sequence databases. Proteins were identified with at least two peptide sequences, with at least one unique peptide, FDR<1%. Relative label-free quantitation of proteins was carried out using the normalised spectral index implemented in the SINQ software (Trudgian et al., 2011).

The mass spectrometry proteomics data have been deposited to the ProteomeXchange Consortium via the PRIDE partner repository (Vizcaino et al., 2013) with the dataset identifier PXD002960 (www.ebi.ac.uk/pride/archive/login). Username: reviewer61059@ebi.ac.uk; Password: 3EpnlBv8.

Bioinformatical analyses

Statistical analysis of the mass spectrometry data from RNA/DNA hybrid IP is based on 3 independent biological replicates and was performed essentially as described elsewhere (Castello et al., 2012; Kwon et al., 2013). In short, intensity values were log_2-transformed and missing values were imputed with random numbers from a normal distribution to simulate low abundance values below noise level in Perseus 1.5.2.6, as described (Raschle et al., 2015). Using the limma package in R/Bioconductor, a linear model was fitted to these data to calculate the log_2 enrichment between RNA/DNA hybrid IP and control samples. An empirical Bayes moderated t-test was used to calculate p-values (Smyth, 2004) . P-values were then corrected for multiple testing by Benjamini-Hochberg method. Proteins enriched in RNA/DNA hybrid IP compared to control were included in the "RNA/DNA hybrid Interactome" if their corrected p-values < 0.01.

Cellular compartment analysis was based on GO term cellular component analysis. Fisher's exact test was used to calculate statistical enrichment, using Benjamini-Hochberg correction for multiple testing. Proteins were classified into the groups 'nucleus', 'nucleolus', 'nucleoplasm' and 'cytoplasm', with the latter group consisting of proteins that are exclusively cytoplasmic.

Overrepresentation analysis of protein classes was performed using the PANTHER database (www.pantherdb.org), version 10.0 (Mi et al., 2013). PANTHER protein classes overrepresented in the RNA/DNA hybrid IP were ranked according to their Benjamini-Hochberg-corrected p-values (p-value threshold 0.05) and the 17 most-significant groups were manually curated for redundancy.

Venn diagram representation of the overlap of RNA/DNA hybrid interactome with the HeLa mRNA interactome is based on (Castello et al., 2012). Two subgroups, proteins identified both in RNA/DNA hybrid interactome and HeLa interactome, and proteins exclusively identified in RNA/DNA hybrid interactome, were further examined for overrepresented protein classes using PANTHER, as described above.

Chromatin probability assignment was based on previously published data (Kustatscher et al., 2014) by assigning Interphase Chromatin Probability (ICP) values to the proteins in the RNA/DNA hybrid interactome. As comparison, ICP values are shown for the whole list of 7635 HeLa proteins as provided in (Kustatscher et al., 2014).

Genetic alterations of helicases identified in RNA/DNA hybrid interactome was carried out using the COSMIC database (cancer.sanger.ac.uk) (Forbes et al., 2015). In brief, total number of copy number variations (either gain or loss) were retrieved for each gene and expressed as percentage of total cancer samples annotated in the database, independently of the tissue. Data were plotted alongside two tumour suppressor genes and oncogenes. Differential mRNA expression analysis in cancer was performed using the ONCOMINE platform (www.oncomine.org) (Rhodes et al., 2007), with a p-value threshold of < 0.05 and a minimal fold-change of 2 between cancer and matched control samples. Cancer case studies with significant alterations were grouped based on whether the gene was amongst the top 1%, top 5%, or top 10% of all altered genes.

Antibodies

The following antibodies were used for ChIP: DHX9 (Abcam, ab26271, Lot#GR83942 and #187365), γH2AX (Millipore, 07-164), H2AX (Millipore, 07-627), H3 (Abcam, ab1791), PARP1 (Abcam ab6079, Proteintech 22999-1-AP) and Pol II (Santa Cruz, H-224). Antibodies used for IP, western blotting and IF: actin (Sigma, A2066), CBP80 (sc-48803, Santa Cruz), DDX5 (Bethyl, A300-523A), DDX1 (Proteintech, 11357-1-AP), DHX9 (Abcam, ab26271), DNA-PKcs (Abcam, ab1832), Drosha (Cell Signalling, D28B1), γH2AX (Millipore, 05-636), H3 (Abcam, ab1791), IgG2a (M5409, Sigma), Lamin B1 (Abcam, ab16048), Nuclear Pore Complex (Abcam, ab24609), anti-PAR (Trevigen, 4336-BPC-100), PARP1 (Abcam ab32138), RNA Pol II (Abcam, ab817), RNase H1 (Proteintech 156061-AP), SAFB2 (A301-113A-T), SETX (Bethyl, A301-105A), SRSF1 (LifeTechnologies, Clone 96), Topoisomerase I (Abcam, ab109374), alpha-tubulin (Sigma, T5168), WHSC1 (Abcam, ab75359), XRN2 (Proteintech, 11267-1-AP).

RNA/DNA hybrid slot blot

The slot blot was performed as described (Kotsantis et al., 2016; Sollier et al., 2014). RNase H sensitivity was carried out by incubation with 2 U of RNase H (NEB, M0297) per ug of genomic DNA for 2.5 h at 37 °C. Images were acquired with LAS-4000 (Fujifilm) (Figure 3) or by chemiluminescence using autoradiography in other figures. S9.6 signal was quantified using Image Studio Lite software (Li-COR Biosciences).

Immunoprecipitation and protein analysis

HeLa cells at 85% confluency were washed with PBS and lysed in RSB+T (10 mM TRIS pH 7.5, 200 mM NaCl, 2.5mM $MgCl_2$, 0.5% Triton X-100) on ice, followed by brief sonication (Diagenode Bioruptor). After removal of insoluble material, 1 mg of extracts were incubated with 3 ug of antibodies overnight. Immuno-complexes were captured with protein A dynabeads (Invitrogen), washed in RSB+T and eluted as described for RNA/DNA hybrid IP.

To prepare whole cell extracts, cells were lysed in RIPA buffer or in 1% SDS and 10 mM Tris-HCl (pH 7.4) buffer as described (Cristini et al., 2016), supplemented with protease inhibitor cocktail (Roche). Silver staining of SDS-PAGE gels was carried as in (Green and Sambrook, 2012).

RNA/DNA hybrid IP with RNase H treatment

Genomic DNA containing RNA/DNA hybrids was isolated as described before (Groh et al., 2014) and treated with 5.5 U of RNase H (NEB, M0297) per ug of DNA overnight at 37 °C. DNA was sonicated for 10 min (Diagenode Bioruptor) prior to RNase H treatment for IP and western blot analysis or left unsonicated for the slot blot. A fraction of the genomic DNA was stored as ‘genomic DNA Input’ for the slot blot. Genomic DNA (4 ug for western blot and 30 ug for the slot blot) was enriched for RNA/DNA hybrids by immuno-precipitation with S9.6 antibody, bound to protein A dynabeads (Invitrogen), pre-blocked with 0.5% BSA/PBS, for 2 h at 4 °C. Beads were washed 3x with RSB+T and incubated for 2 h at 4 °C with diluted HeLa nuclear extracts containing 15 ug proteins, prepared as described for RNA/DNA hybrid IP and pre-treated with 0.1 mg/ml RNase A (PureLink, Invitrogen) for 1 h at 37 °C to degrade all RNA/DNA hybrids (Figure S3A). Excess of RNase A was blocked by adding 200 U of

RNasin (Promega) to IPs. 100 ul fraction of diluted and RNase A pre-treated extracts prior to IP was stored as 'Protein Input' for western blot. Bead washes and elution were performed as described for RNA/DNA hybrid IP.

Immunofluorescence microscopy (IF)

For DHX9 IF, cells were fixed with 3% PFA, washed, permeabilised and blocked in PBS, 1% goat serum, 0.5% triton X-100, followed by incubation with DHX9 antibody. γH2AX and RNase H1 IF were carried out as described (Cristini et al., 2016; Sordet et al., 2009). For S9.6 IF, cells were fixed with ice-cold methanol for 10 min at -20 °C, washed with PBS and permeabilized with Triton 0.1% for 10 min at RT. After PBS washes, slides were treated with 150 U/ml of RNase H (NEB, M0297) or left untreated for 36 h at 37 °C. After PBS washes, slides were blocked with 8% BSA before incubating with purified S9.6 antibody overnight at 4 °C. Slides were incubated with appropriate secondary antibodies, coupled to Alexa Fluor 488 or 594 (Invitrogen), and mounted using Vectashield with DAPI (Vector Laboratories). Images were acquired on an Axioplan 2e (Zeiss), on a confocal Olympus FV1200 or on a confocal Zeiss 880 Airyscan. Fluorescence intensities were quantified with ImageJ (version 1.50g or 1.51k).

RNA analysis

Total RNA was harvested using TRIZOL reagent (Invitrogen) followed by DNase I treatment (Roche). 1-2 µg of total RNA was reverse-transcribed using SuperScript Reverse Transcriptase III (Invitrogen) with random hexamers (Invitrogen) and analysed by quantitative PCR with QuantiTect SYBR green (Qiagen).

Supplemental References

Andersen, P.R., Domanski, M., Kristiansen, M.S., Storvall, H., Ntini, E., Verheggen, C., Schein, A., Bunkenborg, J., Poser, I., Hallais, M., *et al.* (2013). The human cap-binding complex is functionally connected to the nuclear RNA exosome. Nat Struct Mol Biol *20*, 1367-1376.

Chambers, M.C., Maclean, B., Burke, R., Amodei, D., Ruderman, D.L., Neumann, S., Gatto, L., Fischer, B., Pratt, B., Egertson, J., *et al.* (2012). A cross-platform toolkit for mass spectrometry and proteomics. Nat Biotechnol *30*, 918-920.

Chen, L., Chen, J.Y., Huang, Y.J., Gu, Y., Qiu, J., Qian, H., Shao, C., Zhang, X., Hu, J., Li, H., *et al.* (2018). The Augmented R-Loop Is a Unifying Mechanism for Myelodysplastic Syndromes Induced by High-Risk Splicing Factor Mutations. Molecular cell *69*, 412-425 e416.

Chen, E.Y., Tan, C.M., Kou, Y., Duan, Q., Wang, Z., Meirelles, G.V., Clark, N.R., and Ma'ayan, A. (2013). Enrichr: interactive and collaborative HTML5 gene list enrichment analysis tool. BMC Bioinformatics *14*, 128.

Cox, J., Neuhauser, N., Michalski, A., Scheltema, R.A., Olsen, J.V., and Mann, M. (2011). Andromeda: a peptide search engine integrated into the MaxQuant environment. J Proteome Res *10*, 1794-1805.

Du, M.X., Johnson, R.B., Sun, X.L., Staschke, K.A., Colacino, J., and Wang, Q.M. (2002). Comparative characterization of two DEAD-box RNA helicases in superfamily II: human translation-initiation factor 4A and hepatitis C virus non-structural protein 3 (NS3) helicase. Biochem J *363*, 147-155.

Green, M., and Sambrook, J. (2012). Molecular Cloning: A Laboratory Manual, 4th edn (Cold Spring Harbour Laboratory Press, Cold Spring Harbour, NY).

Hill, S.J., Mordes, D.A., Cameron, L.A., Neuberg, D.S., Landini, S., Eggan, K., and Livingston, D.M. (2016). Two familial ALS proteins function in prevention/repair of transcription-associated DNA damage. Proc Natl Acad Sci U S A *113*, E7701-E7709.

Kwon, S.C., Yi, H., Eichelbaum, K., Fohr, S., Fischer, B., You, K.T., Castello, A., Krijgsveld, J., Hentze, M.W., and Kim, V.N. (2013). The RNA-binding protein repertoire of embryonic stem cells. Nat Struct Mol Biol *20*, 1122-1130.

Li, L., Germain, D.R., Poon, H.Y., Hildebrandt, M.R., Monckton, E.A., McDonald, D., Hendzel, M.J., and Godbout, R. (2016). DEAD Box 1 Facilitates Removal of RNA and Homologous Recombination at DNA Double-Strand Breaks. Mol Cell Biol *36*, 2794-2810.

Li, L., Monckton, E.A., and Godbout, R. (2008). A role for DEAD box 1 at DNA double-strand breaks. Mol Cell Biol *28*, 6413-6425.

Marinello, J., Chillemi, G., Bueno, S., Manzo, S.G., and Capranico, G. (2013). Antisense transcripts enhanced by camptothecin at divergent CpG-island promoters associated with bursts of topoisomerase I-DNA cleavage complex and R-loop formation. Nucleic acids research *41*, 10110-10123.

Mellacheruvu, D., Wright, Z., Couzens, A.L., Lambert, J.P., St-Denis, N.A., Li, T., Miteva, Y.V., Hauri, S., Sardiu, M.E., Low, T.Y., *et al.* (2013). The CRAPome: a contaminant repository for affinity purification-mass spectrometry data. Nat Methods *10*, 730-736.

Mi, H., Muruganujan, A., Casagrande, J.T., and Thomas, P.D. (2013). Large-scale gene function analysis with the PANTHER classification system. Nat Protoc *8*, 1551-1566.

Nadel, J., Athanasiadou, R., Lemetre, C., Wijetunga, N.A., P, O.B., Sato, H., Zhang, Z., Jeddeloh, J., Montagna, C., Golden, A., *et al.* (2015). RNA:DNA hybrids in the human genome have distinctive nucleotide characteristics, chromatin composition, and transcriptional relationships. Epigenetics Chromatin *8*, 46.

Raschle, M., Smeenk, G., Hansen, R.K., Temu, T., Oka, Y., Hein, M.Y., Nagaraj, N., Long, D.T., Walter, J.C., Hofmann, K., *et al.* (2015). DNA repair. Proteomics reveals dynamic assembly of repair complexes during bypass of DNA cross-links. Science *348*, 1253671.

Sollier, J., Stork, C.T., Garcia-Rubio, M.L., Paulsen, R.D., Aguilera, A., and Cimprich, K.A. (2014). Transcription-coupled nucleotide excision repair factors promote R-loop-induced genome instability. Molecular cell *56*, 777-785.

Song, C., Hotz-Wagenblatt, A., Voit, R., and Grummt, I. (2017). SIRT7 and the DEAD-box helicase DDX21 cooperate to resolve genomic R loops and safeguard genome stability. Genes & development.

Sridhara, S.C., Carvalho, S., Grosso, A.R., Gallego-Paez, L.M., Carmo-Fonseca, M., and de Almeida, S.F. (2017). Transcription Dynamics Prevent RNA-Mediated Genomic Instability through SRPK2-Dependent DDX23 Phosphorylation. Cell Rep *18*, 334-343.

Tresini, M., Warmerdam, D.O., Kolovos, P., Snijder, L., Vrouwe, M.G., Demmers, J.A., van, I.W.F., Grosveld, F.G., Medema, R.H., Hoeijmakers, J.H., *et al.* (2015). The core spliceosome as target and effector of non-canonical ATM signalling. Nature *523*, 53-58.

Trudgian, D.C., Thomas, B., McGowan, S.J., Kessler, B.M., Salek, M., and Acuto, O. (2010). CPFP: a central proteomics facilities pipeline. Bioinformatics *26*, 1131-1132.

Vizcaino, J.A., Cote, R.G., Csordas, A., Dianes, J.A., Fabregat, A., Foster, J.M., Griss, J., Alpi, E., Birim, M., Contell, J., *et al.* (2013). The PRoteomics IDEntifications (PRIDE) database and associated tools: status in 2013. Nucleic acids research *41*, D1063-1069.

Vukovic, L., Koh, H.R., Myong, S., and Schulten, K. (2014). Substrate recognition and specificity of double-stranded RNA binding proteins. Biochemistry *53*, 3457-3466.

Wan, Y., Zheng, X., Chen, H., Guo, Y., Jiang, H., He, X., Zhu, X., and Zheng, Y. (2015). Splicing function of mitotic regulators links R-loop-mediated DNA damage to tumor cell killing. J Cell Biol *209*, 235-246.

Wang, X., Schwartz, J.C., and Cech, T.R. (2015). Nucleic acid-binding specificity of human FUS protein. Nucleic acids research *43*, 7535-7543.

Wiedemann, E.M., Peycheva, M., and Pavri, R. (2016). DNA Replication Origins in Immunoglobulin Switch Regions Regulate Class Switch Recombination in an R-Loop-Dependent Manner. Cell Rep *17*, 2927-2942.
