# Supplementary material for: RNA/DNA Hybrid Interactome Identifies DXH9 as a Molecular Player in Transcriptional Termination and R-Loop-Associated DNA Damage
Source: Cell Rep. 2018 May 8;23(6):1891–905. doi: 10.1016/j.celrep.2018.04.025 (PMC5976580; doi:10.1016/j.celrep.2018.04.025)
Supplement: Document S2. Article plus Supplemental Information [file mmc3.pdf]

Cell Reports

Resource

RNA/DNA Hybrid Interactome Identifies DXH9 as a Molecular Player in Transcriptional Termination and R-Loop-Associated DNA Damage

Graphical Abstract

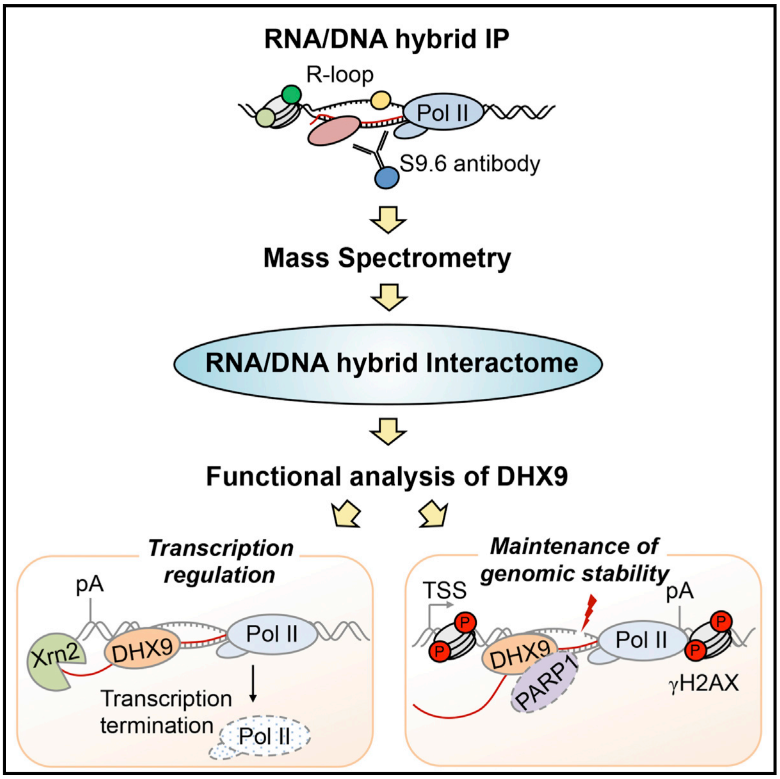

Authors

Agnese Cristini, Matthias Groh, Maiken S. Kristiansen, Natalia Gromak

Correspondence

natalia.gromak@path.ox.ac.uk

In Brief

Cristini et al. use affinity purification and mass spectrometry to define the RNA/DNA interactome in human cells and validate it by revealing the role of DHX9 in transcriptional termination and R-loop-associated DNA damage.

Highlights

- Mass spectrometry identifies the RNA/DNA hybrid interactome in human cells
- Top RNA/DNA interactome candidate DHX9 promotes R-loop suppression
- DHX9 regulates transcriptional termination
- DHX9 interacts with PARP1 and prevents R-loop-associated DNA damage

Data and Software Availability

PXD002960

Cristini et al., 2018, Cell Reports *23*, 1891–1905
May 8, 2018 © 2018 The Author(s).
https://doi.org/10.1016/j.celrep.2018.04.025

CellPress

OPEN ACCESS
CellPress

RNA/DNA Hybrid Interactome Identifies DXH9 as a Molecular Player in Transcriptional Termination and R-Loop-Associated DNA Damage

Agnese Cristini,[1,2] Matthias Groh,[1,2] Maiken S. Kristiansen,[1,3] and Natalia Gromak[1,4,*]
[1]Sir William Dunn School of Pathology, University of Oxford, South Parks Road, Oxford OX1 3RE, UK
[2]These authors contributed equally
[3]Present address: MRC Molecular Haematology Unit, Weatherall Institute of Molecular Medicine, John Radcliffe Hospital, University of Oxford, Oxford OX3 9DS, UK
[4]Lead Contact
*Correspondence: natalia.gromak@path.ox.ac.uk
https://doi.org/10.1016/j.celrep.2018.04.025

SUMMARY

R-loops comprise an RNA/DNA hybrid and displaced single-stranded DNA. They play important biological roles and are implicated in pathology. Even so, proteins recognizing these structures are largely undefined. Using affinity purification with the S9.6 antibody coupled to mass spectrometry, we defined the RNA/DNA hybrid interactome in HeLa cells. This consists of known R-loop-associated factors SRSF1, FACT, and Top1, and yet uncharacterized interactors, including helicases, RNA processing, DNA repair, and chromatin factors. We validate specific examples of these interactors and characterize their involvement in R-loop biology. A top candidate DHX9 helicase promotes R-loop suppression and transcriptional termination. DHX9 interacts with PARP1, and both proteins prevent R-loop-associated DNA damage. DHX9 and other interactome helicases are overexpressed in cancer, linking R-loop-mediated DNA damage and disease. Our RNA/DNA hybrid interactome provides a powerful resource to study R-loop biology in health and disease.

INTRODUCTION

R-loops consist of an RNA/DNA hybrid and a displaced non-template DNA strand. These structures are thermodynamically stable and can arise during transcription, where they contribute to gene regulation at multiple levels (Ginno et al., 2012; Skourti-Stathaki et al., 2011; Yang et al., 2014). They also are involved in immunoglobulin class switch recombination, DNA replication, and regulation of DNA and histone modifications (Aguilera and García-Muse, 2012; Skourti-Stathaki and Proudfoot, 2014).

Despite crucial biological processes associated with R-loops, many aspects of R-loop biology remain unclear. Which factors influence R-loop formation at different genomic locations? How are R-loop levels precisely controlled to allow for their beneficial functions while preventing detrimental effects from dysregulated R-loops? Failure to correctly control R-loop levels results in increased DNA damage and genome instability (Aguilera and García-Muse, 2012; Skourti-Stathaki and Proudfoot, 2014; Sollier and Cimprich, 2015) and aberrant transcriptional termination (Skourti-Stathaki et al., 2011, 2014). Recent studies have implicated R-loops in the pathology of human diseases (Groh and Gromak, 2014). R-loops can form at expanded trinucleotide DNA repeats, leading to heterochromatin formation and transcriptional repression of genes associated with neurological disorders, including amyotrophic lateral sclerosis/frontotemporal dementia (ALS/FTD), Friedreich ataxia, and fragile X syndrome (Colak et al., 2014; Groh et al., 2014; Haeusler et al., 2014). A growing body of evidence also connects R-loops to processes that are deregulated in cancer, including DNA repair, replication, and gene expression of tumor-promoting genes (Bhatia et al., 2014; Boque-Sastre et al., 2015; Hatchi et al., 2015; Kotsantis et al., 2016; Schwab et al., 2015; Stork et al., 2016; Yang et al., 2014).

Genetic screens have been used to identify factors that regulate R-loop levels in yeast (Chan et al., 2014; Stirling et al., 2012; Wahba et al., 2011). However, these screens could not distinguish direct or indirect effects of these factors on R-loops, and the molecular mechanisms linking these factors to R-loop biology are still not understood fully. Human cells possess dedicated enzymes that can directly bind and regulate R-loop levels, including members of the RNase H family that specifically degrade the RNA in R-loops (Cerritelli and Crouch, 2009) and the helicase senataxin (SETX) that can unwind RNA/DNA hybrids (Skourti-Stathaki et al., 2011). It is interesting that mutations in RNase H and SETX lead to devastating neurological disorders, further underlining the importance of maintaining the correct R-loop balance in human cells (Groh et al., 2017; Groh and Gromak, 2014). Considering the complexity of RNA-processing reactions and the high abundance of non-coding RNA transcription, R-loop formation is a likely consequence that will require the availability of protein factors to directly control R-loop levels.

We used an unbiased approach to purify RNA/DNA hybrids and identify their associated proteins by mass spectrometry (MS) in human cells. This RNA/DNA hybrid interactome comprises 469 proteins, including helicases, RNA/DNA-binding proteins, and factors implicated in DNA damage. More important, we identify and validate factors previously implicated in R-loop biology, including SRSF1 and Top1, as well as candidate

This is an open access article under the CC BY license (http://creativecommons.org/licenses/by/4.0/).

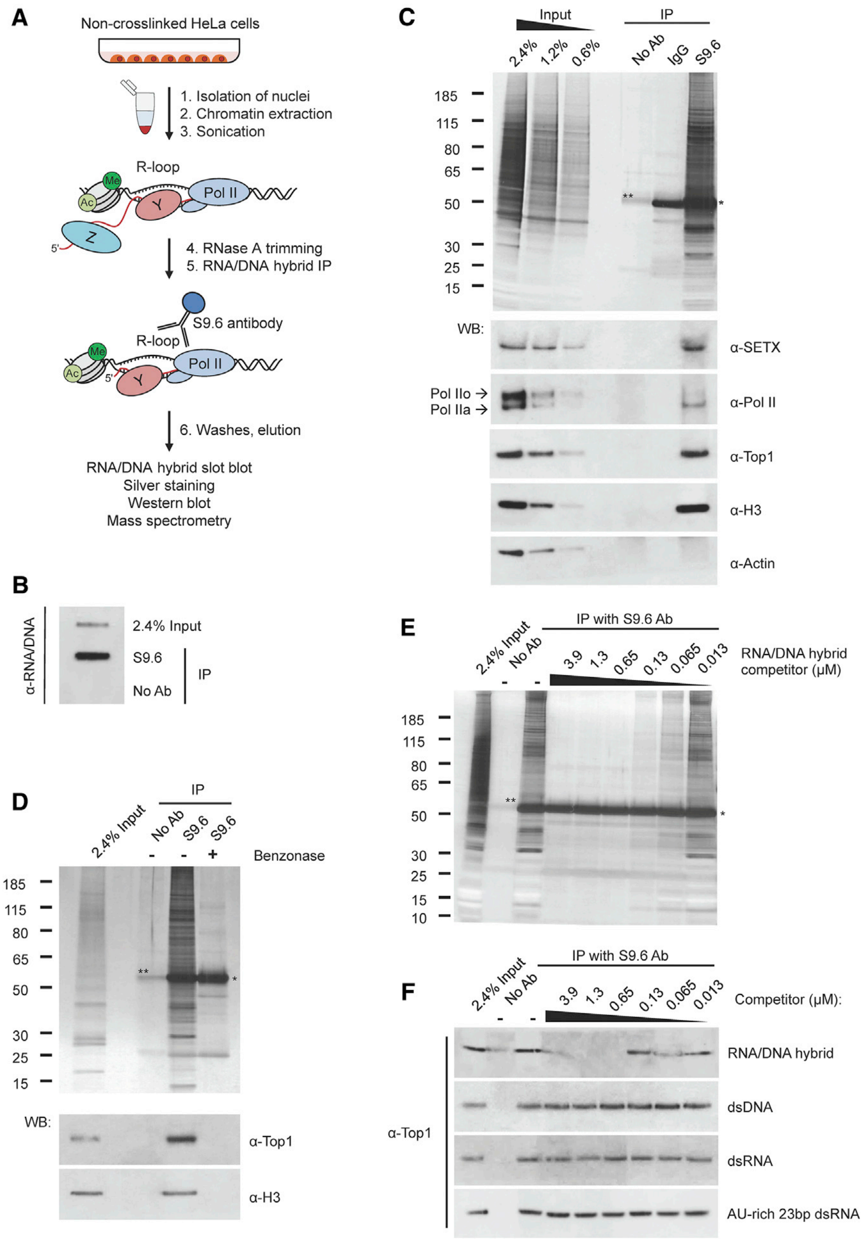

Figure 1. Design of RNA/DNA Hybrid IP Method in HeLa Cells

(A) RNA/DNA hybrid IP workflow in HeLa cells.

(B) RNA/DNA hybrid slot blot with S9.6 antibody.

(C) Top: silver stain of RNA/DNA hybrid IP. No antibody and isotype-matched IgG2a antibody were used as controls. Bottom: western blot of RNA/DNA hybrid IP using indicated antibodies. Arrows indicate hypophosphorylated (IIa) and hyperphosphorylated (IIo) forms of Pol II. Triple amounts of input and IP samples were loaded for SETX.

(D) Top: silver stain of RNA/DNA hybrid IP following benzonase treatment. Bottom: western blot of RNA/DNA hybrid IP, probed with Top1 and H3 antibodies.

(E) Silver stain of RNA/DNA hybrid IP in the presence of RNA/DNA hybrid competitor.

(F) Western blot for Top1 of RNA/DNA hybrid IP with indicated synthetic competitors.

(C–E) *, indicates the heavy chain of S9.6 and IgG2a antibodies. **, indicates BSA, used to block protein A dynabeads.

See also Figure S1.

RNA/DNA hybrid interactors DHX9, PARP1, SAFB2, WHSC1, and DNA-PK. We demonstrate that a top candidate DHX9 helicase prevents R-loop accumulation *in vivo* and is required for transcriptional termination. We also describe a role of DHX9 in maintaining genome stability. DHX9 interacts with PARP1, and the loss of these proteins leads to R-loop accumulation, which triggers DNA damage. DHX9 and other RNA/DNA hybrid-interacting helicases are overexpressed in cancers, highlighting their potential role in the cancer transcriptional program. In conclusion, our data show that the RNA/DNA hybrid interactome reveals new RNA/DNA hybrid-interacting proteins and provides a link between fundamental aspects of R-loop biology and human disease.

RESULTS

Design and Validation of the RNA/DNA Hybrid IP Method

To identify RNA/DNA hybrid-interacting factors in an unbiased way, we developed an affinity purification approach, using the S9.6 antibody, which specifically recognizes RNA/DNA hybrids with 0.6 nM affinity (Boguslawski et al., 1986; Phillips et al., 2013). Nuclear extracts were prepared from HeLa cells and sonicated before immunoprecipitation (IP) to minimize copurification of unspecific proteins (Figures 1A and S1A). Non-crosslinked cells were used because crosslinking reagents could induce R-loops (Schwab et al., 2015), preventing the identification of bona fide R-loop interactors. IP using the S9.6 antibody was carried out in the presence of RNase A to reduce unspecific RNA-mediated interactions and avoid S9.6 recognition of double-stranded RNA (dsRNA) (Figure S1B). RNase A treatment before IP did not affect RNA/DNA hybrid IP results, suggesting that R-loops are not formed artificially during the extraction procedure (data not shown). We verified that the RNA/DNA hybrid IP enriches for RNA/DNA hybrids using the RNA/DNA hybrid slot blot, which quantitatively detects both endogenous and synthetic RNA/DNA hybrids (Figures 1B and S1C). Silver staining of immunoprecipitated RNA/DNA hybrid-interacting proteins revealed a complex mixture of proteins, which differed from the nuclear input material (Figure 1C) or IP carried out with antibody-recognizing cap-binding protein 80 (CBP80) (Figure S1D). More important, little protein was detected in no antibody or

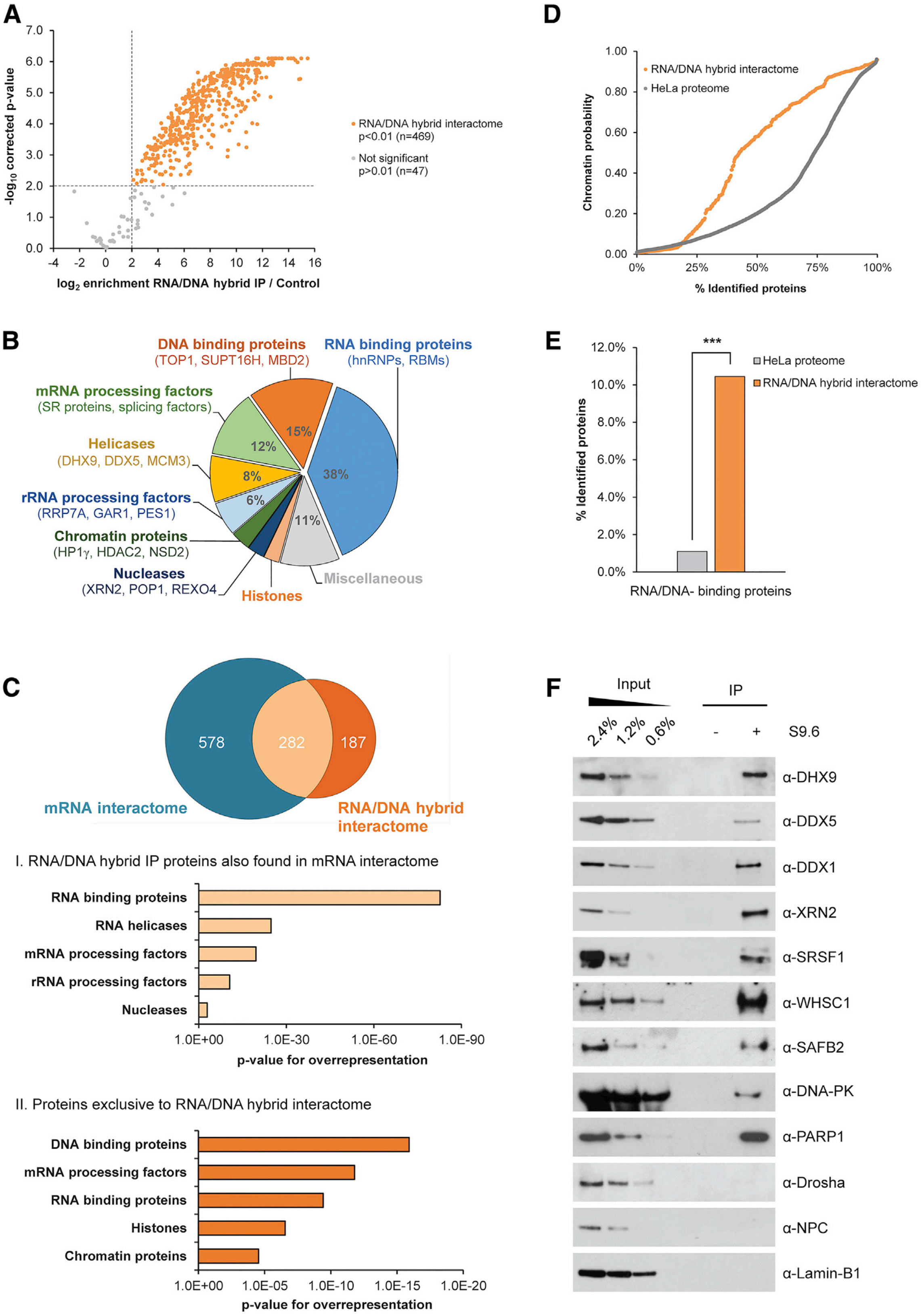

(legend on next page)

matched isotype immunoglobulin G2 (IgG2) negative IP controls. These results suggest that the RNA/DNA hybrid IP enriches a unique set of interacting proteins.

To test possible cross-reactivity of the S9.6 antibody with proteins, nuclear extracts were treated with benzonase, which degrades all forms of nucleic acids, including RNA/DNA hybrids, without affecting proteins (Figure S1E). Benzonase caused a loss of immunoprecipitated proteins (Figure 1D), confirming that RNA/DNA hybrid IP proteins are associated with nucleic acids recognized by the S9.6 antibody.

Next, we validated our IP procedure by testing copurification of proteins already implicated in R-loop biology. In particular, we detected the RNA/DNA helicase SETX, which resolves R-loops at termination regions (Figure 1C, bottom) (Hatchi et al., 2015; Skourti-Stathaki et al., 2011) and Top1 (El Hage et al., 2010; Tuduri et al., 2009). In line with cotranscriptional R-loop formation, RNA polymerase II (Pol II) also was detected. As an integral part of chromatin, RNA/DNA hybrids interacted with histone H3 but not with actin.

To verify that proteins identified in the RNA/DNA hybrid IP specifically bind RNA/DNA hybrids, we added synthetic 15–23 bp competitors during the IP procedure. When 15-bp RNA/DNA hybrid with 0.54 nM affinity for S9.6 antibody (Phillips et al., 2013) was added, a significant competition was observed for most concentrations, as demonstrated by a loss of copurified proteins, including Top1 and H3 (Figures 1E and 1F and S1F). In contrast, the addition of corresponding dsDNA, dsRNA, and 23 bp AU-rich dsRNA, which has low affinity for S9.6 *in vitro* (Phillips et al., 2013), did not affect RNA/DNA hybrid IP efficiency (Figures 1F and S1F–S1H). Supporting the specificity of our method, we did not detect the loss of proteins in the CBP80 IP in the presence of each competitor (Figure S1D).

In conclusion, even though a low affinity of S9.6 antibody with dsRNA in mild buffer conditions has been reported (Phillips et al., 2013), our stringent purification procedure and the use of RNase A ensures that the RNA/DNA hybrid IP method exhibits high specificity and efficiently enriches for proteins associated with RNA/DNA hybrids.

Characterization of the RNA/DNA Hybrid Interactome

We then developed a proteomic pipeline based on label-free quantitative MS to identify proteins associated with RNA/DNA hybrids in HeLa cells. To achieve the highest specificity, we compared protein enrichment in RNA/DNA hybrid IP to a control IP with S9.6 antibody in the presence of 1.3 μM RNA/DNA hybrid competitor. In total, 846 proteins were identified with high reproducibility ($r > 0.77$) in three biological replicates using MaxQuant software (Figure S2A) (Cox and Mann, 2008). Next, by using a moderated t test, we identified 469 candidate factors significantly enriched in the RNA/DNA hybrid versus control IP (Smyth, 2004). These proteins represent the RNA/DNA hybrid interactome (Figure 2A, orange data points). Analysis of our MS data with an independent approach based on the normalized spectral index (SIN) (Trudgian et al., 2011) found a strong correlation between the two methods ($r = 0.796$), confirming the robustness of the analysis (Figures S2B and S2C). We subdivided the RNA/DNA hybrid interactome into three classes according to their statistical significance: class I (top 25%), class II (next 50%), and class III (bottom 25%) (Figure S2D). A representative list of identified proteins, encompassing a variety of cellular functions, is provided in Table S2.

As expected, the RNA/DNA hybrid interactome almost exclusively contained nuclear proteins (Figure S2E). However, we observed no correlation between their cellular abundance (Geiger et al., 2012) and enrichment in the RNA/DNA hybrid interactome (Figure S2F), indicating that the RNA/DNA hybrid IP enriches for a specific subset of the proteome. Indeed, protein classes involved in RNA metabolism, such as heterogeneous nuclear ribonucleoproteins (hnRNPs), SR proteins and splicing factors, DNA-binding proteins (including Top1 and FACT), and chromatin-associated proteins, were overrepresented (Figure 2B). Notably, we detected many helicases acting on RNA (DDX5 and DHX9) or DNA (MCM3) and a high enrichment of RNA/DNA-binding domains, including RNA recognition motif (RRM); K homology (KH); double-stranded RNA-binding domains (dsRBDs); and SAF-A/B, acinus, and PIAS (SAP) (Figure S2G). This suggests that the RNA/DNA hybrid IP detects direct interactions rather than secondary interactions mediated by protein-protein binding.

We also investigated whether the RNA/DNA hybrid interactome exhibits additional characteristics. Comparison of the RNA/DNA hybrid interactome to the HeLa mRNA interactome (Castello et al., 2012) showed that despite an overlap between the two datasets, a significant part of the RNA/DNA hybrid interactome (187 proteins) is unique (Figure 2C, top). While proteins common to both interactomes are enriched for RNA-related functions, factors unique to the RNA/DNA hybrid interactome are involved in DNA and chromatin biology (Figure 2C, bottom).

Figure 2. Characterization of the RNA/DNA Hybrid Interactome

(A) Volcano plot displaying MS results of three biological replicates of RNA/DNA hybrid IP. Averaged $\log_2$ ratios between RNA/DNA hybrid IP and control IP (with addition of 1.3 μM synthetic RNA/DNA hybrid competitor) are plotted against their Benjamini-Hochberg-corrected $-\log_{10}$ p values across all replicates using a moderated t test. Proteins significantly enriched in RNA/DNA hybrid IP are in orange ($p < 0.01$) and constitute the RNA/DNA hybrid interactome. Proteins identified with $p > 0.01$ are in gray. Dashed lines indicate the significance cutoffs ($\log_2$ enrichment > 2 and $-\log_{10} > 2$).

(B) Protein classes overrepresented in RNA/DNA hybrid interactome (corrected $p < 0.05$, Fisher's exact test). Representative proteins are given in brackets.

(C) Top: overlap between mRNA interactome and RNA/DNA hybrid interactome in HeLa cells. Bottom: common proteins enriched in both RNA/DNA hybrid and mRNA interactomes (I) and proteins unique to the RNA/DNA hybrid interactome (II). The x axis indicates statistical significance of overrepresentation.

(D) The chromatin probability analysis of 7,635 HeLa proteins (HeLa proteome) and RNA/DNA hybrid interactome.

(E) Enrichment for proteins known to bind both RNA and DNA in the RNA/DNA hybrid interactome compared to the HeLa proteome ($p < 1.6 \times 10^{-28}$, Fisher's exact test).

(F) Validation of RNA/DNA hybrid interactors using western blot, probed with indicated antibodies. Drosha, NPC, and Lamin B1 are negative controls. (−) IP lane corresponds to control IP with IgG2a antibody.

See also Figure S2.

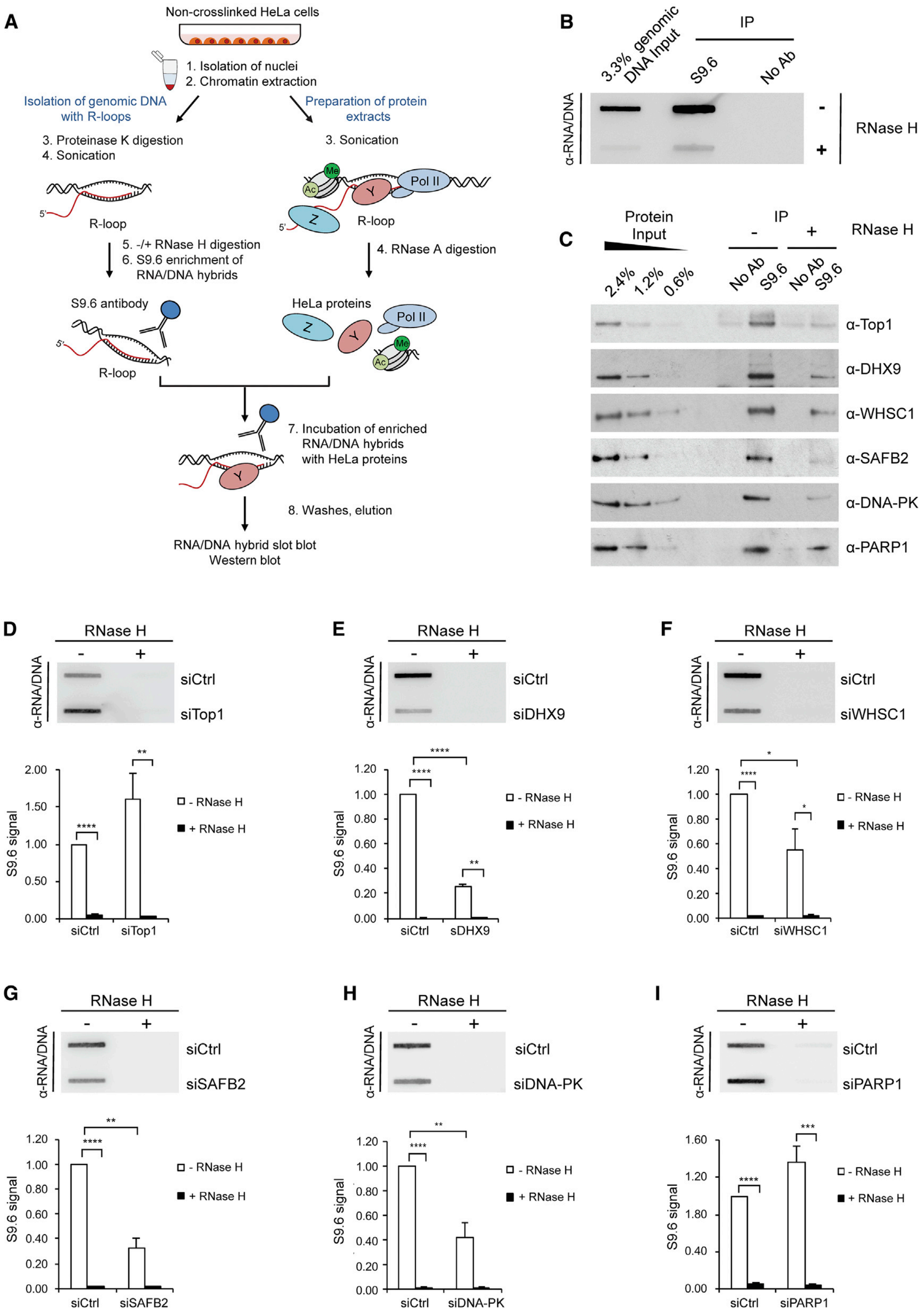

(legend on next page)

Consequently, we also detected an overall enrichment for proteins with high chromatin probability (Figure 2D) (Kustatscher et al., 2014) and proteins capable of binding to both RNA and DNA (Figure 2E) (Hudson and Ortlund, 2014) compared to the HeLa proteome. More important, there was no correlation between protein abundance in the RNA/DNA hybrid interactome and chromatin probability, demonstrating that our method enriches for a specific subset of proteins over the chromatin background (Figure S2H). It is interesting that the RNA/DNA hybrid interactome also was enriched for factors mediating genome stability (Paulsen et al., 2009) (Figure S2J), whereas other abundant protein classes were depleted (Figure S2I). Finally, consistent with the literature, we found a number of proteins already implicated in R-loop biology in human cells, including Top1 (El Hage et al., 2010; Tuduri et al., 2009), SRSF1 (Li and Manley, 2005; Tuduri et al., 2009), FACT (Herrera-Moyano et al., 2014), and some human counterparts of factors regulating R-loops in yeast, including ALY/REF (yeast YRA1) (Gavaldá et al., 2013) and DDX39B (yeast Sub2) (Gómez-González et al., 2011) (Table S3).

RNA/DNA Hybrid Interactome Uncovers New Candidates Involved in R-Loop Biology *In Vivo*

Next, we experimentally validated proteins identified in all three classes of the RNA/DNA hybrid interactome by western blotting (class I: DHX9, DDX5, WHSC1, SAFB2; class II: DDX1, XRN2, DNA-PK, PARP1; class III: SRSF1) (Figure 2F). In particular, we confirmed a number of proteins already implicated in R-loop biology, including the 5′-3′ exonuclease XRN2, previously associated with R-loop-mediated transcription termination (Skourti-Stathaki et al., 2011) and SRSF1 (Li and Manley, 2005; Tuduri et al., 2009). In addition, we validated candidates with previously unreported function in R-loop metabolism *in vivo*, including DHX9, DDX5, WHSC1, SAFB2, DNA-PK, and PARP1. We also confirmed the absence of abundant nuclear proteins, including Drosha, Lamin B1, and nuclear pore complex (NPC).

To confirm the specificity of these RNA/DNA hybrid interactors, we performed a modified version of the RNA/DNA hybrid IP with RNase H treatment (Figure 3A). The genomic DNA was extracted from HeLa cells and enriched for RNA/DNA hybrids with the S9.6 antibody. These genomic RNA/DNA hybrids were then incubated with HeLa nuclear extracts, depleted for RNA/DNA hybrids by treatment with a high concentration of RNase A, followed by IP of RNA/DNA-binding proteins with the S9.6 antibody (Figures 3B and 3C and S3A). All new R-loop-interacting candidates, including Top1, used as a positive control, can bind genomic RNA/DNA hybrids (Figure 3C). In contrast, treatment of the genomic DNA with RNase H strongly decreased the amount of copurified proteins, which correlated with the decrease in the RNA/DNA hybrid signal on the slot blot (Figures 3B and 3C).

To further investigate the involvement of selected candidates in R-loop biology, we performed slot blot analyses upon small interfering RNA (siRNA)-mediated depletion of these candidates (Figures S3C–3H). We observed that the knockdown of DHX9, WHSC1, SAFB2, and DNA-PK decreases the global level of RNA/DNA hybrids (Figures 3E–3H and S3B), whereas depletion of PARP1 and Top1 triggers an increase (Figures 3D and 3I). Moreover, the S9.6 signal was abolished by RNase H treatment, indicating that it is specific to RNA/DNA hybrids (Figures 3D–3I). Even though the mechanistic basis of the global R-loop changes requires further investigation, these results demonstrate that the RNA/DNA hybrid interactome uncovers new factors involved in R-loop biology.

DHX9 Promotes Transcriptional Termination

We detected several helicases enriched in the RNA/DNA interactome, suggesting that this protein class may play yet uncharacterized roles in R-loop biology. Therefore, we decided to further investigate the R-loop-associated function of a top validated candidate, DHX9. DHX9 has been shown to possess RNA/DNA helicase activity *in vitro* (Chakraborty and Grosse, 2011). To establish whether DHX9 associates with RNA/DNA hybrids *in vivo*, we confirmed the specificity of this interaction biochemically. RNA/DNA hybrid IP carried out with benzonase or RNase H treatments significantly reduced DHX9-RNA/DNA hybrid interaction (Figures S4A and 3C). Cell treatment with the transcriptional inhibitor actinomycin D also dramatically reduced DHX9-RNA/DNA hybrid interaction, suggesting that this interaction is transcription dependent (Figures 4A and 4B). Furthermore, DXH9-RNA/DNA hybrid interaction was prevented by synthetic RNA/DNA hybrid competitor but not by corresponding dsDNA, dsRNA, and AU-rich dsRNA competitors (Figure S4B). RNA/DNA hybrids also co-immunoprecipitated (coIP) with DHX9 but not with tubulin or no antibody control IPs (Figures 4C and 4D). These experiments confirm that DHX9 interacts with RNA/DNA hybrids *in vivo*.

We further examined the R-loop-associated biological functions of DHX9 *in vivo*. In line with its potential function in R-loop biology, endogenous DHX9 is predominantly localized throughout the nucleus (Figure 4E), and it is enriched at promoter-proximal regions of *β-actin* and *γ-actin* genes, as demonstrated by chromatin immunoprecipitation (ChIP) (Figures S4C and S4D). Therefore, we examined the R-loop profile of the *β-actin* and *γ-actin* genes in DHX9-depleted cells, using DNA/RNA immunoprecipitation (DIP) (Skourti-Stathaki et al., 2011).

Figure 3. Validation of New RNA/DNA Hybrid Interactome Candidates

(A) Workflow of RNA/DNA hybrid IP with RNase H digestion.

(B and C) HeLa genomic DNA input was either treated (+) or not (−) with RNase H before enrichment for RNA/DNA hybrids with the S9.6 antibody. Genomic RNA/DNA hybrids were incubated with nuclear extracts depleted for RNA/DNA hybrids with RNase A, followed by S9.6 IP. RNA/DNA hybrid slot blot (B) and western blot of RNA/DNA hybrid IP, probed with indicated antibodies (C).

(D–I) Genomic DNA from HeLa cells transfected with control (siCtrl) or indicated siRNAs was treated with RNase H. siTop1 (D), siDHX9 #1 (E), siWHSC1 (F), siSAFB2 (G), siDNA-PK (H), siPARP1 (I) were used. Top: RNA/DNA hybrid slot blot. Bottom: quantification of S9.6 signal. Values are normalized to the siCtrl and represent the means ± SEMs, n $\geq$ 3.

See also Figure S3.

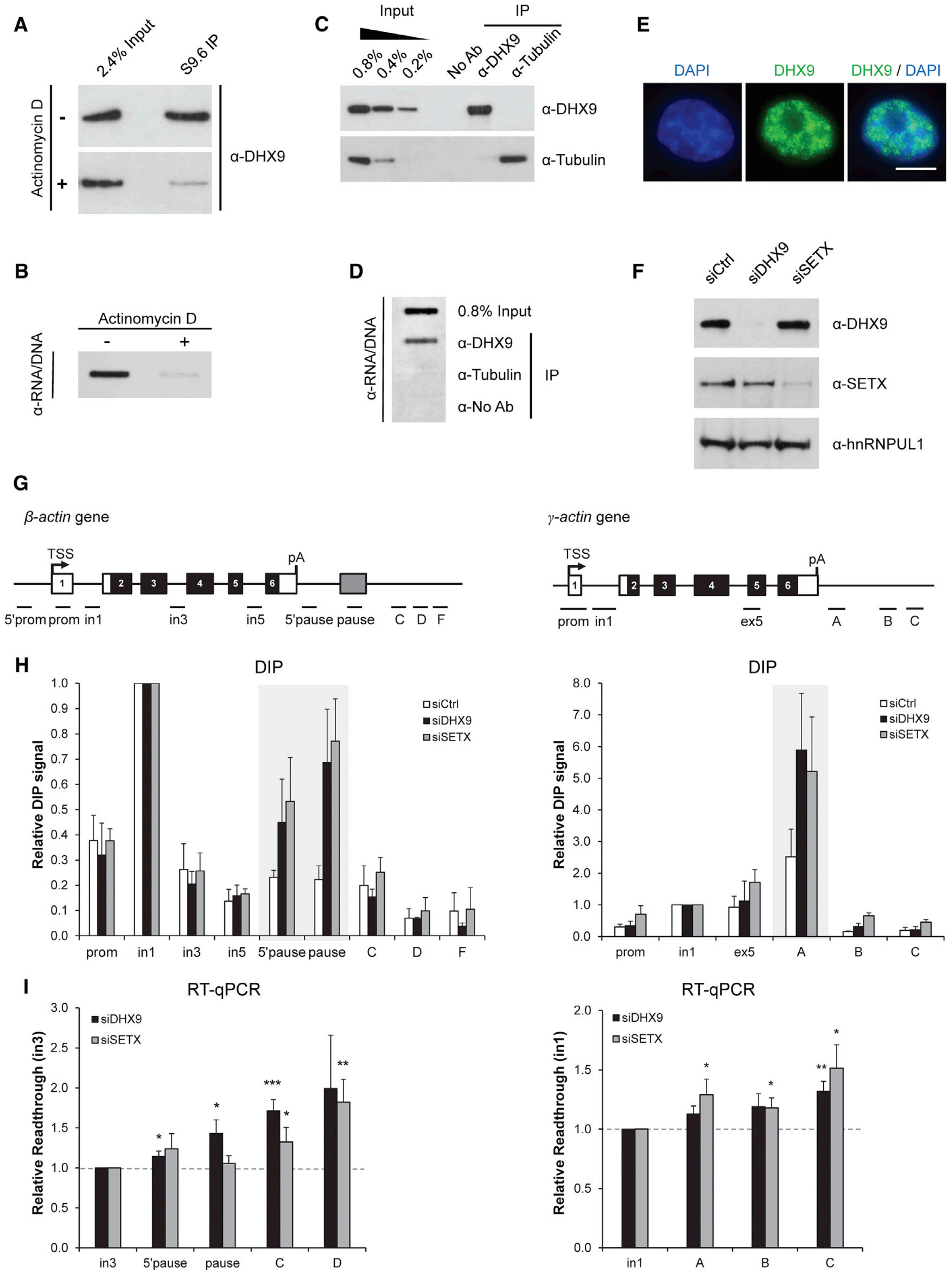

Figure 4. DHX9 Promotes Transcriptional Termination

(A and B) Western blot probed with DHX9 antibody (A) and RNA/DNA hybrid slot blot (B) of RNA/DNA hybrid IP from cells treated (+) or not (–) with actinomycin D.
(C and D) Western blot probed with DHX9 and tubulin antibodies (C) and RNA/DNA hybrid slot blot (D) of IPs carried out with DHX9 and tubulin antibodies.
(E) IF analysis of DHX9 (green). DAPI (blue) depicts nuclei. Scale bar, 10 μm.
(F) Western blot of HeLa protein extracts, treated with control, DHX9 #1, and SETX siRNAs and probed with indicated antibodies. hnRNPUL1 is a loading control.

(legend continued on next page)

As a positive control, we also depleted SETX, which resolves R-loops at the termination regions of the β-*actin* gene (Skourti-Stathaki et al., 2011). R-loops are enriched over the promoter-proximal and termination regions of the β-*actin* (in1, pause, and 5′ pause) (Skourti-Stathaki et al., 2011) and γ-*actin* (in1, A) genes (Figures 4G and 4H). R-loops were increased over the termination regions of both genes in DHX9- and SETX-depleted cells (Figures 4F–4H and S4E and S4F), and the DIP signal was sensitive to RNase H treatment (Figure S5E). These data suggested that similar to SETX, DXH9 promotes R-loop suppression at termination regions.

Next, we investigated whether the function of DHX9 in R-loop metabolism in poly(A)-proximal regions of β-*actin* and γ-*actin* genes can affect their transcriptional termination, as previously shown for SETX (Skourti-Stathaki et al., 2011). Similar to SETX, DHX9 depletion caused an increase in the amount of read-through transcripts downstream of the poly(A) signal (Figure 4I) and stabilization of Pol II over the transcription termination region for both genes (Figure S4G). These data pointed toward a role for DHX9 in R-loop suppression during transcriptional termination.

DHX9 Depletion Triggers R-Loop Accumulation in Response to CPT

Defects in transcription termination resulting from SETX or XRN2 depletion have been associated with R-loop accumulation and R-loop-driven genome instability (Hatchi et al., 2015; Morales et al., 2016). Because DHX9-depleted cells exhibit a termination defect (Figures 4H and 4I), we examined the potential role of DHX9 in preventing R-loop-associated DNA damage. For these studies, we used the Top1 inhibitor camptothecin (CPT), which is known to promote R-loop accumulation and replication- and transcription-associated DNA damage caused by unresolved DNA supercoiling (Marinello et al., 2016; Sordet et al., 2009). CPT treatment induced DNA damage, as indicated by the phosphorylation of histone variant H2AX (γH2AX) and R-loop accumulation as measured by immunofluorescence (IF) with the S9.6 antibody (Figures S5A and S5B).

Next, we examined the R-loop response following CPT treatment on the β-*actin* gene (Figure 5A). CPT triggered a reduction of R-loops over the prom and in1 regions, which correlated with transcriptional downregulation detected by Pol II ChIP (Figure S5C). This is in line with previously reported effects of CPT on Pol II transcription (Khobta et al., 2006). In contrast, CPT enhanced R-loop formation over in3, in5, and 5′ pause regions (Figure 5A). This coincided with the induction of γH2AX (Figure S5D). It is interesting that R-loops were most dramatically induced after 20 min, followed by a steady reduction, reaching the basal level at $\sim$240 min. These striking kinetics suggest that CPT-induced R-loops can be resolved efficiently by cellular factors. Therefore, we investigated the response of DHX9 to CPT-induced R-loop accumulation. ChIP analysis showed that despite a transcriptional downregulation (Figure S5C), DHX9 is enriched over the β-*actin* gene in response to CPT, and this binding is R-loop dependent because it is abolished by RNase H1 overexpression (Figures 5B and 5C). To test whether DHX9 is involved in the suppression of CPT-induced R-loops, we assessed R-loop levels in DHX9-depleted cells. In the absence of DHX9, CPT led to a dramatic increase in R-loops over in3, in5, and 5′ pause β-*actin* regions (Figure 5D). RNase H treatment confirmed that CPT-induced DIP peaks were specific (Figure S5E). Taken together, these results suggested that DHX9 is recruited to the β-*actin* gene to suppress CPT-induced R-loops.

DHX9 has been identified in MS screens for PARP1 and poly (ADP-ribos)ylation (PARylation) interactors (Gagné et al., 2012; Isabelle et al., 2010). We identified PARP1 in the RNA/DNA hybrid interactome by MS and validated its interaction with RNA/DNA hybrids *in vivo* (Figures 2F and 3C). Furthermore, we observed an increase in the global level of R-loops in PARP1-depleted cells (Figure 3I). Next, we examined whether PARP1 is involved in R-loop-driven DNA damage, similar to DHX9. CoIP experiments showed that endogenous DHX9 and PARP1 proteins interact in both untreated and CPT-treated cells (Figure 5E). Similar to DHX9, PARP1 was recruited to the β-*actin* gene in response to CPT (Figure 5F). Moreover, the CPT-induced R-loop signal was increased over in5 and 3′ end of the β-*actin* gene upon PARP inhibition with Olaparib (10 and 0.1 μM) or PARP1 depletion (Figures 5G and S6A–S6C). However, PARP inhibition did not further increase the R-loop signal on the in5 of the β-*actin* gene in DHX9-depleted cells, suggesting that DHX9 and PARP1 act in the same pathway to suppress CPT-induced R-loops (Figure S6D). Next, we tested whether DHX9 and PARP1 are required for their reciprocal recruitment to chromatin in response to CPT. Depletion of PARP1 did not compromise DHX9 recruitment to the β-*actin* gene (Figure S6E). Similarly, PARP1 recruitment was not affected upon DHX9 loss (Figure S6F). These results suggest that DHX9 and PARP1 promote the suppression of CPT-induced R-loops independent of their reciprocal recruitment to chromatin.

DHX9 Prevents R-Loop-Dependent DNA Damage in Response to CPT

An excess of R-loops promotes DNA damage and genome instability (Aguilera and García-Muse, 2012; Skourti-Stathaki and Proudfoot, 2014; Sollier and Cimprich, 2015). Therefore, we examined whether R-loop accumulation in DHX9-depleted cells caused a global increase in R-loop-dependent DNA damage by analyzing γH2AX signal by IF. In line with the increased accumulation of R-loops (Figure 5D), DHX9-depleted cells showed a significant increase in γH2AX signal following CPT treatment (Figure 6A). Inhibition of transcription with 5,6-dichloro-1-β-D-ribofuranosylbenzimidazole (DRB) or cordycepin clearly reduced γH2AX

(G) Diagram of β-*actin* (left) and γ-*actin* (right) genes depicting exons (black), UTRs (white), transcriptional start site (TSS), termination region (gray), and qPCR amplicons.

(H) DIP in HeLa cells, treated with control, DHX9 #1 and SETX siRNAs, on β-*actin* (left) and γ-*actin* (right) genes. Values are normalized to in1.

(I) Read-through transcription analysis of β-*actin* (left) and γ-*actin* (right) genes in HeLa cells treated with control, DHX9 #1, and SETX siRNAs, using RT-qPCR. Values are normalized to β-*actin* in3 and γ-*actin* in1.

(H–I) Bars, means $\pm$ SEMs, n $\geq$ 3.

See also Figure S4.

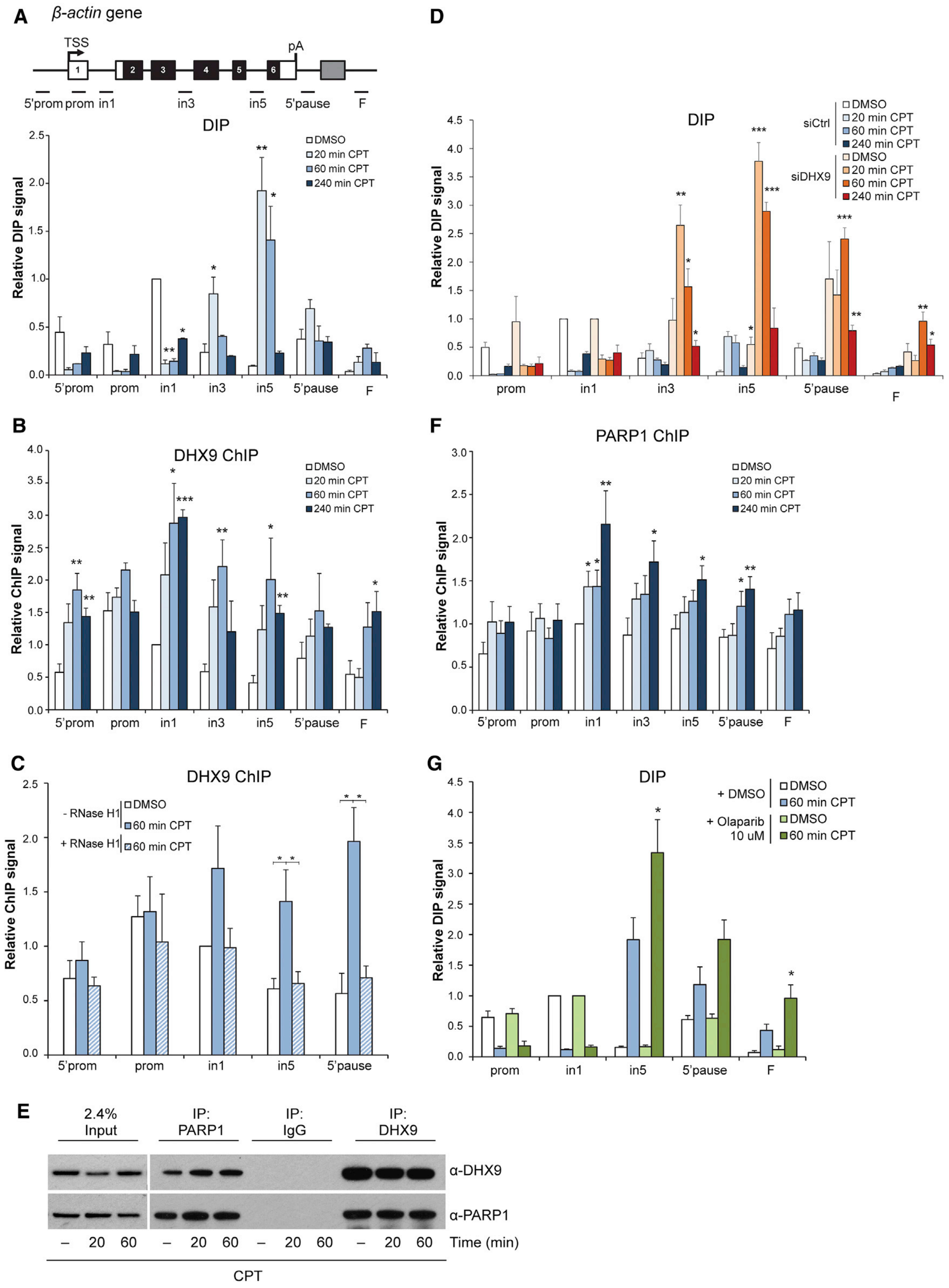

Figure 5. DHX9 Depletion Triggers R-Loop Accumulation in Response to CPT

(A, B, and F) Diagram of *β-actin* gene (A, top), DIP (A, bottom), DHX9 ChIP (B), and PARP1 ChIP (F) in HeLa cells, treated with CPT for the indicated time, on *β-actin* gene. Values are relative to in1 in the DMSO sample.

(C) DHX9 ChIP in HEK293T cells, transfected with FLAG (−RNase H1) or RNase H1 (+RNase H1) and treated with CPT for 60 min. Values are relative to in1 −RNase H1 in the DMSO sample.

(legend continued on next page)

induction, which is consistent with co-transcriptional formation of R-loops (Figures 6A and S7A). Furthermore, overexpression of RNase H1 significantly reduced the CPT-induced γH2AX signal in DHX9-depleted cells (Figure 6B). Co-staining against RNase H1 showed that cells overexpressing RNase H1 exhibited low γH2AX levels, while non-expressing cells showed high γH2AX signal (Figure S7B). Therefore, our data demonstrate that DHX9 prevents R-loop-associated DNA damage in response to CPT.

R-Loop Helicases and Cancer

The role of DHX9 in R-loop-associated DNA damage pointed toward its potential involvement in human cancer because one of cancer hallmarks is widespread genomic instability. In addition to DHX9, other DEAD/H helicases were enriched in the RNA/DNA hybrid interactome (Figure 7A), suggesting a general role for these helicases in R-loop processes. In agreement with a possible role for these RNA/DNA hybrid-interacting helicases in cancer, analysis of the COSMIC cancer dataset (Forbes et al., 2015) revealed that these DEAD/H helicases are frequently genetically amplified in cancer, similar to known oncogenes SKP2 and MDM2 (Figure 7B). Moreover, most identified helicases, including DHX9 and DDX5, showed mRNA overexpression in a range of cancers, based on the ONCOMINE database (Rhodes et al., 2007) (Figure 7C). Therefore, R-loop-interacting helicases may play a role in oncogenesis or cancer development.

DISCUSSION

In this study, we established and validated a specific affinity-based MS approach with the S9.6 antibody to define a comprehensive RNA/DNA hybrid interactome in HeLa cells (Figures 1 and 2). The RNA/DNA hybrid interactome represents a unique functional subset of the total HeLa proteome enriched for dual DNA- and RNA-binding proteins and RNA-processing factors. It comprises several proteins with previously described functions in R-loop biology, such as SRSF1, Top1, and FACT (Aguilera and García-Muse, 2012; Skourti-Stathaki and Proudfoot, 2014). The RNA/DNA hybrid interactome also reveals several new classes of *in vivo* RNA/DNA hybrid-binding proteins, including RNA-processing factors, helicases, histone modifiers, and DNA repair factors (Figure 2; Table S2), suggesting that these processes are linked to R-loops. It should be noted that some expected proteins, including SETX and RNase H1, were not identified by MS. This could be because of limited MS sensitivity for low-abundance proteins or their dynamic association with RNA/DNA hybrids *in vivo*. We experimentally validated a number of new candidates from the RNA/DNA hybrid interactome and demonstrated their possible implication in R-loop biology (Figure 3). Although future work is required to determine the specific function of these proteins in R-loop metabolism, these data highlight the value of the RNA/DNA hybrid interactome in understanding R-loop biology.

In this article, we specifically focused on R-loop-associated function of a top interactome candidate, helicase DHX9. Previous work demonstrated that DHX9 can resolve RNA/DNA hybrids *in vitro* (Chakraborty and Grosse, 2011). We report here that DHX9 interacts with RNA/DNA hybrids *in vivo* and it promotes R-loop suppression and transcriptional termination (Figure 4). Furthermore, DHX9 is important for maintaining genomic stability in response to CPT by preventing R-loop accumulation (Figures 5 and 6). However, it is not clear how DHX9 recognizes and suppresses physiological R-loops at transcription termination regions and CPT-induced R-loops.

DHX9 is involved in various aspects of RNA metabolism (Lee and Pelletier, 2016), and its depletion results in altered transcription (Chen et al., 2014). This broad involvement in transcription underlies the global decrease of R-loop levels detected by slot blot and IF upon DHX9 depletion (Figure 3). The new characterized role of DHX9 in transcriptional termination accounts for the specific R-loop accumulation at the termination regions of *β-actin* and *γ-actin* genes (Figure 4). In this respect, DHX9 behaves similarly to SETX (Skourti-Stathaki et al., 2011). DHX9 can regulate transcription either by binding directly to RNA (Liu et al., 2013) and gene promoters (Myöhänen and Baylin, 2001) or by mechanisms dependent on its nucleoside-triphosphatase (NTPase)/helicase activity (Nakajima et al., 1997) and its ability to coordinate protein-protein interactions (Anderson et al., 1998). Indeed, DHX9 interacts with a large number of proteins that function at the interface of transcription and DNA damage response, including RNA Pol II, CBP, BRCA1 (Anderson et al., 1998; Nakajima et al., 1997), SMN (Pellizzoni et al., 2001), DDX5 (Hegele et al., 2012), topoisomerase IIα (Zhou et al., 2003), and DNA-PK (Mischo et al., 2005). Therefore, DHX9 may be recruited to the promoters of transcribed genes by different mechanisms, and then, by interacting with Pol II, it can travel along the gene suppressing the arising R-loops.

Endogenous DHX9 is found in the same complexes as PARP1, another RNA/DNA hybrid interactor (Figure 5), which is in line with data from MS screens for PARP1 and PARylation interactors (Gagné et al., 2012; Isabelle et al., 2010). The loss of DHX9 and PARP1 independently results in increased R-loop accumulation following CPT treatment. An intriguing possibility is that DHX9 and PARP1 could be in the same pathway to suppress CPT-induced R-loops. PARP1 is not required for DHX9 recruitment to chromatin, but it may regulate DHX9 helicase activity in a similar way as it regulates the activity of the Werner syndrome helicase (von Kobbe et al., 2004). Moreover, our data show that following CPT treatment, PARP1 and DHX9 both are recruited in the body and at the 3′ end of the *β-actin* gene,

(D) DIP in HeLa cells, transfected with control (shades of blue) or DHX9 #1 (shades of red) siRNA and treated with CPT for indicated time, on the *β-actin* gene. Values are relative to in1 for each siRNA. The p-value is calculated for the siDHX9 versus the siCtrl sample.
(E) Western blot of IgG2a (negative control), PARP1, and DHX9 IPs in HeLa cells treated with CPT and probed with indicated antibodies. Left: input, right: IP.
(G) DIP in HeLa cells, treated with DMSO (shades of blue) or Olaparib (shades of green) before addition of CPT for 60 min, on the *β-actin* gene. Values are relative to in1 for DMSO and Olaparib. The pvalue is calculated for the Olaparib + CPT versus the DMSO + CPT samples.
(A–D, F, and G) Bars, means ± SEMs, n ≥ 3.
See also Figures S5 and S6.

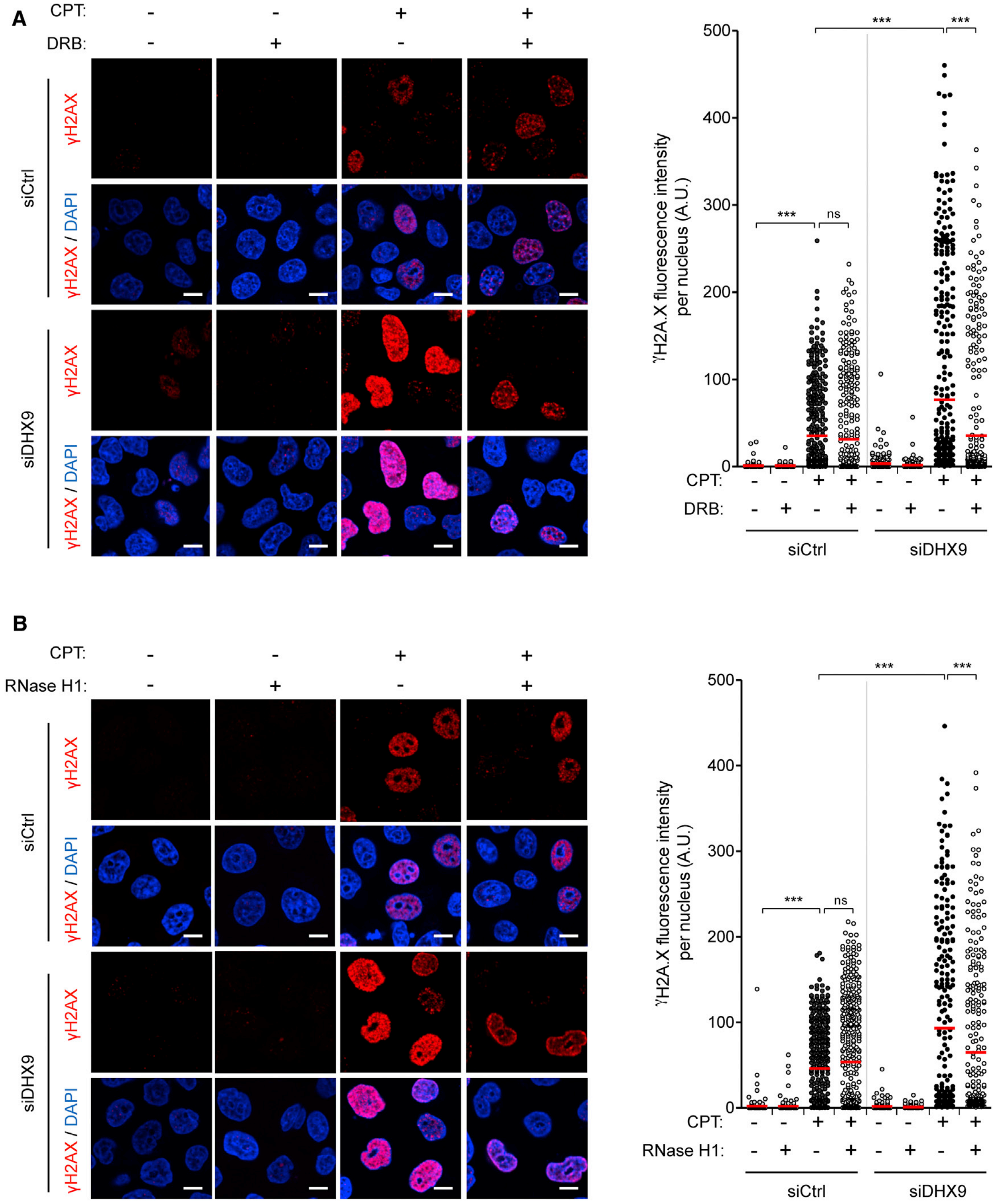

Figure 6. DHX9 Prevents R-Loop-Associated DNA Damage in Response to CPT

(A) IF analysis of HeLa cells transfected with control or DHX9 #1 siRNA and treated with DRB before the addition of CPT for 60 min and stained for γH2AX (red) and DAPI (blue). Left: representative images. Bars, 10 μm. Right: γH2AX fluorescence intensity per nucleus from a representative experiment (≥300 nuclei were analyzed per condition). The horizontal red bars represent the means, and each dot is one nucleus.

(B) The same as in (A), but instead of DRB, cells were transfected with FLAG (−RNase H1) or RNase H1 plasmid.

***$p < 0.001$, ns, not significant (one-way ANOVA Tukey's multiple comparisons test). See also Figure S7.

corresponding to R-loop peaks and the surrounding chromatin. The local increased concentration of R-loop-processing factors around the CPT-induced R-loops could be a way for the cells to deal with pathological R-loops, representing a threat to genome stability. However, PARP1 is known to play key roles in the repair of CPT-induced DNA lesions (Cristini et al., 2016; Das et al.,

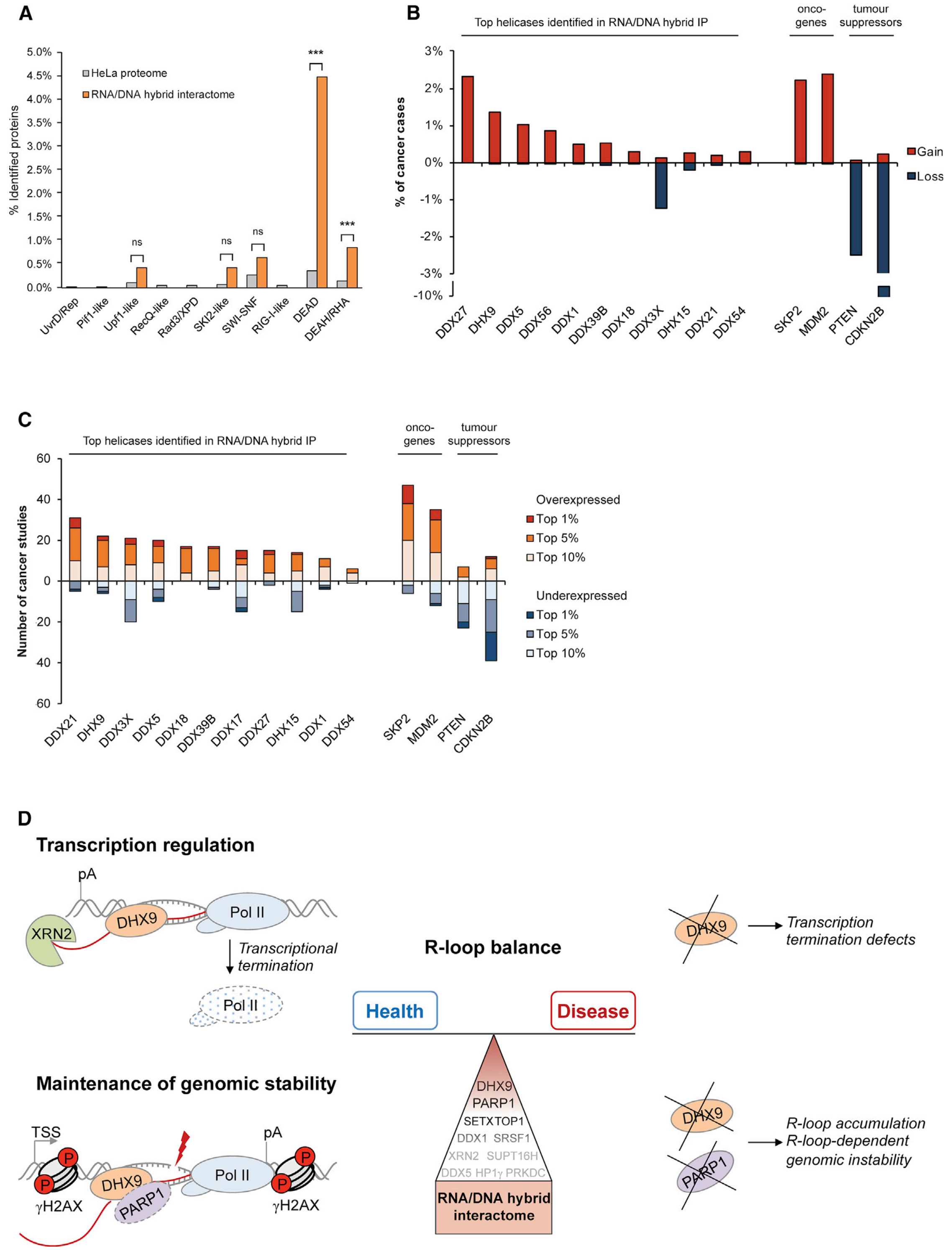

Figure 7. RNA/DNA Hybrid Interactome Helicases Are Amplified in Human Cancers

(A) Enrichment of DEAD and DEAH/RHA helicases in the RNA/DNA hybrid interactome.

(B) Gain (red) and loss (blue) of RNA/DNA hybrid-interacting DEAD/H helicases in cancer. The y axis shows the percentage of the total tested cancer samples based on copy number variations (COSMIC database).

(C) Transcriptional expression changes of RNA/DNA hybrid-interacting DEAD/H helicases in cancer. The y axis shows the number of cancer studies (ONCOMINE database).

(D) Model showing the role of the RNA/DNA hybrid interactome and the top candidate DHX9 in regulating R-loop balance in health and disease.

2014) and in other DNA repair pathways (Tallis et al., 2014). Therefore, following CPT treatment, PARP1 also could be recruited to the *β-actin* gene because of its function in DNA repair, independent of R-loop induction. This may explain the lack of exact co-localization between DHX9, PARP1, and CPT-induced R-loop peaks over the *β-actin* gene.

In addition to a potential model of cooperation between PARP1 and DHX9 in processing R-loops, different non-exclusive mechanisms could exist. First, PARP1 has many interactors that could process R-loops (Isabelle et al., 2010). Second, PARylation itself triggers the recruitment of RNA-processing factors such as FUS, TAF15, EWSF1, and SAFA to DNA damage (Britton et al., 2014). These factors are known R-loop suppressors (Aguilera and García-Muse, 2012; Skourti-Stathaki and Proudfoot, 2014). Finally, R-loop accumulation could be a consequence of impaired DNA damage repair. Indeed, R-loops have been described at DNA breaks (Britton et al., 2014; Cohen et al., 2018). Our data suggest a potential role of PARP1 in controlling R-loop balance, but given the complexity of this scenario, further studies are required to understand the mechanistic details underlying PARP1 function in R-loop metabolism.

Recently, it has been demonstrated that increased global transcription in response to oncogenes or hormones is a characteristic of cancer (Kotsantis et al., 2016; Stork et al., 2016). This transcriptional burst is accompanied by the accumulation of R-loops, which directly contributes to replication stress and genomic instability (Aguilera and García-Muse, 2012; Skourti-Stathaki and Proudfoot, 2014; Sollier and Cimprich, 2015). These findings suggest that an increased R-loop level could be a common feature of cancer cells. We found that multiple members of the DEAD/H helicase family, including DHX9, are strongly enriched in the RNA/DNA hybrid interactome and they are frequently deregulated in a range of cancers (Figure 7). These helicases may be required to support a higher transcriptional and RNA metabolic activity of cancer cells because of their role in transcription, RNA processing, and export. However, DHX9 and other DEAD/H helicases also may be upregulated in cancer to prevent R-loop accumulation or to promote the restart of replication forks stalled by the excess of R-loops. Future work will determine whether these helicases play a role in oncogenesis, tumor progression, or drug resistance by promoting R-loop processing.

In conclusion, our study provides the first proteomic characterization of factors interacting with RNA/DNA hybrids *in vivo*, offering a new perspective on cellular R-loop functions, including transcriptional termination and maintenance of genome stability (Figure 7D). Thus, the RNA/DNA hybrid interactome constitutes a powerful resource to study R-loop biology in health and disease.

EXPERIMENTAL PROCEDURES

Cell Culture, Transfections, and Drug Treatments

HeLa and HEK293T cells were grown as described (Skourti-Stathaki et al., 2011). Cells were treated with 5 μg/mL actinomycin D for 6 hr, 10 μM CPT for the indicated time, 100 μM DRB for 3 hr, 50 μM cordycepin for 4 hr, and 10 μM or 0.1 μM Olaparib for 1 hr. Cell transfections are described in Supplemental Experimental Procedures.

ChIP and DIP Analysis

ChIP with 2–5 μg of the indicated antibodies was carried out as described (Groh et al., 2014). DIP analysis with the S9.6 antibody (Boguslawski et al., 1986) was described by Groh et al. (2014) and Skourti-Stathaki et al. (2011). RNase H digestion with 1.5 U RNase H (M0297, NEB) per microgram genomic DNA for 2.5 hr at 37°C was carried out before IP. The amount of immunoprecipitated material at a particular gene region was calculated as the percentage of input after subtracting the background signal (no antibody control). Where stated, the values were normalized to the indicated probes.

RNA/DNA Hybrid IP

Non-crosslinked HeLa cells were lysed in 85 mM KCl, 5 mM PIPES (pH 8.0), and 0.5% NP-40 for 10 min on ice. Pelleted nuclei were resuspended in RSB buffer (10 mM Tris-HCl pH 7.5, 200 mM NaCl, 2.5 mM MgCl2) with 0.2% sodium deoxycholate [NaDOC], 0.1% SDS, 0.05% sodium lauroyl sarcosinate [Na sarkosyl] and 0.5% Triton X-100, and extracts were sonicated for 10 min (Diagenode Bioruptor). Extracts were then diluted 1:4 in RSB with 0.5% Triton X-100 (RSB + T) and subjected to IP with the S9.6 antibody, bound to protein A dynabeads (Invitrogen), and preblocked with 0.5% BSA/PBS for 2 hr. CBP80 and IgG2a antibodies were used as control. RNase A (PureLink, Invitrogen) was added during IP at 0.1 ng RNase A per microgram genomic DNA. Beads were washed 4x with RSB + T; 2x with RSB; and eluted either in 2x LDS (Invitrogen), 100 mM DTT for 10 min at 70°C (for SDS-PAGE), or 1% SDS and 0.1 M $NaHCO_3$ for 30 min at room temperature (for RNA/DNA hybrid slot blot). Where indicated, nuclear extracts were treated with 1 U/μL benzonase (Sigma) for 30 min at 37°C before IP. Sequences and preparation of double-stranded competitors were described by Phillips et al. (2013) and Rigby et al. (2014). For MS analysis, eluted samples were processed by filter-aided sample preparation (FASP) with trypsin (Wiśniewski et al., 2009). Table S1 provides the list of proteins that make up the RNA/DNA interactome.

Statistical Analysis

Unless otherwise stated, values represent the means ± SEMs based on at least three independent experiments. Asterisks indicate statistical significance (*$p < 0.05$; **$p < 0.01$; ***$p < 0.001$), based on unpaired, two-tailed Student's t test. Correlations were calculated using Pearson's correlation coefficient (*r*).

DATA AND SOFTWARE AVAILABILITY

The accession number for the RNA/DNA interactome reported in this paper is PRIDE: PXD002960 (http://www.ebi.ac.uk/pride/archive/login).

SUPPLEMENTAL INFORMATION

Supplemental Information includes Supplemental Experimental Procedures, seven figures, and three tables and can be found with this article online at https://doi.org/10.1016/j.celrep.2018.04.025.

ACKNOWLEDGMENTS

We thank B. Thomas, S. Mohammed, and S. Hester from the Advanced Proteomics facility of the University of Oxford for their help with MS. We thank S. Irmscher for help with western blots and I. Gibbs-Seymour, L. Palazzo, and I. Ahel for PARP-related reagents. We thank N. Proudfoot for critically reading the manuscript and A. Castello, C. Norbury, and S. Murphy for helpful discussions. M.G. is supported by an Ataxia UK and Motor Neuron Disease Association studentship (Gromak/Jun11/6278) and funding from the Dunn School of Pathology (University of Oxford). M.S.K. is supported by the Lundbeck Foundation (R126-2012-12263). The Gromak laboratory is supported by a Royal Society University Research Fellowship (UF150656), an MRC New Investigator Research Grant (MR/J007870/1), and a John Fell award (BVD07340, Ref 133/090) to N.G.

AUTHOR CONTRIBUTIONS

M.G. designed and performed the experiments in Figures 1, 2, 4 A–4D 5A, 5D, and 5E, 6, and 7. A.C. designed and performed the experiments in Figures 2F, 3, 5B, 5C, 5F, and 5G, and 7B. M.S.K. performed experiments and analyzed the data in Figures 4E–4I. M.G., A.C., and N.G. analyzed the data and wrote the manuscript. N.G. conceived the study and supervised the project.

DECLARATION OF INTERESTS

The authors declare no competing interests.

Received: August 15, 2017
Revised: March 3, 2018
Accepted: April 4, 2018
Published: May 8, 2018

REFERENCES

Aguilera, A., and García-Muse, T. (2012). R loops: from transcription byproducts to threats to genome stability. Mol. Cell *46*, 115–124.

Anderson, S.F., Schlegel, B.P., Nakajima, T., Wolpin, E.S., and Parvin, J.D. (1998). BRCA1 protein is linked to the RNA polymerase II holoenzyme complex via RNA helicase A. Nat. Genet. *19*, 254–256.

Bhatia, V., Barroso, S.I., Garcia-Rubio, M.L., Tumini, E., Herrera-Moyano, E., and Aguilera, A. (2014). BRCA2 prevents R-loop accumulation and associates with TREX-2 mRNA export factor PCID2. Nature *511*, 362–365.

Boguslawski, S.J., Smith, D.E., Michalak, M.A., Mickelson, K.E., Yehle, C.O., Patterson, W.L., and Carrico, R.J. (1986). Characterization of monoclonal antibody to DNA.RNA and its application to immunodetection of hybrids. J. Immunol. Methods *89*, 123–130.

Boque-Sastre, R., Soler, M., Oliveira-Mateos, C., Portela, A., Moutinho, C., Sayols, S., Villanueva, A., Esteller, M., and Guil, S. (2015). Head-to-head antisense transcription and R-loop formation promotes transcriptional activation. Proc. Natl. Acad. Sci. USA *112*, 5785–5790.

Britton, S., Dernoncourt, E., Delteil, C., Froment, C., Schiltz, O., Salles, B., Frit, P., and Calsou, P. (2014). DNA damage triggers SAF-A and RNA biogenesis factors exclusion from chromatin coupled to R-loops removal. Nucleic Acids Res. *42*, 9047–9062.

Castello, A., Fischer, B., Eichelbaum, K., Horos, R., Beckmann, B.M., Strein, C., Davey, N.E., Humphreys, D.T., Preiss, T., Steinmetz, L.M., et al. (2012). Insights into RNA biology from an atlas of mammalian mRNA-binding proteins. Cell *149*, 1393–1406.

Cerritelli, S.M., and Crouch, R.J. (2009). Ribonuclease H: the enzymes in eukaryotes. FEBS J. *276*, 1494–1505.

Chakraborty, P., and Grosse, F. (2011). Human DHX9 helicase preferentially unwinds RNA-containing displacement loops (R-loops) and G-quadruplexes. DNA Repair (Amst.) *10*, 654–665.

Chan, Y.A., Aristizabal, M.J., Lu, P.Y., Luo, Z., Hamza, A., Kobor, M.S., Stirling, P.C., and Hieter, P. (2014). Genome-wide profiling of yeast DNA:RNA hybrid prone sites with DRIP-chip. PLoS Genet. *10*, e1004288.

Chen, Z.X., Wallis, K., Fell, S.M., Sobrado, V.R., Hemmer, M.C., Ramsköld, D., Hellman, U., Sandberg, R., Kenchappa, R.S., Martinson, T., et al. (2014). RNA helicase A is a downstream mediator of KIF1Bβ tumor-suppressor function in neuroblastoma. Cancer Discov. *4*, 434–451.

Cohen, S., Puget, N., Lin, Y.L., Clouaire, T., Aguirrebengoa, M., Rocher, V., Pasero, P., Canitrot, Y., and Legube, G. (2018). Senataxin resolves RNA:DNA hybrids forming at DNA double-strand breaks to prevent translocations. Nat. Commun. *9*, 533.

Colak, D., Zaninovic, N., Cohen, M.S., Rosenwaks, Z., Yang, W.Y., Gerhardt, J., Disney, M.D., and Jaffrey, S.R. (2014). Promoter-bound trinucleotide repeat mRNA drives epigenetic silencing in fragile X syndrome. Science *343*, 1002–1005.

Cox, J., and Mann, M. (2008). MaxQuant enables high peptide identification rates, individualized p.p.b.-range mass accuracies and proteome-wide protein quantification. Nat. Biotechnol. *26*, 1367–1372.

Cristini, A., Park, J.H., Capranico, G., Legube, G., Favre, G., and Sordet, O. (2016). DNA-PK triggers histone ubiquitination and signaling in response to DNA double-strand breaks produced during the repair of transcription-blocking topoisomerase I lesions. Nucleic Acids Res. *44*, 1161–1178.

Das, B.B., Huang, S.Y., Murai, J., Rehman, I., Amé, J.C., Sengupta, S., Das, S.K., Majumdar, P., Zhang, H., Biard, D., et al. (2014). PARP1-TDP1 coupling for the repair of topoisomerase I-induced DNA damage. Nucleic Acids Res. *42*, 4435–4449.

El Hage, A., French, S.L., Beyer, A.L., and Tollervey, D. (2010). Loss of topoisomerase I leads to R-loop-mediated transcriptional blocks during ribosomal RNA synthesis. Genes Dev. *24*, 1546–1558.

Forbes, S.A., Beare, D., Gunasekaran, P., Leung, K., Bindal, N., Boutselakis, H., Ding, M., Bamford, S., Cole, C., Ward, S., et al. (2015). COSMIC: exploring the world's knowledge of somatic mutations in human cancer. Nucleic Acids Res. *43*, D805–D811.

Gagné, J.P., Pic, E., Isabelle, M., Krietsch, J., Ethier, C., Paquet, E., Kelly, I., Boutin, M., Moon, K.M., Foster, L.J., and Poirier, G.G. (2012). Quantitative proteomics profiling of the poly(ADP-ribose)-related response to genotoxic stress. Nucleic Acids Res. *40*, 7788–7805.

Gavaldá, S., Gallardo, M., Luna, R., and Aguilera, A. (2013). R-loop mediated transcription-associated recombination in trf4Δ mutants reveals new links between RNA surveillance and genome integrity. PLoS One *8*, e65541.

Geiger, T., Wehner, A., Schaab, C., Cox, J., and Mann, M. (2012). Comparative proteomic analysis of eleven common cell lines reveals ubiquitous but varying expression of most proteins. Mol. Cell. Proteomics *11*, M111.014050.

Ginno, P.A., Lott, P.L., Christensen, H.C., Korf, I., and Chédin, F. (2012). R-loop formation is a distinctive characteristic of unmethylated human CpG island promoters. Mol. Cell *45*, 814–825.

Gómez-González, B., García-Rubio, M., Bermejo, R., Gaillard, H., Shirahige, K., Marín, A., Foiani, M., and Aguilera, A. (2011). Genome-wide function of THO/TREX in active genes prevents R-loop-dependent replication obstacles. EMBO J. *30*, 3106–3119.

Groh, M., and Gromak, N. (2014). Out of balance: R-loops in human disease. PLoS Genet. *10*, e1004630.

Groh, M., Lufino, M.M., Wade-Martins, R., and Gromak, N. (2014). R-loops associated with triplet repeat expansions promote gene silencing in Friedreich ataxia and fragile X syndrome. PLoS Genet. *10*, e1004318.

Groh, M., Albulescu, L.O., Cristini, A., and Gromak, N. (2017). Senataxin: genome guardian at the interface of transcription and neurodegeneration. J. Mol. Biol. *429*, 3181–3195.

Haeusler, A.R., Donnelly, C.J., Periz, G., Simko, E.A., Shaw, P.G., Kim, M.S., Maragakis, N.J., Troncoso, J.C., Pandey, A., Sattler, R., et al. (2014). C9orf72 nucleotide repeat structures initiate molecular cascades of disease. Nature *507*, 195–200.

Hatchi, E., Skourti-Stathaki, K., Ventz, S., Pinello, L., Yen, A., Kamieniarz-Gdula, K., Dimitrov, S., Pathania, S., McKinney, K.M., Eaton, M.L., et al. (2015). BRCA1 recruitment to transcriptional pause sites is required for R-loop-driven DNA damage repair. Mol. Cell *57*, 636–647.

Hegele, A., Kamburov, A., Grossmann, A., Sourlis, C., Wowro, S., Weimann, M., Will, C.L., Pena, V., Lührmann, R., and Stelzl, U. (2012). Dynamic protein-protein interaction wiring of the human spliceosome. Mol. Cell *45*, 567–580.

Herrera-Moyano, E., Mergui, X., García-Rubio, M.L., Barroso, S., and Aguilera, A. (2014). The yeast and human FACT chromatin-reorganizing complexes solve R-loop-mediated transcription-replication conflicts. Genes Dev. *28*, 735–748.

Hudson, W.H., and Ortlund, E.A. (2014). The structure, function and evolution of proteins that bind DNA and RNA. Nat. Rev. Mol. Cell Biol. *15*, 749–760.

Isabelle, M., Moreel, X., Gagné, J.P., Rouleau, M., Ethier, C., Gagné, P., Hendzel, M.J., and Poirier, G.G. (2010). Investigation of PARP-1, PARP-2, and

PARG interactomes by affinity-purification mass spectrometry. Proteome Sci. *8*, 22.

Khobta, A., Ferri, F., Lotito, L., Montecucco, A., Rossi, R., and Capranico, G. (2006). Early effects of topoisomerase I inhibition on RNA polymerase II along transcribed genes in human cells. J. Mol. Biol. *357*, 127–138.

Kotsantis, P., Silva, L.M., Irmscher, S., Jones, R.M., Folkes, L., Gromak, N., and Petermann, E. (2016). Increased global transcription activity as a mechanism of replication stress in cancer. Nat. Commun. *7*, 13087.

Kustatscher, G., Wills, K.L., Furlan, C., and Rappsilber, J. (2014). Chromatin enrichment for proteomics. Nat. Protoc. *9*, 2090–2099.

Lee, T., and Pelletier, J. (2016). The biology of DHX9 and its potential as a therapeutic target. Oncotarget *7*, 42716–42739.

Li, X., and Manley, J.L. (2005). Inactivation of the SR protein splicing factor ASF/SF2 results in genomic instability. Cell *122*, 365–378.

Liu, M., Roth, A., Yu, M., Morris, R., Bersani, F., Rivera, M.N., Lu, J., Shioda, T., Vasudevan, S., Ramaswamy, S., et al. (2013). The IGF2 intronic miR-483 selectively enhances transcription from IGF2 fetal promoters and enhances tumorigenesis. Genes Dev. *27*, 2543–2548.

Marinello, J., Bertoncini, S., Aloisi, I., Cristini, A., Malagoli Tagliazucchi, G., Forcato, M., Sordet, O., and Capranico, G. (2016). Dynamic effects of topoisomerase I inhibition on R-loops and short transcripts at active promoters. PLoS One *11*, e0147053.

Mischo, H.E., Hemmerich, P., Grosse, F., and Zhang, S. (2005). Actinomycin D induces histone gamma-H2AX foci and complex formation of gamma-H2AX with Ku70 and nuclear DNA helicase II. J. Biol. Chem. *280*, 9586–9594.

Morales, J.C., Richard, P., Patidar, P.L., Motea, E.A., Dang, T.T., Manley, J.L., and Boothman, D.A. (2016). XRN2 links transcription termination to DNA damage and replication stress. PLoS Genet. *12*, e1006107.

Myöhänen, S., and Baylin, S.B. (2001). Sequence-specific DNA binding activity of RNA helicase A to the p16INK4a promoter. J. Biol. Chem. *276*, 1634–1642.

Nakajima, T., Uchida, C., Anderson, S.F., Lee, C.G., Hurwitz, J., Parvin, J.D., and Montminy, M. (1997). RNA helicase A mediates association of CBP with RNA polymerase II. Cell *90*, 1107–1112.

Paulsen, R.D., Soni, D.V., Wollman, R., Hahn, A.T., Yee, M.C., Guan, A., Hesley, J.A., Miller, S.C., Cromwell, E.F., Solow-Cordero, D.E., et al. (2009). A genome-wide siRNA screen reveals diverse cellular processes and pathways that mediate genome stability. Mol. Cell *35*, 228–239.

Pellizzoni, L., Baccon, J., Charroux, B., and Dreyfuss, G. (2001). The survival of motor neurons (SMN) protein interacts with the snoRNP proteins fibrillarin and GAR1. Curr. Biol. *11*, 1079–1088.

Phillips, D.D., Garboczi, D.N., Singh, K., Hu, Z., Leppla, S.H., and Leysath, C.E. (2013). The sub-nanomolar binding of DNA-RNA hybrids by the single-chain Fv fragment of antibody S9.6. J. Mol. Recognit. *26*, 376–381.

Rhodes, D.R., Kalyana-Sundaram, S., Mahavisno, V., Varambally, R., Yu, J., Briggs, B.B., Barrette, T.R., Anstet, M.J., Kincead-Beal, C., Kulkarni, P., et al. (2007). Oncomine 3.0: genes, pathways, and networks in a collection of 18,000 cancer gene expression profiles. Neoplasia *9*, 166–180.

Rigby, R.E., Webb, L.M., Mackenzie, K.J., Li, Y., Leitch, A., Reijns, M.A., Lundie, R.J., Revuelta, A., Davidson, D.J., Diebold, S., et al. (2014). RNA:DNA hybrids are a novel molecular pattern sensed by TLR9. EMBO J. *33*, 542–558.

Schwab, R.A., Nieminuszczy, J., Shah, F., Langton, J., Lopez Martinez, D., Liang, C.C., Cohn, M.A., Gibbons, R.J., Deans, A.J., and Niedzwiedz, W. (2015). The Fanconi anemia pathway maintains genome stability by coordinating replication and transcription. Mol. Cell *60*, 351–361.

Skourti-Stathaki, K., and Proudfoot, N.J. (2014). A double-edged sword: R loops as threats to genome integrity and powerful regulators of gene expression. Genes Dev. *28*, 1384–1396.

Skourti-Stathaki, K., Proudfoot, N.J., and Gromak, N. (2011). Human senataxin resolves RNA/DNA hybrids formed at transcriptional pause sites to promote Xrn2-dependent termination. Mol. Cell *42*, 794–805.

Skourti-Stathaki, K., Kamieniarz-Gdula, K., and Proudfoot, N.J. (2014). R-loops induce repressive chromatin marks over mammalian gene terminators. Nature *516*, 436–439.

Smyth, G.K. (2004). Linear models and empirical bayes methods for assessing differential expression in microarray experiments. Stat. Appl. Genet. Mol. Biol. *3*, Article3.

Sollier, J., and Cimprich, K.A. (2015). Breaking bad: R-loops and genome integrity. Trends Cell Biol. *25*, 514–522.

Sordet, O., Redon, C.E., Guirouilh-Barbat, J., Smith, S., Solier, S., Douarre, C., Conti, C., Nakamura, A.J., Das, B.B., Nicolas, E., et al. (2009). Ataxia telangiectasia mutated activation by transcription- and topoisomerase I-induced DNA double-strand breaks. EMBO Rep. *10*, 887–893.

Stirling, P.C., Chan, Y.A., Minaker, S.W., Aristizabal, M.J., Barrett, I., Sipahimalani, P., Kobor, M.S., and Hieter, P. (2012). R-loop-mediated genome instability in mRNA cleavage and polyadenylation mutants. Genes Dev. *26*, 163–175.

Stork, C.T., Bocek, M., Crossley, M.P., Sollier, J., Sanz, L.A., Chédin, F., Swigut, T., and Cimprich, K.A. (2016). Co-transcriptional R-loops are the main cause of estrogen-induced DNA damage. eLife *5*, e17548.

Tallis, M., Morra, R., Barkauskaite, E., and Ahel, I. (2014). Poly(ADP-ribosyl) ation in regulation of chromatin structure and the DNA damage response. Chromosoma *123*, 79–90.

Trudgian, D.C., Ridlova, G., Fischer, R., Mackeen, M.M., Ternette, N., Acuto, O., Kessler, B.M., and Thomas, B. (2011). Comparative evaluation of label-free SINQ normalized spectral index quantitation in the central proteomics facilities pipeline. Proteomics *11*, 2790–2797.

Tuduri, S., Crabbé, L., Conti, C., Tourrière, H., Holtgreve-Grez, H., Jauch, A., Pantesco, V., De Vos, J., Thomas, A., Theillet, C., et al. (2009). Topoisomerase I suppresses genomic instability by preventing interference between replication and transcription. Nat. Cell Biol. *11*, 1315–1324.

von Kobbe, C., Harrigan, J.A., Schreiber, V., Stiegler, P., Piotrowski, J., Dawut, L., and Bohr, V.A. (2004). Poly(ADP-ribose) polymerase 1 regulates both the exonuclease and helicase activities of the Werner syndrome protein. Nucleic Acids Res. *32*, 4003–4014.

Wahba, L., Amon, J.D., Koshland, D., and Vuica-Ross, M. (2011). RNase H and multiple RNA biogenesis factors cooperate to prevent RNA:DNA hybrids from generating genome instability. Mol. Cell *44*, 978–988.

Wiśniewski, J.R., Zougman, A., Nagaraj, N., and Mann, M. (2009). Universal sample preparation method for proteome analysis. Nat. Methods *6*, 359–362.

Yang, Y., McBride, K.M., Hensley, S., Lu, Y., Chedin, F., and Bedford, M.T. (2014). Arginine methylation facilitates the recruitment of TOP3B to chromatin to prevent R loop accumulation. Mol. Cell *53*, 484–497.

Zhou, K., Choe, K.T., Zaidi, Z., Wang, Q., Mathews, M.B., and Lee, C.G. (2003). RNA helicase A interacts with dsDNA and topoisomerase IIalpha. Nucleic Acids Res. *31*, 2253–2260.

Cell Reports, Volume *23*

Supplemental Information

RNA/DNA Hybrid Interactome Identifies DXH9 as a Molecular Player in Transcriptional Termination and R-Loop-Associated DNA Damage

Agnese Cristini, Matthias Groh, Maiken S. Kristiansen, and Natalia Gromak

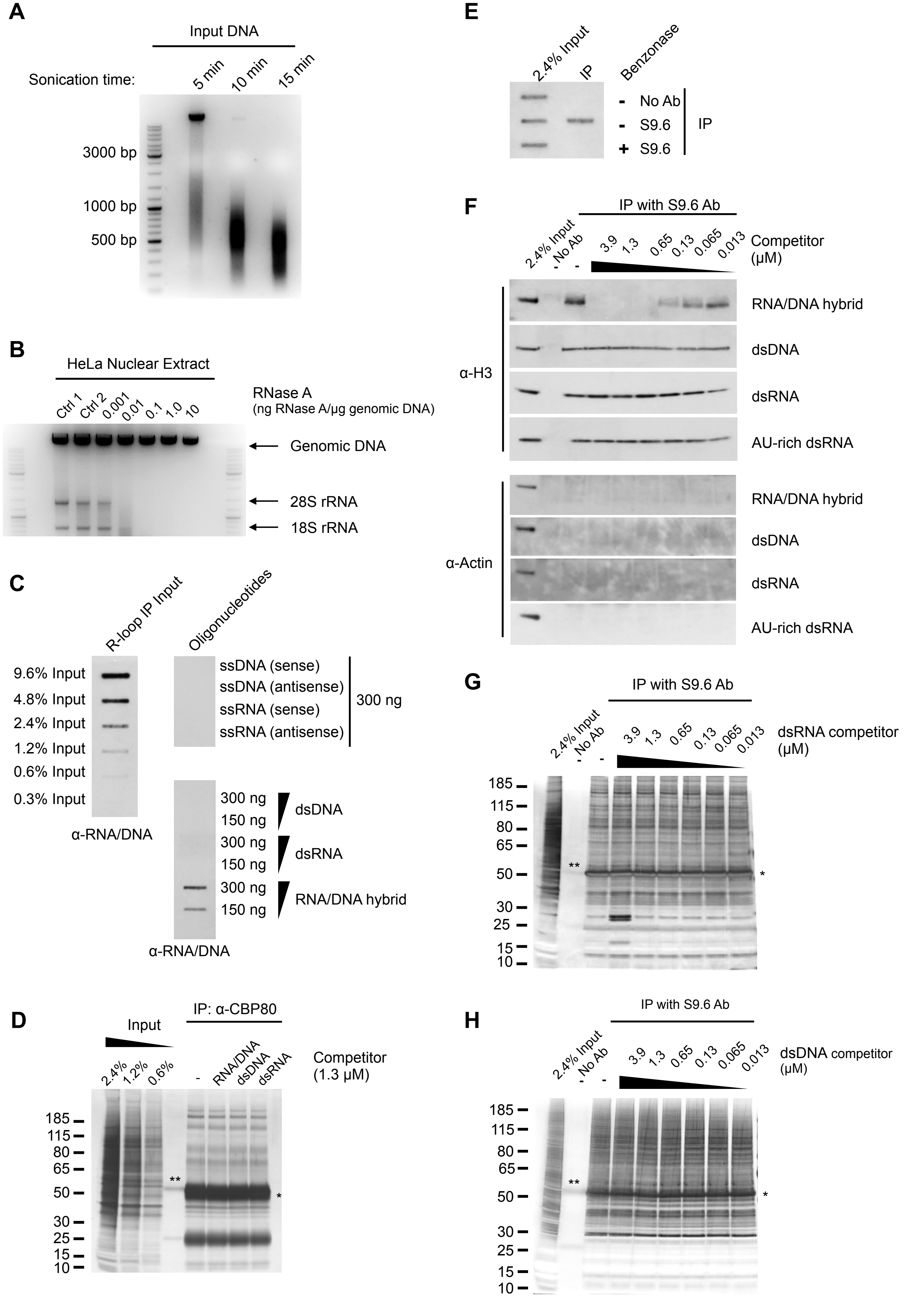
A
Input DNA
Sonication time:
5 min
10 min
15 min
3000 bp
1000 bp
500 bp
B
HeLa Nuclear Extract
Ctrl 1
Ctrl 2
0.001
0.01
0.1
1.0
10
RNase A
(ng RNase A/µg genomic DNA)
Genomic DNA
28S rRNA
18S rRNA
C
R-loop IP Input
9.6% Input
4.8% Input
2.4% Input
1.2% Input
0.6% Input
0.3% Input
α-RNA/DNA
Oligonucleotides
ssDNA (sense)
ssDNA (antisense)
ssRNA (sense)
ssRNA (antisense)
300 ng
300 ng
150 ng
dsDNA
300 ng
150 ng
dsRNA
300 ng
150 ng
RNA/DNA hybrid
α-RNA/DNA
D
Input
2.4%
1.2%
0.6%
IP: α-CBP80
-
RNA/DNA
dsDNA
dsRNA
Competitor
(1.3 µM)
185
115
80
65
50
30
25
15
10
**
*
E
2.4% Input
IP
Benzonase
- No Ab
- S9.6
+ S9.6
IP
F
IP with S9.6 Ab
2.4% Input
No Ab
-
-
3.9
1.3
0.65
0.13
0.065
0.013
Competitor
(µM)
α-H3
RNA/DNA hybrid
dsDNA
dsRNA
AU-rich dsRNA
α-Actin
RNA/DNA hybrid
dsDNA
dsRNA
AU-rich dsRNA
G
IP with S9.6 Ab
2.4% Input
No Ab
-
-
3.9
1.3
0.65
0.13
0.065
0.013
dsRNA competitor
(µM)
185
115
80
65
50
30
25
15
10
**
*
H
IP with S9.6 Ab
2.4% Input
No Ab
-
-
3.9
1.3
0.65
0.13
0.065
0.013
dsDNA competitor
(µM)
185
115
80
65
50
30
25
15
10
**
*

Figure S1. Design and validation of RNA/DNA hybrid IP method. Related to Figure 1

A. Agarose gel analysis of DNA fragment sizes from RNA/DNA hybrid IP samples, sonicated for 5, 10 and 15 min. DNA size markers are shown to the left of the gel.

B. Agarose gel analysis of RNA trimming by RNase A during RNA/DNA hybrid IP. 5 µg of purified genomic DNA, prepared according to the RNA/DNA hybrid IP protocol, was treated with RNase A for 2h at 37°C. Control samples (Ctrl 1 and Ctrl 2) were not treated with RNase A. The migration of genomic DNA and ribosomal RNA (18S and 28S rRNA) is indicated on the right of the gel.

C. S9.6 antibody specifically recognises endogenous and synthetic RNA/DNA hybrids at a wide range of concentrations. Slot blot analysis with S9.6 antibody. Different amounts of endogenous and synthetic RNA/DNA hybrids were loaded on the slot blot. 300 ng of indicated synthetic single-stranded oligonucleotides were used as negative controls (right top panel).

D. Silver-stain of CBP80 IP with nucleic acid competitors added at 1.3 uM.

E. RNA/DNA hybrid slot blot after benzonase treatment.

F. Western blot of RNA/DNA hybrid IP samples in the presence of indicated competitors added at 3.9-0.013 uM. Western blot was probed with histone H3 (top panel) and actin (bottom panel) antibodies.

G-H. Silver-stain of RNA/DNA hybrid IP with dsRNA (G) and dsDNA (H) competitors.

Asterisk (*) indicates the heavy chain from the S9.6 and IgG2a antibodies. Band labelled (**) in ‘No Ab’ lane corresponds to BSA, used to block protein A Dynabeads.

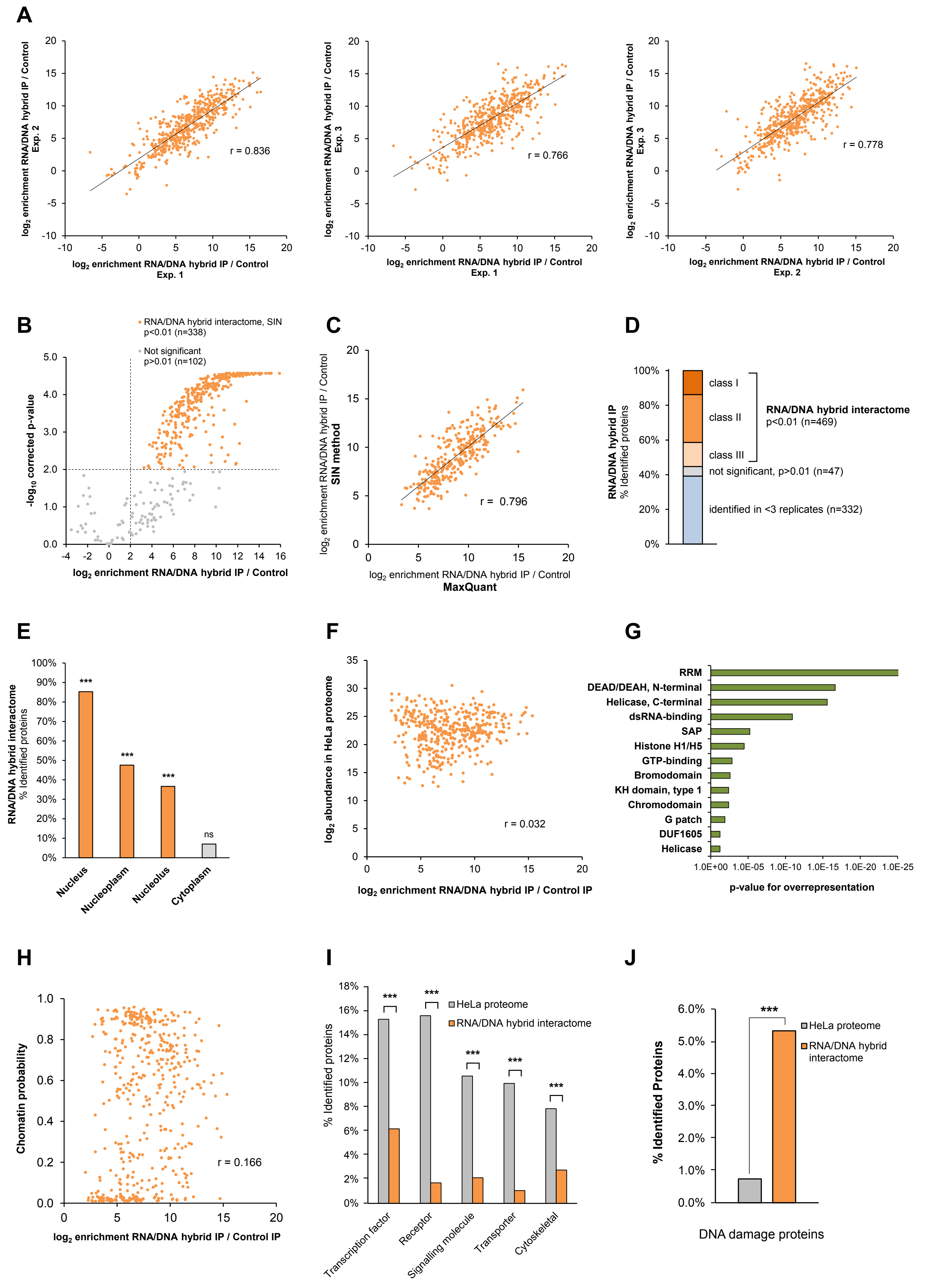

A
log2 enrichment RNA/DNA hybrid IP / Control Exp. 2
log2 enrichment RNA/DNA hybrid IP / Control Exp. 1
r = 0.836
log2 enrichment RNA/DNA hybrid IP / Control Exp. 3
r = 0.766
r = 0.778
log2 enrichment RNA/DNA hybrid IP / Control Exp. 2
B
RNA/DNA hybrid interactome, SIN p<0.01 (n=338)
Not significant p>0.01 (n=102)
-log10 corrected p-value
log2 enrichment RNA/DNA hybrid IP / Control
C
log2 enrichment RNA/DNA hybrid IP / Control SIN method
r = 0.796
log2 enrichment RNA/DNA hybrid IP / Control MaxQuant
D
RNA/DNA hybrid IP % Identified proteins
class I
class II
class III
RNA/DNA hybrid interactome p<0.01 (n=469)
not significant, p>0.01 (n=47)
identified in <3 replicates (n=332)
E
RNA/DNA hybrid interactome % Identified proteins
Nucleus
Nucleoplasm
Nucleolus
Cytoplasm
ns
F
log2 abundance in HeLa proteome
r = 0.032
log2 enrichment RNA/DNA hybrid IP / Control IP
G
RRM
DEAD/DEAH, N-terminal
Helicase, C-terminal
dsRNA-binding
SAP
Histone H1/H5
GTP-binding
Bromodomain
KH domain, type 1
Chromodomain
G patch
DUF1605
Helicase
1.0E+00 1.0E-05 1.0E-10 1.0E-15 1.0E-20 1.0E-25
p-value for overrepresentation
H
Chomatin probability
r = 0.166
log2 enrichment RNA/DNA hybrid IP / Control IP
I
% Identified proteins
HeLa proteome
RNA/DNA hybrid interactome
Transcription factor
Receptor
Signalling molecule
Transporter
Cytoskeletal
J
% Identified Proteins
HeLa proteome
RNA/DNA hybrid interactome
DNA damage proteins

Figure S2. **RNA/DNA hybrid interactome analysis. Related to Figure 2**

A. RNA/DNA hybrid IP is highly reproducible. The panel shows the correlation between $\log_2$ enrichment RNA/DNA hybrid IP / Control IP of proteins quantified in three independent biological replicates of RNA/DNA hybrid IP by mass spectrometry.

B. Volcano plot displaying mass spectrometry results of three biological replicates of RNA/DNA hybrid IP experiments, using normalised spectral indexes (SIN) quantification method, as implemented in the SINQ software (Trudgian et al., 2011). Averaged $\log_2$ ratios between RNA/DNA hybrid IP and control IP, carried out in the presence of 1.3 uM synthetic RNA/DNA hybrid, are plotted against their Benjamini-Hochberg corrected $-\log_{10}$ p-values calculated across all three biological replicates using a moderated t-test. Proteins (n=338) significantly enriched in R-loop IP/Control are plotted in orange. Dashed lines indicate the significance cutoffs ($\log_2$ enrichment > 2 and $-\log_{10} > 2$).

C. Correlation between protein enrichment in RNA/DNA hybrid IP/Control IP of proteins quantified by MaxQuant method and SIN method.

D. Classification of proteins identified in RNA/DNA hybrid IP mass spectrometry on the basis on moderated t-test of three biological replicates with p-value corrected according to Benjamini-Hochberg. 469 Proteins enriched in RNA/DNA hybrid IP (corrected p-value <0.01) represented the 'RNA/DNA hybrid interactome'. The RNA/DNA hybrid interactome was further subdivided into three classes according to the corrected p-values: top 25% (class I), middle 50% (class II), and bottom 25% (class III). 379 proteins were identified but not enriched (grey).

E. Cellular compartment analysis of RNA/DNA hybrid interactome ($p < 0.01$). Asterisks (***) indicate highly significant enrichment of the depicted compartments in RNA/DNA hybrid IP as determined by Fisher's exact test (Benjamini-Hochberg corrected p-values of $2x10^{-127}$, $1x10^{-69}$, $8x10^{-113}$, respectively).

F. Relative protein abundance in RNA/DNA hybrid interactome as compared to the total cellular protein abundance in HeLa cells (Geiger et al., 2012). Log_2 enrichment RNA/DNA hybrid IP/Control IP of proteins in RNA/DNA hybrid interactome (x-axis) is plotted against their corresponding Log_2 abundance in the total HeLa proteome (y-axis).

G. Protein domains overrepresented in RNA/DNA hybrid IP. Analysis of enrichment of Pfam InterPro Domains in RNA/DNA hybrid interactome using 'Enrichr' software (Chen et al., 2013). Top thirteen significantly overrepresented protein domains as determined by Fisher's exact test are shown and ranked according to their Benjamini-Hochberg corrected p-value.

H. Relative protein abundance in RNA/DNA hybrid interactome compared to chromatin probability (Kustatscher et al., 2014). Log_2 enrichment RNA/DNA hybrid IP/Control IP of proteins in RNA/DNA hybrid interactome (x-axis) is plotted against their corresponding chromatin probability (y-axis).

I-J. Enrichment analysis of abundant protein families (I) and factors mediating genome stability (Paulsen et al., 2009) (J) in the RNA/DNA hybrid interactome.

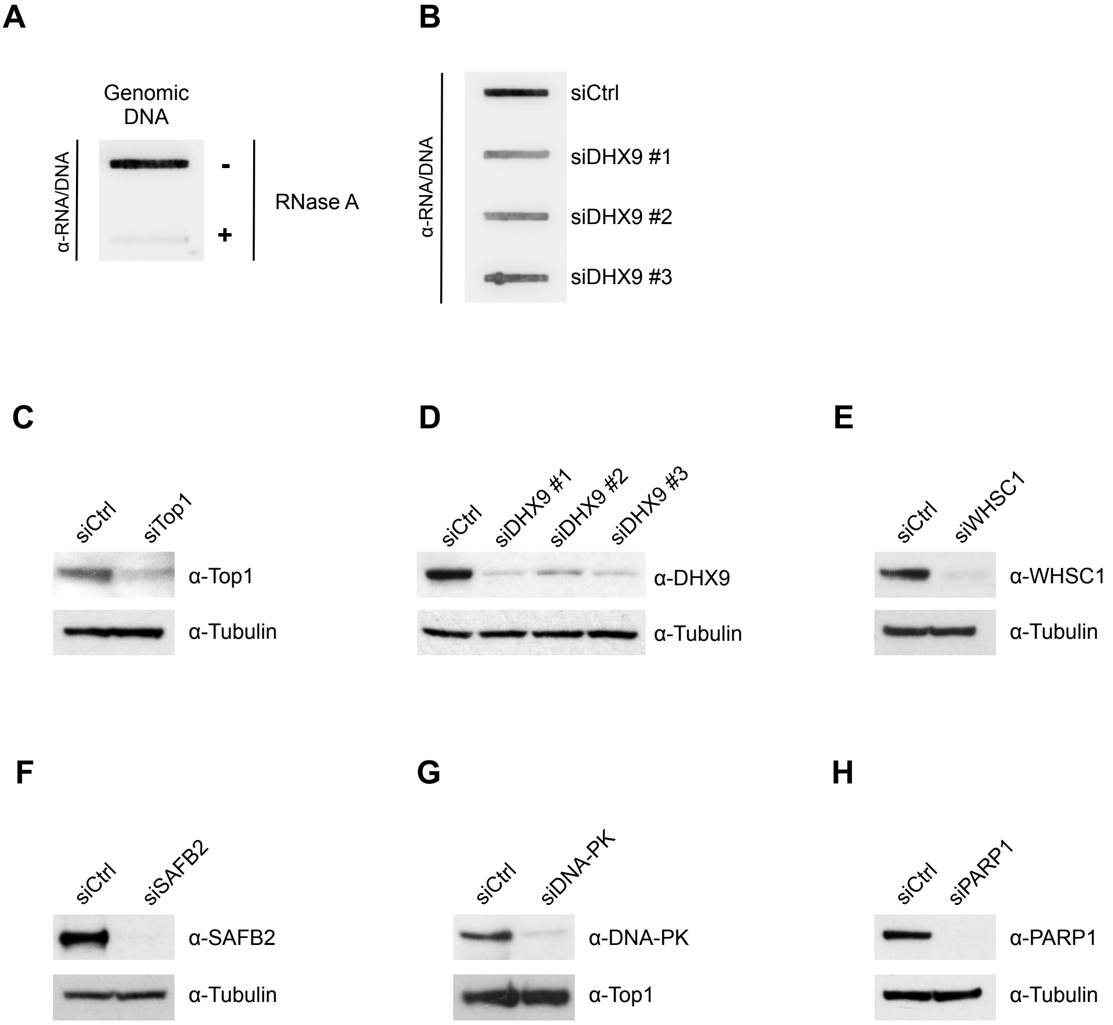

Figure S3. Validation of new RNA/DNA hybrid interactome candidates. Related to Figure 3

A. RNA/DNA hybrid slot blot of HeLa genomic DNA from nuclear extracts treated with 0.1 mg/ml RNase A for 1 h at 37°C and probed with S9.6 antibody.

B. RNA/DNA hybrid slot blot of genomic DNA from HeLa cells transfected with control (siCtrl) or the indicated siRNA targeting DHX9.

C-H. Western blot of whole cell extracts from HeLa cells transfected with control (siCtrl) or indicated siRNAs sequences. Blots were probed with the indicated antibodies. Tubulin and Top1 were used as loading controls.

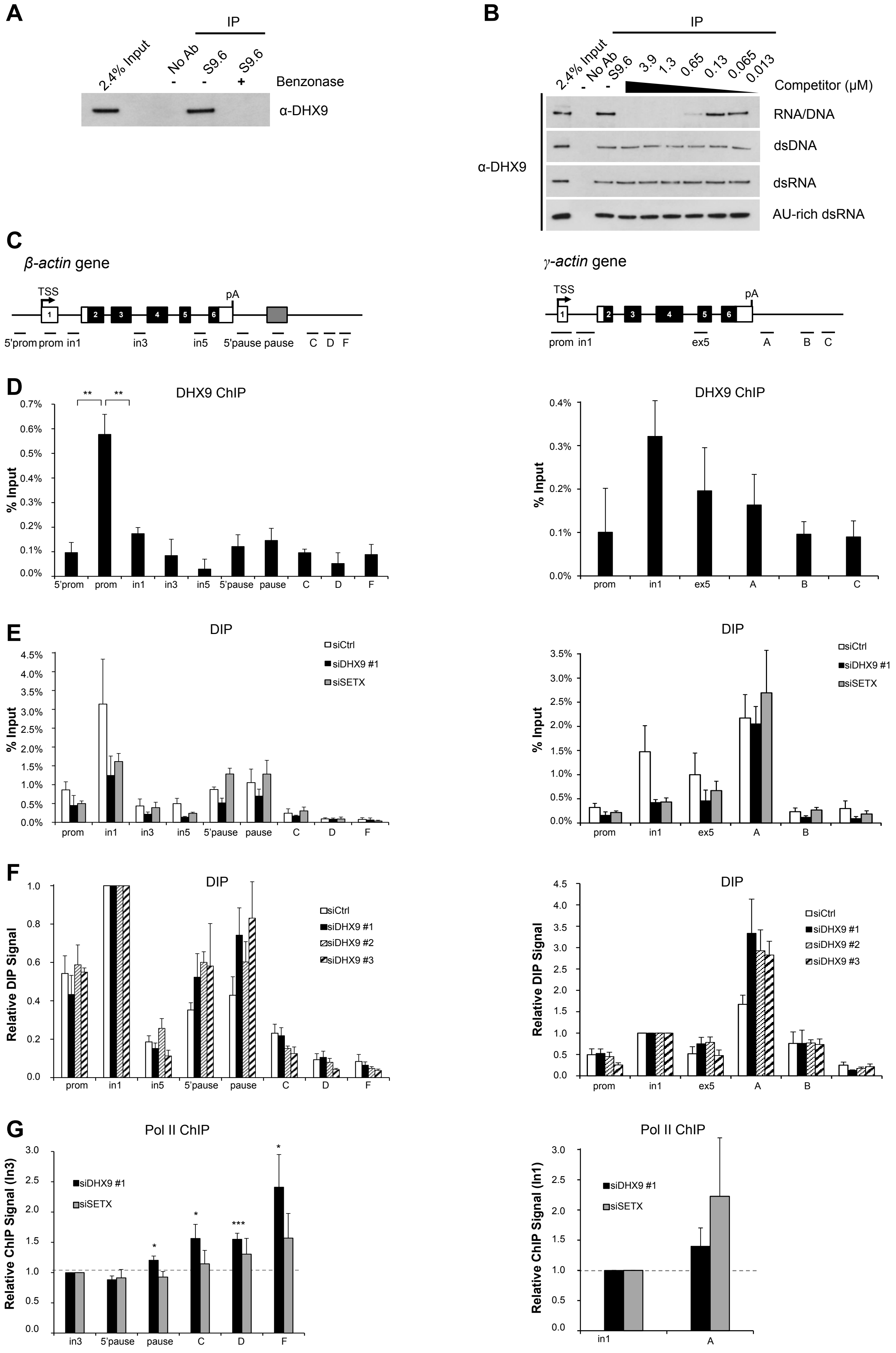
A
IP
2.4% Input
No Ab
S9.6
S9.6
- - +
Benzonase
α-DHX9
B
IP
2.4% Input
No Ab
S9.6
3.9
1.3
0.65
0.13
0.065
0.013
Competitor (μM)
α-DHX9
RNA/DNA
dsDNA
dsRNA
AU-rich dsRNA
C
β-actin gene
TSS
pA
5'prom prom in1 in3 in5 5'pause pause C D F
γ-actin gene
TSS
pA
prom in1 ex5 A B C
D
DHX9 ChIP
% Input
5'prom prom in1 in3 in5 5'pause pause C D F
DHX9 ChIP
% Input
prom in1 ex5 A B C
E
DIP
siCtrl
siDHX9 #1
siSETX
% Input
prom in1 in3 in5 5'pause pause C D F
DIP
siCtrl
siDHX9 #1
siSETX
% Input
prom in1 ex5 A B
F
DIP
siCtrl
siDHX9 #1
siDHX9 #2
siDHX9 #3
Relative DIP Signal
prom in1 in5 5'pause pause C D F
DIP
siCtrl
siDHX9 #1
siDHX9 #2
siDHX9 #3
Relative DIP Signal
prom in1 ex5 A B
G
Pol II ChIP
siDHX9 #1
siSETX
Relative ChIP Signal (In3)
in3 5'pause pause C D F
Pol II ChIP
siDHX9 #1
siSETX
Relative ChIP Signal (In1)
in1 A

Figure S4. DHX9 binds R-loops *in vivo* and promotes transcription termination. Related to Figure 4

A. Western blot of RNA/DNA hybrids IP samples. Nuclear extracts were treated with benzonase (1 U/ul) for 30 min prior to IP with S9.6 antibody. Western blot was probed with DHX9 antibody.

B. Western blot of DHX9 in RNA/DNA hybrid IP in presence of the indicated synthetic competitors.

C. Diagram of *β-actin* (left panel) and *γ-actin* (right panel) genes. Exons are black, UTRs are white, TSS is transcriptional start site. qPCR amplicons are shown below the diagram. Grey box denotes a termination region.

D. DHX9 ChIP in HeLa cells on *β-actin* (left panel) and *γ-actin* (right panel) genes. Values are % of Input.

E. DIP in HeLa cells, transfected with control (siCtrl), DHX9 #1 and SETX siRNAs, on *β-actin* (left panel) and *γ-actin* (right panel) genes. Values are percentage of input from Figure 4H.

F. DIP in HeLa cells, transfected with the indicated siRNAs targeting DHX9 or with a control sequence (siCtrl), on *β-actin* (left panel) and *γ-actin* (right panel) genes. Values are relative to in1.

G. Pol II ChIP in HeLa cells, transfected with control (siCtrl), DHX9 #1 and SETX siRNAs, on *β-actin* (left panel) and *γ-actin* (right panel) genes. Values are normalized to *β-actin* in 3 and *γ-actin* in 1, respectively.

Bars in D-G represent the average values from at least three independent experiments +/- SEM with * $p<0.05$, ** $p<0.01$, *** $p<0.001$ (unpaired, two-tailed Student's t test).

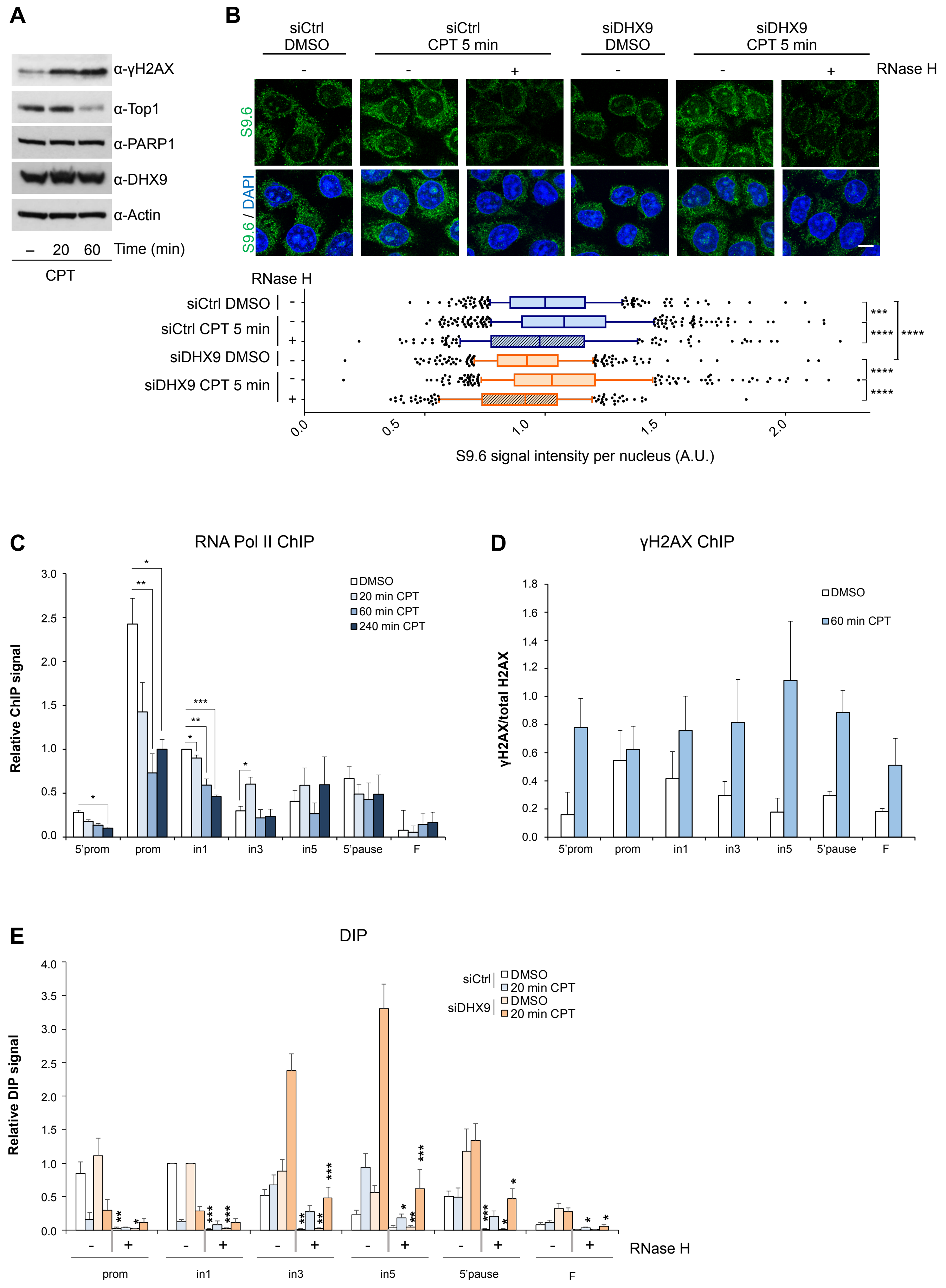

A
α-γH2AX
α-Top1
α-PARP1
α-DHX9
α-Actin
– 20 60 Time (min)
CPT
B
siCtrl DMSO
siCtrl CPT 5 min
siDHX9 DMSO
siDHX9 CPT 5 min
RNase H
S9.6
S9.6 / DAPI
siCtrl DMSO
siCtrl CPT 5 min
siDHX9 DMSO
siDHX9 CPT 5 min
S9.6 signal intensity per nucleus (A.U.)
C
RNA Pol II ChIP
Relative ChIP signal
DMSO
20 min CPT
60 min CPT
240 min CPT
5'prom
prom
in1
in3
in5
5'pause
F
D
γH2AX ChIP
γH2AX/total H2AX
DMSO
60 min CPT
5'prom
prom
in1
in3
in5
5'pause
F
E
DIP
Relative DIP signal
siCtrl
DMSO
20 min CPT
siDHX9
DMSO
20 min CPT
RNase H
prom
in1
in3
in5
5'pause
F

Figure S5. DNA damage and R-loop induction upon CPT treatment. Related to Figure 5

A. Western blot of total HeLa extracts, treated with DMSO or CPT for the indicated times, probed with indicated antibodies. Actin was used as loading control.

B. IF analysis of R-loops using S9.6 antibody (green) in Hela cells transfected with DHX9 #1 or control siRNA and treated with DMSO or CPT for 5 min. DAPI (blue) depicts the nuclei. Cells were treated (+) or left untreated (-) with RNase H prior to S9.6 staining. Top panel: representative images. Bar: 10 um. Bottom panel: S9.6 intensity per nucleus. More than 100 nuclei were analyzed per condition (n=3, apart from RNase H conditions that are n=2). The box represents the 25-75 percentile range with the median plotted as horizontal bar; the whiskers are set to 10-90 percentile range. Dots outside the whiskers represent nuclei that are not in the 10-90 percentile range. *** $p<0.001$, ****, $p<0.0001$ (One-way ANOVA test).

C. Pol II ChIP in HeLa cells, treated with DMSO or CPT for the indicated times, on *β-actin* gene. Values are relative to in1 amplicon in DMSO-treated samples.

D. γH2AX ChIP in HeLa cells, treated with DMSO or CPT for 60 min, on *β-actin* gene. γH2AX values are normalized to total H2AX signal for each amplicon.

E. DIP in HeLa cells, transfected with control (shades of blue) or DHX9 #1 (shades of red) siRNA and treated with DMSO or CPT for 20 min, on *β-actin* gene. HeLa genomic DNA was untreated (-) or treated (+) with RNase H prior to IP with S9.6 antibody. Values are relative to in1 for each siRNA. The p-value is calculated for each condition for + RNase H samples versus the - RNase H samples.

Bars in C-E represent the average values from at least three independent experiments +/- SEM with
* $p<0.05$, ** $p<0.01$, *** $p<0.001$ (unpaired, two-tailed Student's t test).

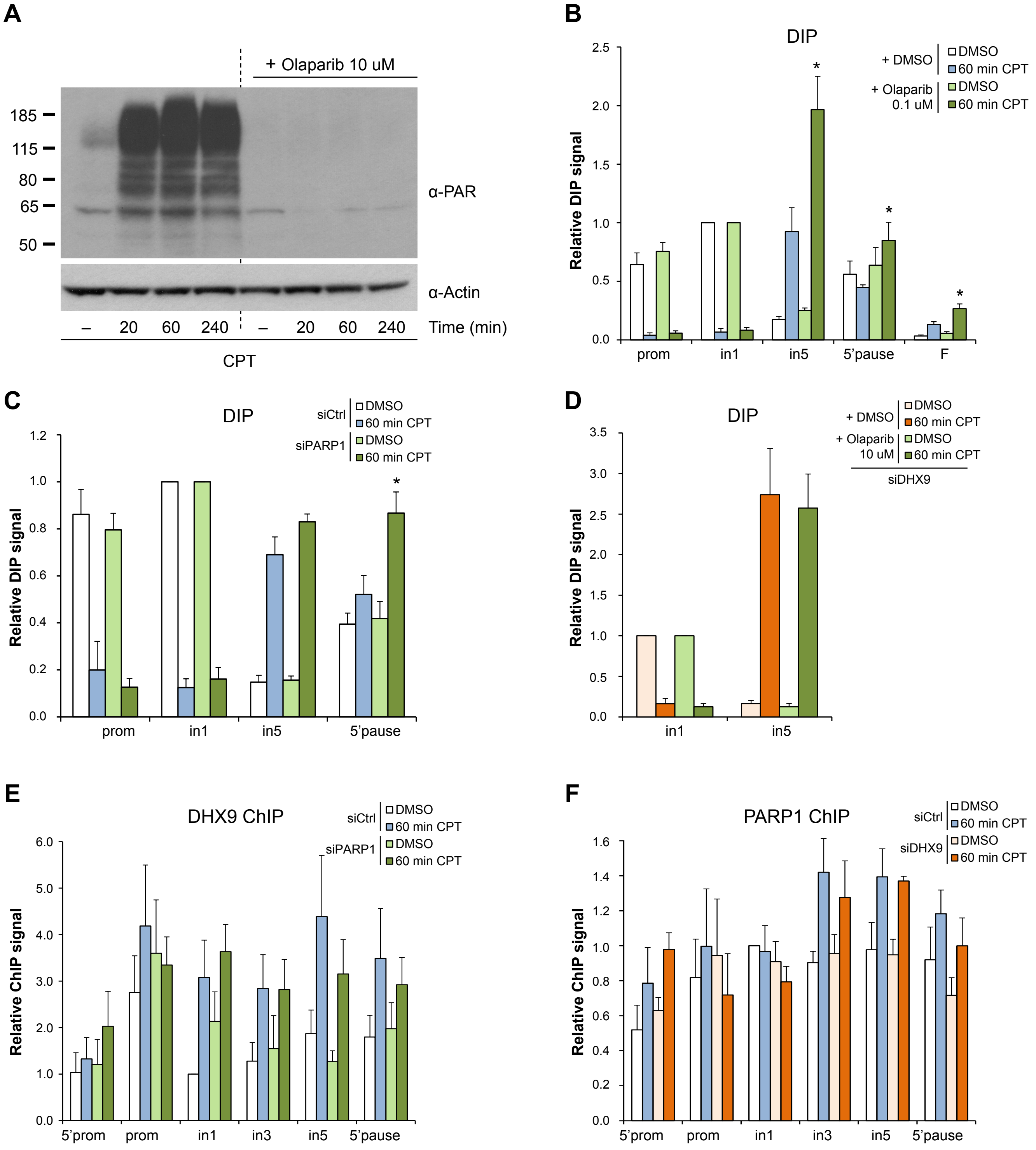
A
+ Olaparib 10 uM
185
115
80
65
50
α-PAR
α-Actin
– 20 60 240 – 20 60 240
Time (min)
CPT
B
DIP
+ DMSO
DMSO
60 min CPT
+ Olaparib 0.1 uM
DMSO
60 min CPT
Relative DIP signal
2.5
2.0
1.5
1.0
0.5
0.0
*
prom
in1
in5
5'pause
F
C
DIP
siCtrl
DMSO
60 min CPT
siPARP1
DMSO
60 min CPT
Relative DIP signal
1.2
1.0
0.8
0.6
0.4
0.2
0.0
*
prom
in1
in5
5'pause
D
DIP
+ DMSO
DMSO
60 min CPT
+ Olaparib 10 uM
DMSO
60 min CPT
siDHX9
Relative DIP signal
3.5
3.0
2.5
2.0
1.5
1.0
0.5
0.0
in1
in5
E
DHX9 ChIP
siCtrl
DMSO
60 min CPT
siPARP1
DMSO
60 min CPT
Relative ChIP signal
6.0
5.0
4.0
3.0
2.0
1.0
0.0
5'prom
prom
in1
in3
in5
5'pause
F
PARP1 ChIP
siCtrl
DMSO
60 min CPT
siDHX9
DMSO
60 min CPT
Relative ChIP signal
1.6
1.4
1.2
1.0
0.8
0.6
0.4
0.2
0.0
5'prom
prom
in1
in3
in5
5'pause

Figure S6. Effects of PARylation inhibition and PARP1 depletion on R-loops and DHX9. Related to Figure 5

A. Western blot of whole cells extracts from HeLa cells treated with DMSO or 10 uM Olaparib before the addition of DMSO or CPT for the indicated times. Blots were probed with anti-PAR antibody. Actin was used as loading control.

B. DIP analysis in HeLa cells, treated with DMSO (shades of blue) or 0.1 uM Olaparib (shades of green) before the addition of DMSO or CPT for 60 min, on *β-actin* gene. Values are relative to in1 for DMSO and Olaparib. The p-value is calculated for each amplicon for the Olaparib + CPT samples versus the DMSO + CPT samples.

C. DIP analysis in HeLa cells, transfected with control (siCtrl, shades of blue) and PARP1 siRNAs (shades of green) and treated with DMSO or CPT for 60 min, on *β-actin* gene. Values are relative to in1 for each siRNA. The p-value is calculated for the siPARP1 versus the siCtrl sample.

D. DIP analysis in HeLa cells, transfected with siRNA targeting DHX9 (DHX9 #1) and treated with DMSO or 10 uM Olaparib before the addition of DMSO or CPT for 60 min, on *β-actin* gene. Values are relative to in1 for DMSO and Olaparib.

E. DHX9 ChIP in HeLa cells, transfected with control (siCtrl, shades of blue) and PARP1 siRNAs (shades of green) and treated with DMSO or CPT for 60 min, on *β-actin* gene. Values are relative to in1 in siCtrl DMSO-treated samples.

F. PARP1 ChIP in HeLa cells (using anti-PARP1 from Proteintech #22999-1-AP), transfected with control (siCtrl, shades of blue) and DHX9 #1 siRNAs (shades of red) and treated with DMSO or CPT for 60 min, on *β-actin* gene. Values are relative to in1 in siCtrl DMSO-treated samples.

Bars in B-F represent the average values from at least three independent experiments +/- SEM with * $p<0.05$ (unpaired, two-tailed Student's t test).

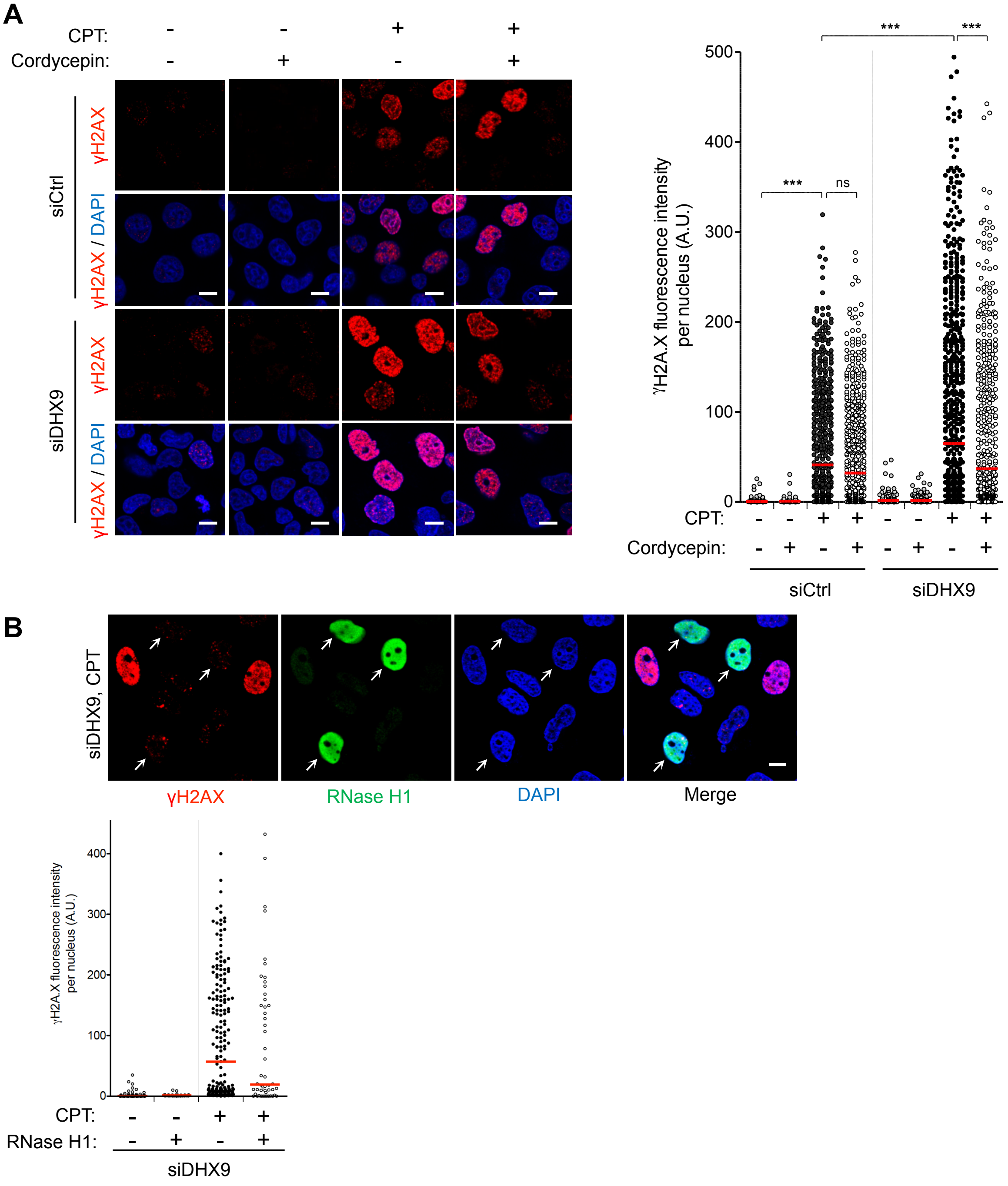

Figure S7. CPT-induced DNA damage in DHX9-depleted cells is transcription-dependent and decreases with RNase H1 overexpression. Related to Figure 6

A. IF analysis of HeLa cells transfected with DHX9 #1 or control siRNA and treated with Cordycepin before the addition of DMSO or CPT for 60 min and stained for γH2AX (red) and DAPI (blue). Left panel: representative images. Bars: 10 um. Right panel: γH2AX fluorescence intensity per nucleus from a representative experiment (≥ 300 nuclei were analyzed per condition). The horizontal red bar represents the means and each dot one nucleus.

B. IF analysis of HeLa cells transfected with DHX9 #1 siRNA and treated with DMSO or CPT for 60 min and then co-stained for γH2AX (red), RNase H1 (green) and DAPI (blue). Arrows indicate RNase H1 transfected cells. Top panel: representative images. Bars: 10 um. Bottom panel: γH2AX fluorescence intensity per nucleus from one representative experiment. Fluorescence was calculated for the cells with (+) or without (-) RNase H1 overexpression (green staining) in each condition. The horizontal red bar represents the median value of fluorescence and each dot corresponds to one nucleus.

Table S1: Full protein list of RNA/DNA hybrid interactome. Related to Figure 2

Table S2. Representative RNA/DNA hybrid interactors identified by MS. Related to Figure 2

Gene	Protein name	Enrichment	p-value	Class
**Transcription**				
DDX5	Probable ATP-dependent RNA helicase DDX5	10.34	1.32E-06	I
ZNF326	DBIRD complex subunit ZNF326	10.09	2.83E-06	I
CTCF	Transcriptional repressor CTCF	8.58	2.88E-06	I
MED19	Mediator of RNA polymerase II transcription subunit 19	6.35	8.20E-06	II
TTF1	Transcription termination factor 1	8.33	8.67E-06	II
**Splicing and Processing**				
SYNCRIP	Heterogeneous nuclear ribonucleoprotein Q	11.69	1.91E-06	I
SNRPE	Small nuclear ribonucleoprotein E	8.77	3.04E-06	I
PRPF19	Pre-mRNA-processing factor 19	10.89	3.45E-06	I
HNRNPA1	Heterogeneous nuclear ribonucleoprotein A1	7.50	4.03E-06	II
TRA2A	Transformer-2 protein homolog alpha	9.88	4.97E-06	II
SRPK1	SRSF protein kinase 1	7.07	1.10E-05	II
U2AF1	Splicing factor U2AF 65 kDa subunit	6.33	1.38E-05	II
SRSF9	Serine/arginine-rich splicing factor 9	7.51	2.60E-05	II
SNRNP70	U1 small nuclear ribonucleoprotein 70 kDa	9.47	1.66E-04	II
U2AF2	Splicing factor U2AF 65 kDa subunit	4.89	2.38E-04	III
**Epigenetic gene regulation**				
WHSC1	Histone-lysine N-methyltransferase NSD2	9.78	2.42E-06	I
HP1BP3	Heterochromatin protein 1-binding protein 3	10.27	3.04E-06	I
HDAC2	Histone deacetylase 2	8.09	5.17E-06	II
BAZ1B	Tyrosine-protein kinase BAZ1B	8.97	5.97E-06	II
MBD2	Methyl-CpG-binding domain protein 2	7.33	1.77E-05	II
NAT10	N-acetyltransferase 10	9.15	3.42E-05	II
KMT2A	Histone-lysine N-methyltransferase 2A	6.17	3.82E-05	II
CDYL	Chromodomain Y-like protein	5.63	3.87E-05	II
BRD7	Bromodomain-containing protein 7	6.73	6.04E-05	II
CBX3	Chromobox protein homolog 3	7.80	8.06E-05	II
RUVBL2	RuvB-like 2	5.40	1.58E-04	II
DNMT1	DNA (cytosine-5)-methyltransferase 1	4.99	1.71E-04	II
SUV39H1	Histone-lysine N-methyltransferase SUV39H1	4.01	1.25E-03	III
CBX5	Chromobox protein homolog 5	2.80	1.56E-03	III
SMARCA5	SWI/SNF-related matrix-associated actin-dependent regulator of chromatin subfamily A member 5	5.75	4.41E-03	III
**DNA replication and repair**				
TOP2A	DNA topoisomerase 2-alpha	7.82	7.76E-06	II

PRKDC	DNA-dependent protein kinase catalytic subunit	7.30	1.94E-05	II
PARP1	Poly [ADP-ribose] polymerase 1	6.47	3.42E-05	II
PARP2	Poly [ADP-ribose] polymerase 2	5.92	9.60E-05	II
PCNA	Proliferating cell nuclear antigen	4.37	1.42E-04	II
DDB1	DNA damage-binding protein 1	5.75	2.87E-04	III
XAB2	XPA Binding Protein 2	5.13	3.71E-04	III
MCM3	DNA replication licensing factor MCM3	3.31	2.81E-03	III

Table S3. RNA/DNA hybrid interactors identified by MS with known implication in R-loop biology in mammalian cells. Related to Figure 2

Gene	Protein name	Enrichment	p-value	Class	Notes	Reference
**Transcription**						
DHX9	ATP-dependent RNA helicase A	12.85	1.11E-06	I	*In vitro*	(Chakraborty and Grosse, 2011)
ILF3	Interleukin enhancer-binding factor 3	11.59	1.82E-06	I		(Nadel et al., 2015)
ILF2	Interleukin enhancer-binding factor 2	11.78	2.27E-06	I		(Nadel et al., 2015)
XRN2	5-3 exoribonuclease 2	9.82	4.10E-06	II		(Morales et al., 2016)
DDX1	ATP-dependent RNA helicase DDX1	8.29	4.94E-06	II		(Li et al., 2016; Li et al., 2008)
SUPT16H	FACT complex subunit SPT16	6.23	2.12E-04	II		(Herrera-Moyano et al., 2014)
SNW1	SNW domain-containing protein 1	8.26	2.39E-04	III		(Paulsen et al., 2009)
SSRP1	FACT complex subunit SSRP1	4.23	1.86E-03	III		(Herrera-Moyano et al., 2014)
**RNA processing and export**						
DDX21	Nucleolar RNA helicase	14.98	7.91E-07	I		(Song et al., 2017)
HNRNPC	Heterogeneous nuclear ribonucleoproteins C1/C2	10.56	1.29E-06	I		(Nadel et al., 2015)
SNRPD1	Small nuclear ribonucleoprotein Sm D1	10.95	1.32E-06	I		(Paulsen et al., 2009)
SNRPB	Small nuclear ribonucleoprotein-associated proteins B	9.33	1.87E-06	I		(Paulsen et al., 2009)
HNRNPU	Heterogeneous nuclear ribonucleoprotein U	14.91	3.62E-06	II		(Britton et al., 2014)

SNRPD3	Small nuclear ribonucleoprotein Sm D3	8.42	3.88E-06	II		(Paulsen et al., 2009)
SNRPA1	U2 small nuclear ribonucleoprotein A	8.51	4.03E-06	II		(Paulsen et al., 2009)
SNRNP40	U5 small nuclear ribonucleoprotein 40 kDa protein	7.91	8.88E-06	II		(Tresini et al., 2015)
FUS	RNA-binding protein FUS	7.94	1.23E-05	II		(Hill et al., 2016; Wang et al., 2015)
TARDBP	TAR DNA-binding protein 43	8.17	1.85E-05	II		(Hill et al., 2016)
PRPF8	Pre-mRNA-processing-splicing factor 8	10.44	2.10E-05	II		(Tresini et al., 2015)
DDX23	Probable ATP-dependent RNA helicase DDX23	6.97	2.11E-05	II		(Sridhara et al., 2017)
TARBP2	RISC-loading complex subunit TARBP2	5.69	3.24E-05	II	*In vitro*	(Vukovic et al., 2014)
TAF15	TATA-binding protein-associated factor 2N	5.10	8.7E-05	II	Direct function in R-loop biology is not determined	(Britton et al., 2014)
CRNKL1	Crooked neck-like protein 1	5.63	1.52E-04	II		(Paulsen et al., 2009)
CDC40	Pre-mRNA-processing factor 17	6.70	2.08E-04	II		(Paulsen et al., 2009)
SRPK2	SRSF protein kinase 2	6.08	2.58E-04	III		(Sridhara et al., 2017)
SRSF3	Serine/arginine-rich splicing factor 3	3.99	3.49E-04	III	*In vitro*	(Li and Manley, 2005)
SRSF2	Serine/arginine-rich splicing factor 2	3.68	5.58E-04	III		(Chen et al., 2018; Li and Manley, 2005)
SRSF1	Serine/arginine-rich splicing factor 1	3.03	9.84E-04	III		(Li and Manley, 2005; Tuduri et al., 2009)
SF3B2	Splicing factor 3B subunit 2	5.20	2.69E-03	III		(Tresini et al., 2015)
EIF4A	Eukaryotic initiation factor 4A	6.31	2.99E-04	III	*In vitro*	(Du et al., 2002)
FIP1L1	Pre-mRNA 3-end-processing factor FIP1	5.16	2.40E-04	III	Direct function in R-loop biology is not determined in human cells	(Stirling et al., 2012)

**DNA Topology**						
TOP1	DNA topoisomerase 1	4.61	1.83E-04	II		(Groh et al., 2014; Marinello et al., 2016; Marinello et al., 2013; Sollier et al., 2014; Sordet et al., 2009; Tuduri et al., 2009)
**Replication**						
MCM5	DNA replication licensing factor MCM5	5.00	3.71E-04	III	Murine B cell lines	(Wiedemann et al., 2016)
**Mitosis**						
BUB3	Mitotic checkpoint protein BUB3	5.52	3.29E-04	III		(Wan et al., 2015)
ZNF207	Zinc finger protein 207	4.71	4.14E-04	III		(Wan et al., 2015)

Supplemental experimental procedures

Oligonucleotide sequences

**Name**	**Sequence (5'⟶3')**
**β-actin gene**	
5'prom (F)	CCA CCT GGG TAC ACA CAG TCT
5'prom (R)	TGT CCT TGT CAC CCT TTC TTG
prom (F)	CCG AAA GTT GCC TTT TAT GGC
prom (R)	CAA AGG CGA GGC TCT GTG C
in1 (F)	CGG GGT CTT TGT CTG AGC
in1 (R)	CAG TTA GCG CCC AAA GGA C
in3(F)	TAA CAC TGG CTC GTG TGA CAA
in3(R)	AAG TGC AAA GAA CAC GGC TAA
in5(F)	GGA GCT GTC ACA TCC AGG GTC
in5(R)	TGC TGA TCC ACA TCT GCT GG
5'pause (F)	TTA CCC AGA GTG CAG GTG TG
5'pause (R)	CCC CAA TAA GCA GGA ACA GA
pause (F)	GGG ACT ATT TGG GGG TGT CT
pause (R)	TCC CAT AGG TGA AGG CAA AG
C (F)	TGG GCC ACT TAA TCA TTC AAC
C (R)	CCT CAC TTC CAG ACT GAC AGC
D (F)	CAG TGG TGT GGT GTG ATC TTG
D (R)	GGC AAA ACC CTG TAT CTG TGA

F (F)	CCA TCA CGT CCA GCC TAT TT
F (R)	TGT GTG AGT CCA GGA GTT GG
**γ-actin**	
prom (F)	GGA AAG ATC GCC ATA TAT GGA C
prom (R)	TCA CCG GCA GAG AAA CGC GAC
in1 (F)	CCG CAG TGC AGA CTT CCG AG
in1 (R)	CGG GCG CGT CTG TAA CAC GG
ex5(F)	GTG ACA CAG CAT CAC TAA GG
ex5 (R)	ACA GCA CCG TGT TGG CGT
A (F)	TTC GTG GGC TGG TGA GAA AA
A (R)	CTC CAA CAC CCA AAC CCA CT
B (F)	GGG TCA AGG GAT CGT TCT G
B (R)	GCC TGG AGC TCA GTA AGC
C (F)	GAG GTT TGA GAC TGC AGT GAG
C (R)	CAG ACA TAA TTT TGT GGG GTT TG
**Synthetic competitors for RNA/DNA hybrid IP**	
ssDNA (sense)	CGG TGT GAA TCA GAC
ssDNA (anti-sense)	GTC TGA TTC ACA CCG
ssRNA (sense)	CGG UGU GAA UCA GAC
ssRNA (anti-sense)	GUC UGA UUC ACA CCG
ds AU-rich RNA (sense)	AAU UAC AUU GAU AGA AUU AUU AG
ds AU-rich RNA (anti-sense)	CUA AUA AUU CUA UCA AUG UAA UU
**siRNA sequences**	All siRNAs are terminated by dTdT
control siRNA (siGENOME non-targeting siRNA#1, GE Life Science)	Sequence is licenced (D-001210-01)
DHX9 #1 siRNA (Thermo Fisher)	5'-GAAGUGCAAGCGACUCUAG-3'
DHX9 #2 siRNA (Thermo Fisher)	Sequence is licenced (s4019)
DHX9 #3 siRNA (GE Life Science)	Sequence is licenced (D-009950-01)
SETX siRNA (Invitrogen)	5'- AUUUGACGACGGCUUCCACCCAUUG-3'
Top1 siRNA (Thermo Fisher)	5'-GGACUCCAUCAGAUACUAU-3'
WHSC1 siRNA (Thermo Fisher)	5'- AACGGCCAGAACAAGCUCUUA -3'
SAFB2 siRNA (Thermo Fisher)	5'-GAGUCAGGAUCGCAAGUCA-3'
DNA-PK siRNA (Thermo Fisher)	5'-GGGCGCUAAUCGUACUGAA-3'
PARP1 siRNA (Thermo Fisher)	5'- GAAAGUGUGUUCAACUAAU-3'

siRNA and plasmid transfections

Transfection of plasmids and siRNAs was performed with Lipofectamine 2000 (Thermo Fisher Scientific) using the manufacturer's instructions. HeLa cells were transfected for 24 h and HEK293T cells for 48 h with pFlag (Sigma Aldrich, E7398), RNaseH1-Flag or RNaseH1-no tag plasmid (Groh et al., 2014). RNA interference was performed with Lipofectamine 2000 (Thermo Fisher Scientific) using the manufacturer's instructions. DHX9 and SETX siRNAs were transfected as described (Andersen et al., 2013).

Mass Spectrometry analysis

Peptides prepared using FASP were analysed on an Ultimate 3000 RSLCnano HPLC (Dionex, Camberley, UK) system run in direct injection mode coupled to a QExactive Orbitrap mass spectrometer (Thermo Electron, Hemel Hempstead, UK). Protein samples were resolved on a 25cm by 75 micron inner diameter picotip analytical column (New Objective, Woburn, MA, USA) which was packed in-house with ProntoSIL 120-3 C18 Ace-EPS phase, 3µm bead (Bischoff Chromatography, Germany). The system was operated at a flow-rate of 300nL min-1. A 120 min gradient was used to separate the peptides. The mass spectrometer was operated in a 'Top 20' data-dependent acquisition mode. Precursor scans were performed in the orbitrap at a resolving power of 70,000, from which the twenty most intense precursor ions were selected by the quadrupole and fragmented by HCD at a normalised collision energy of 30%. The quadrupole isolation window was set at 1.6 m/z. Charge state +1 ions and undetermined charge state ions were rejected from selection for fragmentation. Dynamic exclusion was enabled for 27s. Mass spectrometry data processing for all figures (except Figure S2B) was carried out using MaxQuant 1.5.0.35 and Andromeda search engine (Cox and Mann, 2008; Cox et al., 2011). Enzyme specificity was set to trypsin/P, allowing a maximum of two missed cleavages. Cysteine carbamidomethylation was selected as fixed and protein N-terminal acetylation and methionine oxidation as variable modifications. Initial mass tolerance of precursor ions was set to 50 ppm. Proteins and peptides were identified with FDR < 0.01 with a minimum peptide length of 7 amino acids. Protein identification required one unique peptide to the protein group. For protein quantification a minimum of two ratio counts were set and 'match between runs' function enabled. The initial 848 identified proteins were filtered for occurrence in at least three samples using Perseus 1.5.2.6. Common contaminants such as keratins and proteins of the large and small ribosomal subunits (RPL and RPS) were filtered out, due to their known contribution as contaminants and unresolved interactions in affinity purification procedures (Mellacheruvu et al., 2013).

For additional validation of the RNA/DNA hybrid interactome, an independent mass spectrometry quantitation pipeline was used, which is based on MS/MS spectra rather than ion intensities. For this, data were converted from .RAW to .MGF using ProteoWizard (Chambers et al., 2012). Data were analysed using the Central Proteomics Facility Pipeline software (Trudgian et al., 2010). Peptide searches were performed using the InterProphet meta-search combining Mascot, X! Tandem with the k-score plugin, and OMSSA against concatenated target/decoy sequence databases. Proteins were identified with at least two peptide sequences, with at least one unique peptide, FDR<1%. Relative label-free quantitation of proteins was carried out using the normalised spectral index implemented in the SINQ software (Trudgian et al., 2011).

The mass spectrometry proteomics data have been deposited to the ProteomeXchange Consortium via the PRIDE partner repository (Vizcaino et al., 2013) with the dataset identifier PXD002960 (www.ebi.ac.uk/pride/archive/login). Username: reviewer61059@ebi.ac.uk; Password: 3EpnlBv8.

Bioinformatical analyses

Statistical analysis of the mass spectrometry data from RNA/DNA hybrid IP is based on 3 independent biological replicates and was performed essentially as described elsewhere (Castello et al., 2012; Kwon et al., 2013). In short, intensity values were log_2-transformed and missing values were imputed with random numbers from a normal distribution to simulate low abundance values below noise level in Perseus 1.5.2.6, as described (Raschle et al., 2015). Using the limma package in R/Bioconductor, a linear model was fitted to these data to calculate the log_2 enrichment between RNA/DNA hybrid IP and control samples. An empirical Bayes moderated t-test was used to calculate p-values (Smyth, 2004) . P-values were then corrected for multiple testing by Benjamini-Hochberg method. Proteins enriched in RNA/DNA hybrid IP compared to control were included in the "RNA/DNA hybrid Interactome" if their corrected p-values < 0.01.

Cellular compartment analysis was based on GO term cellular component analysis. Fisher's exact test was used to calculate statistical enrichment, using Benjamini-Hochberg correction for multiple testing. Proteins were classified into the groups 'nucleus', 'nucleolus', 'nucleoplasm' and 'cytoplasm', with the latter group consisting of proteins that are exclusively cytoplasmic.

Overrepresentation analysis of protein classes was performed using the PANTHER database (www.pantherdb.org), version 10.0 (Mi et al., 2013). PANTHER protein classes overrepresented in the RNA/DNA hybrid IP were ranked according to their Benjamini-Hochberg-corrected p-values (p-value threshold 0.05) and the 17 most-significant groups were manually curated for redundancy.

Venn diagram representation of the overlap of RNA/DNA hybrid interactome with the HeLa mRNA interactome is based on (Castello et al., 2012). Two subgroups, proteins identified both in RNA/DNA hybrid interactome and HeLa interactome, and proteins exclusively identified in RNA/DNA hybrid interactome, were further examined for overrepresented protein classes using PANTHER, as described above.

Chromatin probability assignment was based on previously published data (Kustatscher et al., 2014) by assigning Interphase Chromatin Probability (ICP) values to the proteins in the RNA/DNA hybrid interactome. As comparison, ICP values are shown for the whole list of 7635 HeLa proteins as provided in (Kustatscher et al., 2014).

Genetic alterations of helicases identified in RNA/DNA hybrid interactome was carried out using the COSMIC database (cancer.sanger.ac.uk) (Forbes et al., 2015). In brief, total number of copy number variations (either gain or loss) were retrieved for each gene and expressed as percentage of total cancer samples annotated in the database, independently of the tissue. Data were plotted alongside two tumour suppressor genes and oncogenes. Differential mRNA expression analysis in cancer was performed using the ONCOMINE platform (www.oncomine.org) (Rhodes et al., 2007), with a p-value threshold of < 0.05 and a minimal fold-change of 2 between cancer and matched control samples. Cancer case studies with significant alterations were grouped based on whether the gene was amongst the top 1%, top 5%, or top 10% of all altered genes.

Antibodies

The following antibodies were used for ChIP: DHX9 (Abcam, ab26271, Lot#GR83942 and #187365), γH2AX (Millipore, 07-164), H2AX (Millipore, 07-627), H3 (Abcam, ab1791), PARP1 (Abcam ab6079, Proteintech 22999-1-AP) and Pol II (Santa Cruz, H-224). Antibodies used for IP, western blotting and IF: actin (Sigma, A2066), CBP80 (sc-48803, Santa Cruz), DDX5 (Bethyl, A300-523A), DDX1 (Proteintech, 11357-1-AP), DHX9 (Abcam, ab26271), DNA-PKcs (Abcam, ab1832), Drosha (Cell Signalling, D28B1), γH2AX (Millipore, 05-636), H3 (Abcam, ab1791), IgG2a (M5409, Sigma), Lamin B1 (Abcam, ab16048), Nuclear Pore Complex (Abcam, ab24609), anti-PAR (Trevigen, 4336-BPC-100), PARP1 (Abcam ab32138), RNA Pol II (Abcam, ab817), RNase H1 (Proteintech 156061-AP), SAFB2 (A301-113A-T), SETX (Bethyl, A301-105A), SRSF1 (LifeTechnologies, Clone 96), Topoisomerase I (Abcam, ab109374), alpha-tubulin (Sigma, T5168), WHSC1 (Abcam, ab75359), XRN2 (Proteintech, 11267-1-AP).

RNA/DNA hybrid slot blot

The slot blot was performed as described (Kotsantis et al., 2016; Sollier et al., 2014). RNase H sensitivity was carried out by incubation with 2 U of RNase H (NEB, M0297) per ug of genomic DNA for 2.5 h at 37 °C. Images were acquired with LAS-4000 (Fujifilm) (Figure 3) or by chemiluminescence using autoradiography in other figures. S9.6 signal was quantified using Image Studio Lite software (Li-COR Biosciences).

Immunoprecipitation and protein analysis

HeLa cells at 85% confluency were washed with PBS and lysed in RSB+T (10 mM TRIS pH 7.5, 200 mM NaCl, 2.5mM $MgCl2$, 0.5% Triton X-100) on ice, followed by brief sonication (Diagenode Bioruptor). After removal of insoluble material, 1 mg of extracts were incubated with 3 ug of antibodies overnight. Immuno-complexes were captured with protein A dynabeads (Invitrogen), washed in RSB+T and eluted as described for RNA/DNA hybrid IP.

To prepare whole cell extracts, cells were lysed in RIPA buffer or in 1% SDS and 10 mM Tris-HCl (pH 7.4) buffer as described (Cristini et al., 2016), supplemented with protease inhibitor cocktail (Roche). Silver staining of SDS-PAGE gels was carried as in (Green and Sambrook, 2012).

RNA/DNA hybrid IP with RNase H treatment

Genomic DNA containing RNA/DNA hybrids was isolated as described before (Groh et al., 2014) and treated with 5.5 U of RNase H (NEB, M0297) per ug of DNA overnight at 37 °C. DNA was sonicated for 10 min (Diagenode Bioruptor) prior to RNase H treatment for IP and western blot analysis or left unsonicated for the slot blot. A fraction of the genomic DNA was stored as 'genomic DNA Input' for the slot blot. Genomic DNA (4 ug for western blot and 30 ug for the slot blot) was enriched for RNA/DNA hybrids by immuno-precipitation with S9.6 antibody, bound to protein A dynabeads (Invitrogen), pre-blocked with 0.5% BSA/PBS, for 2 h at 4 °C. Beads were washed 3x with RSB+T and incubated for 2 h at 4 °C with diluted HeLa nuclear extracts containing 15 ug proteins, prepared as described for RNA/DNA hybrid IP and pre-treated with 0.1 mg/ml RNase A (PureLink, Invitrogen) for 1 h at 37 °C to degrade all RNA/DNA hybrids (Figure S3A). Excess of RNase A was blocked by adding 200 U of

RNasin (Promega) to IPs. 100 ul fraction of diluted and RNase A pre-treated extracts prior to IP was stored as 'Protein Input' for western blot. Bead washes and elution were performed as described for RNA/DNA hybrid IP.

Immunofluorescence microscopy (IF)

For DHX9 IF, cells were fixed with 3% PFA, washed, permeabilised and blocked in PBS, 1% goat serum, 0.5% triton X-100, followed by incubation with DHX9 antibody. γH2AX and RNase H1 IF were carried out as described (Cristini et al., 2016; Sordet et al., 2009). For S9.6 IF, cells were fixed with ice-cold methanol for 10 min at -20 °C, washed with PBS and permeabilized with Triton 0.1% for 10 min at RT. After PBS washes, slides were treated with 150 U/ml of RNase H (NEB, M0297) or left untreated for 36 h at 37 °C. After PBS washes, slides were blocked with 8% BSA before incubating with purified S9.6 antibody overnight at 4 °C. Slides were incubated with appropriate secondary antibodies, coupled to Alexa Fluor 488 or 594 (Invitrogen), and mounted using Vectashield with DAPI (Vector Laboratories). Images were acquired on an Axioplan 2e (Zeiss), on a confocal Olympus FV1200 or on a confocal Zeiss 880 Airyscan. Fluorescence intensities were quantified with ImageJ (version 1.50g or 1.51k).

RNA analysis

Total RNA was harvested using TRIZOL reagent (Invitrogen) followed by DNase I treatment (Roche). 1-2 µg of total RNA was reverse-transcribed using SuperScript Reverse Transcriptase III (Invitrogen) with random hexamers (Invitrogen) and analysed by quantitative PCR with QuantiTect SYBR green (Qiagen).

Supplemental References

Andersen, P.R., Domanski, M., Kristiansen, M.S., Storvall, H., Ntini, E., Verheggen, C., Schein, A., Bunkenborg, J., Poser, I., Hallais, M., *et al.* (2013). The human cap-binding complex is functionally connected to the nuclear RNA exosome. Nat Struct Mol Biol *20*, 1367-1376.

Chambers, M.C., Maclean, B., Burke, R., Amodei, D., Ruderman, D.L., Neumann, S., Gatto, L., Fischer, B., Pratt, B., Egertson, J., *et al.* (2012). A cross-platform toolkit for mass spectrometry and proteomics. Nat Biotechnol *30*, 918-920.

Chen, L., Chen, J.Y., Huang, Y.J., Gu, Y., Qiu, J., Qian, H., Shao, C., Zhang, X., Hu, J., Li, H., *et al.* (2018). The Augmented R-Loop Is a Unifying Mechanism for Myelodysplastic Syndromes Induced by High-Risk Splicing Factor Mutations. Molecular cell *69*, 412-425 e416.

Chen, E.Y., Tan, C.M., Kou, Y., Duan, Q., Wang, Z., Meirelles, G.V., Clark, N.R., and Ma'ayan, A. (2013). Enrichr: interactive and collaborative HTML5 gene list enrichment analysis tool. BMC Bioinformatics *14*, 128.

Cox, J., Neuhauser, N., Michalski, A., Scheltema, R.A., Olsen, J.V., and Mann, M. (2011). Andromeda: a peptide search engine integrated into the MaxQuant environment. J Proteome Res *10*, 1794-1805.

Du, M.X., Johnson, R.B., Sun, X.L., Staschke, K.A., Colacino, J., and Wang, Q.M. (2002). Comparative characterization of two DEAD-box RNA helicases in superfamily II: human translation-initiation factor 4A and hepatitis C virus non-structural protein 3 (NS3) helicase. Biochem J *363*, 147-155.

Green, M., and Sambrook, J. (2012). Molecular Cloning: A Laboratory Manual, 4th edn (Cold Spring Harbour Laboratory Press, Cold Spring Harbour, NY).

Hill, S.J., Mordes, D.A., Cameron, L.A., Neuberg, D.S., Landini, S., Eggan, K., and Livingston, D.M. (2016). Two familial ALS proteins function in prevention/repair of transcription-associated DNA damage. Proc Natl Acad Sci U S A *113*, E7701-E7709.

Kwon, S.C., Yi, H., Eichelbaum, K., Fohr, S., Fischer, B., You, K.T., Castello, A., Krijgsveld, J., Hentze, M.W., and Kim, V.N. (2013). The RNA-binding protein repertoire of embryonic stem cells. Nat Struct Mol Biol *20*, 1122-1130.

Li, L., Germain, D.R., Poon, H.Y., Hildebrandt, M.R., Monckton, E.A., McDonald, D., Hendzel, M.J., and Godbout, R. (2016). DEAD Box 1 Facilitates Removal of RNA and Homologous Recombination at DNA Double-Strand Breaks. Mol Cell Biol *36*, 2794-2810.

Li, L., Monckton, E.A., and Godbout, R. (2008). A role for DEAD box 1 at DNA double-strand breaks. Mol Cell Biol *28*, 6413-6425.

Marinello, J., Chillemi, G., Bueno, S., Manzo, S.G., and Capranico, G. (2013). Antisense transcripts enhanced by camptothecin at divergent CpG-island promoters associated with bursts of topoisomerase I-DNA cleavage complex and R-loop formation. Nucleic acids research *41*, 10110-10123.

Mellacheruvu, D., Wright, Z., Couzens, A.L., Lambert, J.P., St-Denis, N.A., Li, T., Miteva, Y.V., Hauri, S., Sardiu, M.E., Low, T.Y.*, et al.* (2013). The CRAPome: a contaminant repository for affinity purification-mass spectrometry data. Nat Methods *10*, 730-736.

Mi, H., Muruganujan, A., Casagrande, J.T., and Thomas, P.D. (2013). Large-scale gene function analysis with the PANTHER classification system. Nat Protoc *8*, 1551-1566.

Nadel, J., Athanasiadou, R., Lemetre, C., Wijetunga, N.A., P, O.B., Sato, H., Zhang, Z., Jeddeloh, J., Montagna, C., Golden, A.*, et al.* (2015). RNA:DNA hybrids in the human genome have distinctive nucleotide characteristics, chromatin composition, and transcriptional relationships. Epigenetics Chromatin *8*, 46.

Raschle, M., Smeenk, G., Hansen, R.K., Temu, T., Oka, Y., Hein, M.Y., Nagaraj, N., Long, D.T., Walter, J.C., Hofmann, K.*, et al.* (2015). DNA repair. Proteomics reveals dynamic assembly of repair complexes during bypass of DNA cross-links. Science *348*, 1253671.

Sollier, J., Stork, C.T., Garcia-Rubio, M.L., Paulsen, R.D., Aguilera, A., and Cimprich, K.A. (2014). Transcription-coupled nucleotide excision repair factors promote R-loop-induced genome instability. Molecular cell *56*, 777-785.

Song, C., Hotz-Wagenblatt, A., Voit, R., and Grummt, I. (2017). SIRT7 and the DEAD-box helicase DDX21 cooperate to resolve genomic R loops and safeguard genome stability. Genes & development.

Sridhara, S.C., Carvalho, S., Grosso, A.R., Gallego-Paez, L.M., Carmo-Fonseca, M., and de Almeida, S.F. (2017). Transcription Dynamics Prevent RNA-Mediated Genomic Instability through SRPK2-Dependent DDX23 Phosphorylation. Cell Rep *18*, 334-343.

Tresini, M., Warmerdam, D.O., Kolovos, P., Snijder, L., Vrouwe, M.G., Demmers, J.A., van, I.W.F., Grosveld, F.G., Medema, R.H., Hoeijmakers, J.H.*, et al.* (2015). The core spliceosome as target and effector of non-canonical ATM signalling. Nature *523*, 53-58.

Trudgian, D.C., Thomas, B., McGowan, S.J., Kessler, B.M., Salek, M., and Acuto, O. (2010). CPFP: a central proteomics facilities pipeline. Bioinformatics *26*, 1131-1132.

Vizcaino, J.A., Cote, R.G., Csordas, A., Dianes, J.A., Fabregat, A., Foster, J.M., Griss, J., Alpi, E., Birim, M., Contell, J.*, et al.* (2013). The PRoteomics IDEntifications (PRIDE) database and associated tools: status in 2013. Nucleic acids research *41*, D1063-1069.

Vukovic, L., Koh, H.R., Myong, S., and Schulten, K. (2014). Substrate recognition and specificity of double-stranded RNA binding proteins. Biochemistry *53*, 3457-3466.

Wan, Y., Zheng, X., Chen, H., Guo, Y., Jiang, H., He, X., Zhu, X., and Zheng, Y. (2015). Splicing function of mitotic regulators links R-loop-mediated DNA damage to tumor cell killing. J Cell Biol *209*, 235-246.

Wang, X., Schwartz, J.C., and Cech, T.R. (2015). Nucleic acid-binding specificity of human FUS protein. Nucleic acids research *43*, 7535-7543.

Wiedemann, E.M., Peycheva, M., and Pavri, R. (2016). DNA Replication Origins in Immunoglobulin Switch Regions Regulate Class Switch Recombination in an R-Loop-Dependent Manner. Cell Rep *17*, 2927-2942.
